# RNA-seq analysis of the influence of anaerobiosis and FNR on *Shigella flexneri*

**DOI:** 10.1186/1471-2164-15-438

**Published:** 2014-06-06

**Authors:** Marta Vergara-Irigaray, Maria C Fookes, Nicholas R Thomson, Christoph M Tang

**Affiliations:** Sir William Dunn School of Pathology, Oxford University, Oxford, United Kingdom; Centre for Molecular Microbiology and Infection, Imperial College London, London, United Kingdom; The Wellcome Trust Sanger Institute, Hinxton, Cambridgeshire United Kingdom

## Abstract

**Background:**

*Shigella flexneri* is an important human pathogen that has to adapt to the anaerobic environment in the gastrointestinal tract to cause dysentery. To define the influence of anaerobiosis on the virulence of *Shigella*, we performed deep RNA sequencing to identify transcriptomic differences that are induced by anaerobiosis and modulated by the anaerobic Fumarate and Nitrate Reduction regulator, FNR.

**Results:**

We found that 528 chromosomal genes were differentially expressed in response to anaerobic conditions; of these, 228 genes were also influenced by FNR. Genes that were up-regulated in anaerobic conditions are involved in carbon transport and metabolism (*e.g. ptsG, manX, murQ, cysP, cra*), DNA topology and regulation (*e.g. ygiP, stpA, hns*), host interactions (*e.g. yciD, nmpC, slyB, gapA, shf, msbB*) and survival within the gastrointestinal tract (*e.g. shiA, ospI, adiY, cysP*). Interestingly, there was a marked effect of available oxygen on genes involved in Type III secretion system (T3SS), which is required for host cell invasion and pathogenesis. These genes, located on the large *Shigella* virulence plasmid, were down regulated in anaerobiosis in an FNR-dependent manner. We also confirmed anaerobic induction of csrB and csrC small RNAs in an FNR-independent manner.

**Conclusions:**

Anaerobiosis promotes survival and adaption strategies of *Shigella*, while modulating virulence plasmid genes involved in T3SS-mediated host cell invasion. The influence of FNR on this process is more extensive than previously appreciated, although aside from the virulence plasmid, this transcriptional regulator does not govern expression of genes on other horizontally acquired sequences on the chromosome such as pathogenicity islands.

**Electronic supplementary material:**

The online version of this article (doi:10.1186/1471-2164-15-438) contains supplementary material, which is available to authorized users.

## Background

*Shigella flexneri* is a Gram-negative bacterium that causes dysentery, an acute human rectocolitis that usually results in destruction of the intestinal mucosa and bloody diarrhoea. The ability of this pathogen to invade epithelial cells at the colonic and rectal mucosal surface is a key determinant in the establishment of the disease. This is mediated by a Type III secretion system (T3SS) encoded on the large *Shigella* virulence plasmid [1, 2]. The T3SS acts like a molecular syringe that delivers molecules directly from the bacterial cytoplasm into host cells via a needle-like structure [1, 2]. However, before the bacterium reaches the large intestine and invades mucosal epithelial cells, *Shigella* must successfully survive the hostile conditions found in the gastrointestinal tract. Therefore the capacity of the bacterium to adapt to anaerobiosis, changes in pH, resist antimicrobial peptides, and acquire nutrients is essential for its pathogenesis [3, 4].

Anaerobiosis is known to influence the virulence of several enteric pathogens including *Shigella*, *Escherichia coli*, *Salmonella* spp., *Vibrio cholerae* and *Yersinia enterocolitica*[5–13]. In particular, *S. flexneri* has been shown to be primed for invasion in anaerobic conditions, in which it expresses longer T3SS needles while reducing Ipa (invasion plasmid antigen) effector secretion; this results from FNR-mediated repression of the virulence plasmid genes, *spa32* and *spa33*[7]. FNR is a major regulator of anaerobic metabolism that is inactivated by the presence of oxygen. Its function depends on the integrity of its O_2_-sensitive [4Fe–4S] cluster, which is required for FNR dimerization and thence site-specific DNA binding and transcriptional regulation [14]. One RNA deep sequencing (RNA-seq) and several microarray studies have been performed to characterise the extent of the FNR regulon in *E. coli* and other Gram negative pathogens such as *Salmonella enterica* and *Neisseria gonorrhoeae*[15–20]. In *E. coli*, there were significant discrepancies between studies even when the same strain was examined. However some differences could be attributed to the use of media containing high levels of glucose, which represses expression from some FNR-activated promoters, and the delayed growth rate of mutants lacking FNR compared with wild-type strains under anaerobic conditions [16].

Here we define the regulatory role of oxygen and FNR in *S. flexneri*. We have applied two powerful whole-transcriptome approaches, RNA-seq complemented with Flow cell Reverse Transcription sequencing (FRT-seq), in which there is no amplification during library preparation, to quantify differences in gene expression induced by anaerobiosis and to define the contribution of FNR in this process. We found that *Shigella* grown anaerobically exhibits global transcriptional changes compared to when grown aerobically, with marked changes in metabolic and transport genes, as well as those involved in regulatory and virulence functions. Importantly, transcription from the *Shigella* virulence plasmid is extensively modified in anaerobiosis, with most of T3SS-related genes being down regulated in the absence of oxygen in an FNR-dependent manner, demonstrating that this highly conserved regulator of metabolism also controls the horizontally-acquired virulence genes on the plasmid, but not on the chromosome, in this important human pathogen.

## Results

### Growth conditions and RNA sequencing strategies

To determine the response of *Shigella* to anaerobiosis and the role of FNR in this process, we employed RNA-seq to compare the transcriptional profiles of wild type *S. flexneri* M90T and its Δ*fnr* mutant grown in Luria-Bertani (LB) medium in the presence and absence of oxygen. Constantinidou *et al*. designed a supplemented, minimal salts medium (including LB) in which an *E. coli fnr* mutant exhibited similar growth as the parental strain in the absence of oxygen [16]. However, this medium did not support the growth of *S. flexneri* M90T. On the other hand, enriched-glucose media have been shown to repress some FNR-activated promoters [16]. Therefore, we chose LB with no added glucose for our experiments. Particular attention was paid to ensure that the culture volume, agitation, temperature and the growth stage of bacteria did not differ in aerobic and anaerobic conditions. Cultures were grown to an Optical Density at 600 nm (OD_600_) of 0.2 to avoid a reduction in the concentration of dissolved oxygen tension and total depletion of sugars that occurs during exponential growth [21, 22]. Furthermore until reach OD_600_ of 0.2 under anaerobiosis, there was no obvious delay in growth rate of the Δ*fnr* mutant in relation to the wild-type strain (See Additional file 1: Figure S1). Three biological replicates were performed per strain in each condition, and differential expression between conditions was analysed with the *DESeq* R statistical package.

To assess the reproducibility of results obtained with RNA-seq data and to further characterise the role of FNR, the *Shigella* FNR regulon under anaerobiosis was also examined using FRT-seq, an alternative sequencing approach in which cDNA synthesis is performed on the sequencing flowcell thereby avoiding the possible PCR biases generated during library preparation using standard RNA-seq methods [23]. FRT-seq confirmed 77% of the genes found differentially expressed by RNA-seq, showing a robust concordance between the two techniques. Due to its higher sensitivity, FRT-seq detected more genes whose transcription was significantly influenced by the absence of FNR than RNA-seq (See Additional file 1: Table S2). A complete catalogue of significant differences is shown in Additional material (See Additional file 1: Tables S1 and S2) as well as a summary of the mapping statistics (See Additional file 1: Table S3). To confirm the results obtained by global analysis of the transcriptional profile, we performed strand-specific qRT-PCR to analyse mRNA levels of several genes found to be differentially expressed under anaerobic and aerobic growth conditions.

### Identification of novel chromosomal genes influenced by the absence of oxygen in *S. flexneri*

Analysis of the RNA-seq data revealed that 528 chromosomal genes were differentially expressed by wild-type *S. flexneri* M90T grown under anaerobic conditions compared with aerobic conditions, with 363 genes being up-regulated, and 165 genes down-regulated. Additional file 1: Table S1 shows these genes classified into functional categories based on the database of Clusters of Orthologous Groups (COGs) [24]. As expected, most of the genes differentially expressed were related to energy production and metabolism (53%). The remaining genes were involved in cellular processes and signalling (15%), information storage and processing (8%) or were poorly characterized (24%). RNA-seq data also showed that from the above 528 differentially expressed genes, 228 genes (43%) were influenced by the absence of FNR under anaerobic conditions (See Additional file 1: Table S1).

Importantly the majority of genes that we found to be anaerobically induced/repressed have been identified in previous microarray studies with other enteric pathogens examining the effect of oxygen on the transcriptome and/or the two main anaerobic regulators, FNR and ArcA [6, 16–20, 25]. Consistent with previous work, we found increased expression of genes involved in anoxic carbon metabolism (*focA-pfl, yfiD, fdnG, gldA, aspA, fumB, ansB*), respiratory pathways (*glpABC*, *nap*, *nir, ccm, nrfABC, frd*), production of hydrogenases (*hyb, hya, hyc, hyp)*, fermentation (*adhE, ackA-pta, fdhF*) and acid response (*adiA, adiY, yjdE, gadA, hdeAB*) under anaerobiosis (Additional file 1: Table S1) [6, 16–18, 20, 25, 26]. Our analysis also identified several anaerobically repressed genes that have been previously characterised [6, 16–20, 25]. These genes encode enzymes of the tricarboxylic acid cycle (*ace, gltA, acn, icdA, sdh*), aerobic dehydrogenases (*glpD, betBA, gcd, aldA*), transhydrogenases (*udhA*) and iron acquisition systems (*exb, iuc, iutA, sit, suf, fep, fhu*), and others (Additional file 1: Table S1) [6, 16–18, 20, 25, 27, 28].

**Figure 1:**
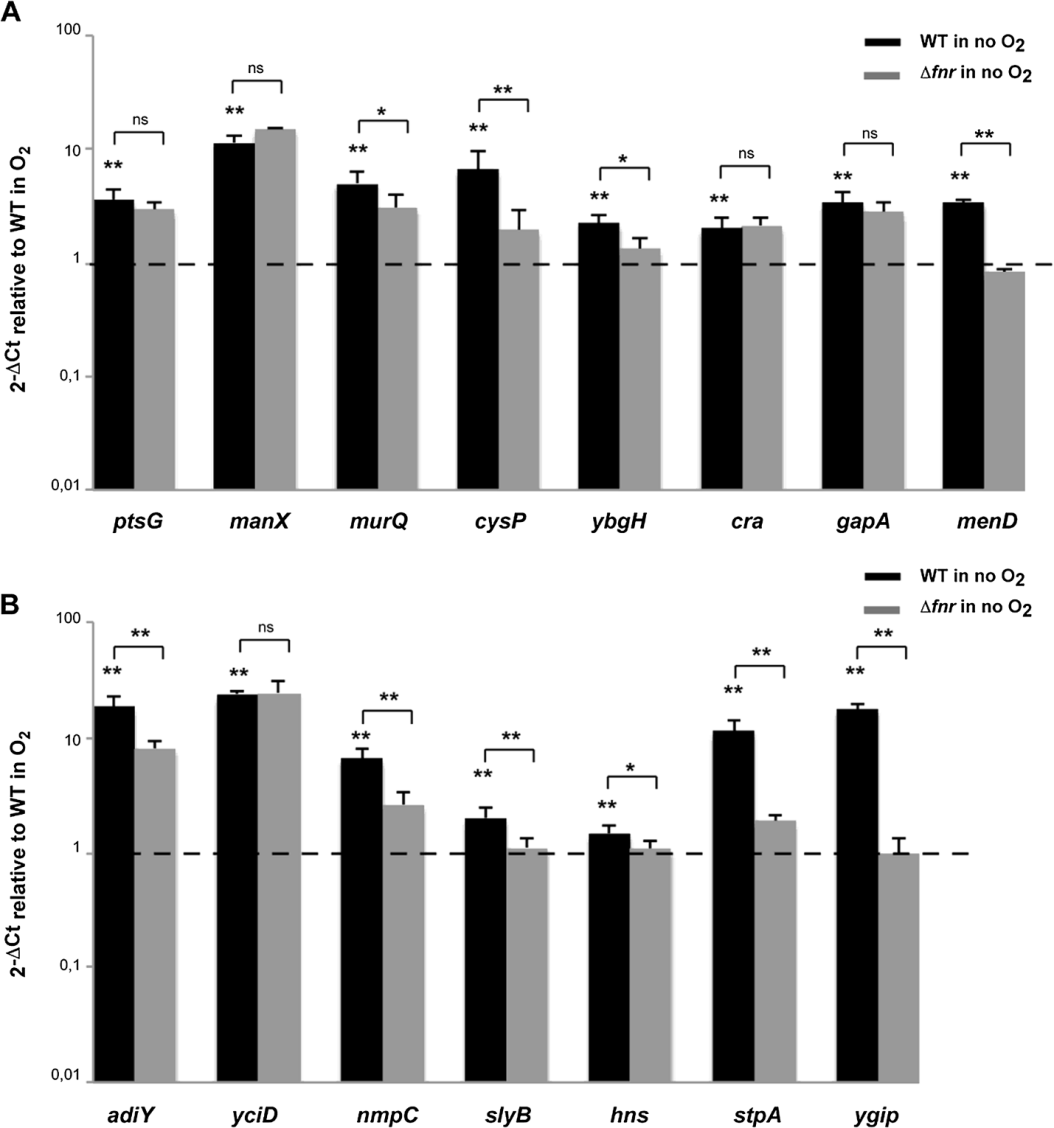
**qRT-PCR verification of** ***S. flexneri*** **chromosomal genes induced under anaerobic growth conditions and the role of FNR in the process.** Strand specific qRT-PCR analysis of mRNA levels of *S. flexneri* M90T chromosomal genes shown to be induced under anaerobiosis in RNA-seq analysis. Panel **A** shows transport and metabolic genes, and panel **B** acid resistance, OMP and regulatory genes. Data were calculated as the n-fold difference relative to *polA* (2^-Δ*Ct*^, where Δ*Ct* represents the difference in threshold cycle between the target and control genes). Results are shown in relation to the wild-type strain 2^-Δ*Ct*^ levels under aerobic conditions, here referred to as 1. Thus, values greater than 1 indicate increased transcription under anaerobiosis, and lower than 1 indicate the opposite. Significant differences were detected when wild-type 2^-Δ*Ct*^ levels under aerobic and anaerobic conditions, or wild-type *vs.* Δ*fnr* 2^-Δ*Ct*^ levels under anaerobiosis were compared. ns = non-significant, *P* < 0.05, *; *P* <0.01, **; n = 4; Mann–Whitney test. Error bars show Standard Deviation (SD).

The sensitivity of the direct sequencing approaches, RNA-seq and FRT-seq, compared with array-based methods enabled us to extend the repertoire of *Shigella* genes modulated by ambient oxygen. Table 1 shows all genes influenced by the presence of oxygen and not detected in previous microarray studies on *E. coli* and *S. flexneri*[6, 16–18, 20, 25]. The effect of FNR mutation on the transcription of previous genes under anaerobiosis (assessed by RNA-seq and FRT-seq) is also shown in Table 1. Several members of the phosphoenolpyruvate–carbohydrate phosphotransferase system (PTS), involved in the transport and phosphorylation of sugars, were up-regulated under anaerobic conditions. Examples include *ptsHI*, which encode the general PTS components phosphohistidine carrier protein (HPr) and Enzyme I (EI) respectively, and sugar-specific PTS components like *ptsG* and *manXYZ* (involved in glucose transport), *treBC* (trehalose transport and hydrolysis), *mtlA* (mannitol) and *murQP* that contribute to the uptake and catabolism of *N*-acetylmuramic acid [29–32]. Of note, the *murQP* operon, which is also involved in peptidoglycan recycling, showed an FNR-dependent expression pattern (Table 1, Figure 1A) [31].

**Table 1:** **Chromosomal genes differentially expressed in response to anaerobic conditions not previously published in** ***E. coli*** **and** ***S. flexneri*** **microarray analysis**

ORF ID ^ab^	Gene	Description	RNA-seq ^c^ log2FC	RNA-seq ^c^ log2FC	FRT-seq ^c^ log2FC
			WT no O _2_ /O _2_	Δ ***fnr*** /WT no O _2_	Δ ***fnr*** /WT no O _2_
***Metabolism***					
Energy production and conversion					
SF5M90T_1519		putative oxidoreductase, major subunit	**3.80**	**-4.97**	**-3.30**
SF5M90T_3856	*yiaY*	putative oxidoreductase	**3.18**	**-2.73**	**-2.67**
SF5M90T_1560		putative oxidoreductase, major subunit	**3.06**		**-3.87**
SF5M90T_3333	*pckA*	phosphoenolpyruvate carboxykinase	**1.99**		**0.62**
SF5M90T_3877	*yiaK*	putative dehydrogenase	**1.93**	**1.22**	**0.93**
SF5M90T_3374	*ugpQ*	glycerophosphodiester phosphodiesterase, cytosolic	**1.89**		
SF5M90T_2534	*hmpA*	dihydropteridine reductase, ferrisiderophore reductase activity	**1.47**	**5.38**	**5.85**
SF5M90T_33	*caiB*	l-carnitine dehydratase	**1.32**		
SF5M90T_3679	*atpF*	membrane-bound ATP synthase, F0 sector, subunit b	**1.04**		
SF5M90T_3680	*atpE*	membrane-bound ATP synthase, F0 sector, subunit c	**1.04**		
SF5M90T_3937	*ppc*	phosphoenolpyruvate carboxylase	**0.91**		**0.64**
SF5M90T_579	*galT*	galactose-1-phosphate uridylyltransferase	**0.77**		
SF5M90T_1419	*ydjA*	predicted oxidoreductase	**-1.17**	**1.69**	**1.52**
SF5M90T_2771	*ygaF*	hydroxyglutarate oxidase	**-1.31**	**4.12**	**2.99**
SF5M90T_4044	*gltP*	glutamate-aspartate symport protein	**-1.32**		
SF5M90T_1603	*rnfB*	electron transport complex protein	**-1.46**		
SF5M90T_2869	*fldB*	flavodoxin 2	**-1.56**		
SF5M90T_1602	*rnfA*	Na + -translocating NADH-quinone reductase subunit E	**-1.75**		
SF5M90T_1011	*rutA*	pyrimidine monooxygenase	**-3.07**		
Carbohydrate transport and metabolism					
SF4250	*treB*	PTS system trehalose(maltose)-specific transporter subunits IIBC	**3.66**		
SF5M90T_4160	*treC*	trehalase 6-P hydrolase	**3.56**		
SF5M90T_1379	*manX*	PTS enzyme IIAB, mannose-specific	**3.36**		
SF5M90T_1378	*manY*	PTS enzyme IIC, mannose-specific	**3.11**		
SF5M90T_1377	*manZ*	PTS enzyme IID, mannose-specific	**2.89**		
SF5M90T_3670	*rbsD*	high affinity ribose transport protein	**2.71**		
SF5M90T_1101	*ptsG*	PTS system, glucose-specific IIBC component	**2.27**		
SF5M90T_3491	*treF*	cytoplasmic trehalase	**2.12**		
SF5M90T_2419	*murP*	PTS system N-acetylmuramic acid transporter subunits EIIBC	**2.09**		**-0.71**
SF5M90T_3499	*pfkA*	6-phosphofructokinase I	**2.08**		
SF5M90T_2096		fructose-bisphosphate aldolase	**2.02**	**2.08**	**1.86**
SF5M90T_1001	*agp*	periplasmic glucose-1-phosphatase	**2.00**	**1.40**	**1.57**
SF5M90T_2887	*rpiA*	ribosephosphate isomerase, constitutive	**1.84**		
SF5M90T_2898	*pgk*	phosphoglycerate kinase	**1.84**		
SF5M90T_1403	*gapA*	glyceraldehyde-3-phosphate dehydrogenase A	**1.83**		
SF5M90T_2097	*yegT*	putative nucleoside permease protein	**1.74**		
SF5M90T_2897	*fba*	fructose-bisphosphate aldolase, class II	**1.56**		
SF5M90T_2404	*ptsH*	PTS system protein HPr	**1.56**		
SF5M90T_3850	*mtlA*	PTS system, mannitol-specific enzyme IIABC components	**1.52**		
SF5M90T_1640	*ydhC*	putative transport protein	**1.51**		**0.69**
SF5M90T_2359		beta-fructosidase	**1.49**		**0.72**
SF5M90T_3496	*tpiA*	triosephosphate isomerase	**1.45**		
SF5M90T_2405	*ptsI*	PEP-protein phosphotransferase system enzyme I	**1.42**		
SF5M90T_2808	*fucI*	L-fucose isomerase	**1.41**		
SF5M90T_2875	*bglA*	6-phospho-beta-glucosidase A	**1.27**		
SF5M90T_3348	*malP*	maltodextrin phosphorylase	**1.16**		**0.78**
SF5M90T_1107	*ycfO*	beta-hexosaminidase	**1.13**		
SF5M90T_8	*talB*	transaldolase B	**1.10**		
SF5M90T_2033	*gnd*	gluconate-6-phosphate dehydrogenase	**1.01**		
SF5M90T_581	*galM*	galactose-1-epimerase	**1.01**	**1.41**	**1.00**
SF5M90T_1805	*eda*	keto-hydroxyglutarate-aldolase/keto-deoxy-phosphogluconate aldolase	**0.97**		
SF5M90T_580	*galK*	galactokinase	**0.95**		**0.62**
SF5M90T_2913	*tktA*	transketolase 1 isozyme	**0.80**		
SF5M90T_2187	*fruB*	PTS system fructose-specific transporter subunit IIA/HPr protein	**-1.33**		
SF5M90T_2186	*fruK*	fructose-1-phosphate kinase	**-1.75**		
SF5M90T_3161	*ptsO*	phosphocarrier protein NPr	**-1.76**		
SF5M90T_1637		putative transport protein	**-1.93**	**1.58**	
SF5M90T_2185	*fruA*	PTS system, fructose-specific transport protein	**-1.99**		
Aminoacid transport and metabolism					
SF5M90T_2823	*argA*	N-acetylglutamate synthase	**1.94**		
SF5M90T_1910	*fliY*	putative periplasmic binding transport protein	**1.80**		
SF5M90T_625	*ybgH*	peptide transporter	**1.64**	**-1.60**	**-1.21**
SF5M90T_292	*pepD*	aminoacyl-histidine dipeptidase (peptidase D)	**1.54**	**1.78**	**1.75**
SF5M90T_2879	*gcvT*	aminomethyltransferase	**1.53**		**1.44**
SF5M90T_284	*proA*	gamma-glutamylphosphate reductase	**1.48**		
SF5M90T_1121	*potD*	spermidine/putrescine periplasmic transport protein	**1.44**		**-0.69**
SF5M90T_2674	*cysD*	ATP:sulfurylase (ATP:sulfate adenylyltransferase), subunit 2	**1.39**	**-2.30**	**-2.21**
SF5M90T_1514	*dcp*	dipeptidyl carboxypeptidase II	**1.35**		**0.63**
SF5M90T_285	*proB*	gamma-glutamate kinase	**1.26**		**-0.58**
SF5M90T_2533	*glyA*	serine hydroxymethyltransferase	**1.16**		
SF5M90T_2967	*gsp*	glutathionylspermidine synthetase/amidase	**1.16**		**0.70**
SF5M90T_1806	*edd*	6-phosphogluconate dehydratase	**1.15**		**-0.72**
SF5M90T_807		glutathione transporter ATP-binding protein	**1.08**		
SF5M90T_2317	*hisJ*	histidine-binding periplasmic protein of high-affinity histidine transport system	**1.05**		
SF5M90T_806	*ybiK*	putative asparaginase	**1.02**		
SF5M90T_1122	*potC*	spermidine/putrescine transport system permease	**0.97**	**-1.09**	**-0.92**
SF5M90T_2882	*pepP*	proline aminopeptidase P II	**0.94**		
SF5M90T_2877	*gcvP*	glycine decarboxylase	**0.90**	**2.13**	**1.69**
SF5M90T_3687	*asnA*	asparagine synthetase A	**-1.23**		**-1.61**
SF5M90T_4099	*lysC*	aspartokinase III, lysine sensitive	**-1.33**	**1.37**	**1.14**
SF5M90T_1253	*trpE*	anthranilate synthase component I	**-1.57**		
SF5M90T_4187	*cycA*	transport of D-alanine, D-serine, and glycine	**-1.69**		
SF5M90T_1946	*yedA*	putative transmembrane subunit	**-1.79**		
SF5M90T_3626	*yifK*	putative amino acid/amine transport protein	**-1.94**		
SF5M90T_3385	*livJ*	Leu/Ile/Val-binding protein precursor	**-1.95**		
SF5M90T_2843	*lysA*	diaminopimelate decarboxylase	**-2.11**	**2.44**	
SF5M90T_4029	*proP*	low-affinity transport system; proline permease II	**-2.65**	**1.05**	**0.75**
SF5M90T_4185	*ytfF*	putative transmembrane subunit	**-3.70**	**2.71**	
Nucleotide transport and metabolism					
SF5M90T_3587	*udp*	uridine phosphorylase	**3.12**		**0.59**
SF5M90T_2387	*nupC*	permease of transport system for 3 nucleosides	**2.81**		
SF5M90T_2949	*nupG*	nucleoside permease	**2.47**		
SF5M90T_674	*ybeK*	putative tRNA synthetase	**1.60**		
SF5M90T_444	*adk*	adenylate kinase	**1.51**		**-0.67**
SF5M90T_2456	*purC*	phosphoribosylaminoimidazole-succinocarboxamidesynthetase	**1.35**		
SF5M90T_4182	*cpdB*	2′:3′-cyclic-nucleotide 2′-phosphodiesterase	**1.16**		
SF5M90T_291	*gpt*	guanine-hypoxanthine phosphoribosyltransferase	**1.10**		**-0.89**
SF5M90T_1598	*add*	adenosine deaminase	**0.95**		
SF5M90T_478	*purE*	phosphoribosylaminoimidazole carboxylase	**-1.41**		
Coenzyme transport and metabolism					
SF5M90T_2274	*menB*	dihydroxynaphtoic acid synthetase	**2.63**	**-1.75**	**-1.65**
SF5M90T_2687		phenylacrylic acid decarboxylase-like protein	**1.83**		
SF5M90T_2276	*menD*	2-oxoglutarate decarboxylase	**1.76**	**-1.63**	**-1.75**
SF5M90T_2273	*menC*	O-succinylbenzoate synthase	**1.76**	**-1.72**	**-1.57**
SF5M90T_3142	*ispB*	octaprenyl diphosphate synthase	**1.06**		
SF5M90T_1613	*pdxH*	pyridoxinephosphate oxidase	**1.06**		
SF5M90T_2880	*visC*	putative FAD-dependent oxidoreductase	**0.89**		
SF5M90T_3011	*ribB*	3,4 dihydroxy-2-butanone-4-phosphate synthase	**-1.10**		
SF5M90T_3577	*yigC*	putative oxidoreductase	**-1.31**		
SF5M90T_2885	*ygfA*	putative ligase	**-1.59**		
SF5M90T_3957	*birA*	biotin--protein ligase	**-1.62**		**-0.51**
SF5M90T_2103	*thiM*	hydoxyethylthiazole kinase	**-1.95**		
Lipid transport and metabolism					
SF5M90T_1094	*acpP*	acyl carrier protein	**1.70**	**-3.04**	
SF5M90T_2272	*menE*	o-succinylbenzoate-CoA ligase	**1.60**	**-1.54**	**-1.80**
SF5M90T_2416	*ucpA*	putative oxidoreductase	**1.45**		**-0.56**
SF5M90T_339	*sbmA*	sensitivity to microcin B17, possibly envelope protein	**-1.64**		
Inorganic ion transport and metabolism					
SF5M90T_2903		hypothetical lipoprotein	**3.41**		
SF5M90T_929	*ycbO*	alkanesulfonate transporter substrate-binding subunit	**3.04**		
SF5M90T_2415	*cysP*	thiosulfate binding protein	**2.81**	**-1.51**	**-1.88**
SF5M90T_1636	*sodB*	superoxide dismutase	**2.52**	**2.13**	**1.67**
SF5M90T_1187		putative ATP-binding protein of ABC transporter	**2.14**		
SF5M90T_454	*copA*	copper exporting ATPase	**1.95**		
SF5M90T_1186		putative iron compound ABC transporter permease	**1.69**		
SF5M90T_1185		iron ABC transporter ATP-binding protein	**1.52**		
SF5M90T_4057	*yjcE*	predicted cation/proton antiporter	**1.49**		
SF5M90T_2675	*cysN*	ATP-sulfurylase (ATP:sulfate adenylyltransferase), subunit 1	**1.08**	**-2.08**	**-2.05**
SF5M90T_448	*ybaL*	putative transport protein	**-1.01**		**-0.50**
SF5M90T_2386	*mntH*	divalent metal cation transporter	**-1.93**	**1.61**	**1.68**
SF5M90T_330	*tauC*	taurine transport system permease protein	**-2.09**		
SF5M90T_3769	*shiF*	putative membrane transport protein	**-2.16**		
SF5M90T_3054	*ygjT*	putative transport protein	**-2.41**		
SF5M90T_1102	*fhuE*	outer membrane receptor for ferric iron uptake	**-2.46**		
SF5M90T_1483	*ydiE*	hemin uptake protein	**-2.93**	**-2.20**	**-1.55**
SF5M90T_1572	*mdtI*	spermidine export protein	**-3.61**		
Secondary metabolites biosynthesis, transport and catabolism					
SF5M90T_1184		putative SAM-dependent methyltransferase	**2.12**		
SF5M90T_331	*tauD*	taurine dioxygenase, 2-oxoglutarate-dependent	**-2.97**	**1.94**	
***Cellular processes and signalling***					
Cell cycle control, cell division, chromosome partitioning					
SF5M90T_1243	*yciB*	probable intracellular septation protein A	**0.93**		**-0.80**
Defense mechanisms					
SF5M90T_4215	*ampC*	beta-lactamase; penicillin resistance	**1.55**		**-1.69**
SF5M90T_3751	*emrD*	multidrug resistance protein D	**1.41**		
SF5M90T_4273		putative restriction modification enzyme R subunit	**1.41**	**-0.94**	**-0.99**
SF5M90T_3781	*shiA*	virulence factor	**1.30**		
SF5M90T_101	*ampD*	N-acetyl-anhydromuranmyl-L-alanine amidase	**1.18**		
SF5M90T_772	*ybhF*	putative ABC-type multidrug transport system component	**1.16**		**0.54**
SF5M90T_771	*ybhS*	putative ABC-type multidrug transport system component	**1.15**		
SF5M90T_770	*ybhR*	putative ABC-type multidrug transport system component	**0.90**		
SF5M90T_418	*mdlA*	ATP-binding component of a transport system	**-1.29**		
Signal transduction mechanisms					
SF5M90T_2126	*yehU*	putative 2-component sensor protein	**1.36**		
SF5M90T_3428	*uspA*	universal stress protein	**0.86**		
SF5M90T_2388	*yfeA*	predicted diguanylate cyclase	**-1.20**		**-0.81**
SF5M90T_4339	*creC*	sensory histidine kinase	**-1.63**	**1.43**	
Cell wall/membrane/envelope biogenesis					
SF5M90T_1923	*nmpC*	outer membrane porin protein	**2.04**	**-1.34**	**-1.13**
SF5M90T_1618	*slyB*	putative outer membrane protein	**1.82**	**-1.43**	**-0.92**
SF5M90T_952	*ompA*	outer membrane protein 3a	**1.59**		
SF5M90T_374	*tsx*	outer membrane protein	**1.53**		
SF5M90T_256	*gtrB*	bactoprenol glucosyl transferase	**1.36**	**-2.59**	
SF5M90T_2039	*rfbC*	dTDP-4-dehydrorhamnose 3,5-epimerase	**1.20**	**-2.64**	
SF5M90T_4332	*slt*	soluble lytic murein transglycosylase	**0.96**	**1.54**	**1.46**
SF5M90T_3951	*murI*	glutamate racemase	**0.93**		**-0.48**
SF5M90T_3821	*rfaD*	ADP-L-glycero-D-mannoheptose-6-epimerase	**0.82**		
SF5M90T_1241	*tonB*	transport protein	**-1.72**		
SF5M90T_3956	*murB*	UDP-N-acetylenolpyruvoylglucosamine reductase	**-2.23**		**-0.60**
Cell motility					
SF5M90T_1938	*fliQ*	flagellar biosynthetic protein	**3.55**		
Intracellular trafficking, secretion and vesicular transport					
SF5M90T_3964	*secE*	preprotein translocase	**0.87**	**-1.06**	**-0.76**
SF5M90T_3580	*tatC*	Sec-independent protein translocase	**0.84**		**-0.57**
SF5M90T_3501	*yiiO*	uncharacterized periplasmic protein	**-4.72**		**2.64**
Posttranslational modification, protein turnover, chaperones					
SF5M90T_4204	*mopB*	co-chaperonin GroES	**1.41**		
SF5M90T_3279	*slyD*	FKBP-type peptidyl-prolyl cis-trans isomerase	**1.31**		
SF5M90T_462	*ybbN*	putative thioredoxin-like protein	**0.90**		
SF5M90T_407	*clpP*	ATP-dependent proteolytic subunit of clpA-clpP serine protease	**0.84**		
SF5M90T_3738	*ibpA*	heat shock protein	**-1.17**		
SF5M90T_2074	*yegD*	putative heat shock protein	**-2.80**		**-1.11**
***Information storage and processing***					
Translation, ribosomal structure and biogenesis					
SF5M90T_2801	*yfiA*	translation inhibitor protein RaiA	**2.70**		
SF5M90T_2392	*gltX*	glutamate tRNA synthetase, catalytic subunit	**1.65**		
SF5M90T_155	*frr*	ribosome releasing factor	**1.21**		
SF5M90T_650	*glnS*	glutamine tRNA synthetase	**1.08**		
SF5M90T_3893	*glyQ*	glycine tRNA synthetase, alpha subunit	**1.06**		**-0.50**
SF5M90T_4220	*yjeA*	putative lysyl-tRNA synthetase	**0.95**	**-1.75**	**-1.72**
SF5M90T_3894	*glyS*	glycine tRNA synthetase, beta subunit	**0.81**		
Transcription					
SF5M90T_3025	*ygiP*	putative transcriptional regulator/nucleoid-associated protein	**3.04**	**-4.57**	**-2.45**
SF5M90T_2417	*murR*	HTH-type transcriptional regulator	**2.35**		
SF5M90T_3510	*rhaR*	positive regulator for *rhaRS* operon	**2.33**		
SF5M90T_1595	*malI*	repressor of *malX* and *Y* genes	**1.98**		
SF5M90T_2125	*yehT*	putative two-component response regulator	**1.80**		
SF5M90T_1373	*cspC*	cold shock protein	**1.59**	**-1.38**	
SF5M90T_3349	*malT*	positive regulator of *mal* regulon	**1.58**		**0.58**
SF5M90T_3335	*ompR*	osmolarity response regulator	**1.42**		
SF5M90T_3453	*yiaG*	putative transcriptional regulator	**1.38**		**3.65**
SF5M90T_2089	*gatR*	galactitol utilization operon repressor	**1.33**		
SF5M90T_71	*cra*	transcriptional repressor of *fru* operon and others	**1.16**		
SF5M90T_4197	*yjdC*	putative transcriptional regulator	**1.15**		**1.37**
SF5M90T_1370		putative regulator	**1.09**		
SF5M90T_3578	*rfaH*	transcriptional activator	**-1.57**		**-0.96**
SF5M90T_4242	*yjeB*	HTH-type transcriptional repressor	**-1.95**		
SF5M90T_984	*cspH*	cold shock-like protein	**-3.35**		
Replication, recombination and repair					
SF5M90T_2925	*endA*	DNA-specific endonuclease I	**1.42**	**-3.26**	**-2.74**
SF5M90T_3034	*ygjF*	G/U mismatch-specific DNA glycosylase	**1.23**		**1.10**
SF5M90T_410	*hupB*	DNA-binding protein HU-beta	**1.08**		
SF5M90T_775	*rhlE*	putative ATP-dependent RNA helicase	**-1.16**		
SF5M90T_3117	*deaD*	inducible ATP-independent RNA helicase	**-1.20**		
SF5M90T_1769	*dbpA*	ATP-dependent RNA helicase	**-1.86**		
***Poorly characterized***					
General function prediction only					
SF5M90T_2762	*stpA*	DNA-binding protein	**3.51**	**-3.00**	**-2.60**
SF5M90T_275		putative crossover junction endodeoxyribonuclease	**2.84**		
SF5M90T_2418	*muQ*	N-acetylmuramic acid 6-phosphate etherase	**2.77**		**-0.93**
SF5M90T_1724		putative acetyltransferase	**2.06**	**-2.41**	
SF5M90T_2435		putative amino acid antiporter	**2.03**	**1.90**	**1.58**
SF5M90T_2301	*yfbT*	putative phosphatase	**2.00**		
SF5M90T_773	*ybhG*	putative membrane protein	**1.78**	**1.02**	
SF5M90T_1227	*hns*	DNA-binding protein	**1.69**	**-1.24**	
SF5M90T_2275	*yfbB*	putative enzyme	**1.62**	**-1.90**	**-1.85**
SF5M90T_3225	*yrdA*	putative transferase	**1.51**		
SF5M90T_4236	*hfq*	RNA-binding protein	**1.45**	**-2.10**	
SF5M90T_3315	*gph*	phosphoglycolate phosphatase	**1.25**		
SF5M90T_2192	*yeiR*	putative GTPases	**1.03**		
SF5M90T_1919	*yedE*	putative transport system permease protein	**1.03**	**-1.83**	**-1.96**
SF5M90T_3295	*yhfC*	putative transport	**0.97**	**-1.30**	**-1.25**
SF5M90T_2205	*yejK*	nucleoid-associated protein	**0.95**		
SF5M90T_2066	*yegH*	putative transport protein	**0.84**		
SF5M90T_3344	*yhgH*	putative gluconate periplasmic binding protein	**0.81**		
SF5M90T_3102	*yraM*	putative glycosylase	**0.73**		
SF5M90T_794	*ybiP*	putative enzyme	**-1.14**		
SF5M90T_2207	*yejM*	putative sulfatase	**-1.36**		
SF5M90T_3139	*yhbE*	putative permeases of drug/metabolite transporter superfamily	**-1.36**		**-0.90**
SF5M90T_966	*yccA*	putative carrier/transport protein	**-1.38**		**-1.18**
SF5M90T_2742	*yqaB*	putative phosphatase	**-1.51**		
SF5M90T_3882	*bax*	putative ATP-binding protein	**-1.70**		**-0.77**
SF5M90T_3370	*yhhX*	putative regulator	**-1.97**		**1.30**
SF5M90T_3621	*aslB*	putative arylsulfatase regulator	**-2.73**		
SF5M90T_2516		putative enzyme	**-3.91**		
Function unknown					
SFxv_3833		conserved hypothetical protein	**3.59**	**-4.23**	
SF5M90T_2431		conserved hypothetical protein	**2.89**		
SF5M90T_2432		conserved hypothetical protein	**2.69**		
SF5M90T_11		uncharacterized protein	**2.57**		
SF5M90T_1402	*yeaD*	conserved hypothetical protein	**2.45**		
SF5M90T_828	*ybjO*	conserved hypothetical protein	**2.23**		**-2.52**
SF5M90T_1941	*dsrB*	conserved hypothetical protein	**2.03**	**-2.43**	
SF5M90T_2302	*yfbU*	conserved hypothetical protein	**1.87**		
SF5M90T_451	*ybaK*	conserved hypothetical protein	**1.86**	**-1.21**	**-1.10**
SSJG_00311		conserved hypothetical protein	**1.75**		**-1.60**
SF5M90T_5	*yaaA*	conserved hypothetical protein	**1.54**		
SF5M90T_1387		conserved hypothetical protein	**1.51**		
SF5M90T_3911	*yiiU*	conserved hypothetical protein	**1.45**	**-2.49**	
SF5M90T_957		conserved hypothetical protein	**1.37**		
SF5M90T_4146	*yjgD*	conserved hypothetical protein	**1.28**	**-2.30**	
SF5M90T_2622		conserved hypothetical protein	**1.24**	**-1.46**	
SF5M90T_3155	*yhbN*	conserved hypothetical protein	**0.81**		
SF5M90T_479	*ybbF*	conserved hypothetical protein	**-1.19**		
SF5M90T_2195	*rtn*	conserved hypothetical protein	**-1.50**		
SF5M90T_438	*ybaN*	conserved hypothetical protein	**-1.73**		
SF5M90T_1853		conserved hypothetical protein	**-2.03**	**-2.58**	
SF5M90T_1647		conserved hypothetical protein	**-2.11**		
SF5M90T_983	*ymcD*	conserved hypothetical protein	**-2.34**		
SF5M90T_4094	*yjbA*	P-starvation inducible protein PsiE	**-2.51**	**1.69**	
SF5M90T_1110	*ycfJ*	conserved hypothetical protein	**-2.52**		**0.99**
SF2861		hypothetical protein remnant	**-2.64**		
SF5M90T_2146	*yohO*	membrane protein	**-2.96**		
SF5M90T_1952		putative outer membrane pore protein	**-2.98**		
SF5M90T_4307		putative inner membrane protein	**-3.40**		
SF1231		conserved hypothetical protein	**-3.71**		**-1.60**
SF5M90T_427	*ybaA*	conserved hypothetical protein	**-3.88**		
Phage related					
S1668	*relF*	prophage maintenance protein	**1.75**		
SF5M90T_1793		putative phage integrase protein	**1.45**	**-1.60**	
SF5M90T_1056		hypothetical bacteriophage protein	**1.14**		
SF5M90T_740		putative bacteriophage protein	**-1.93**		

^a^Genomes used as reference are: *S. flexneri* 5a str. M90T, *S. flexneri* 2a str. 301, *S. flexneri* 2002017, *Shigella* sp. D9 and *S. flexneri* 2457 T with GenBank accession numbers AGNM00000000, NC_004337, NC_017328, NZ_GG657384 and NC_004741 respectively.

^b^Genes are classified in functional categories based on the database of Clusters of Orthologous Groups (COGs). http://www.ncbi.nlm.nih.gov/COG/. Inside each subgroup, genes are arranged in descending order in relation to Log2 of Fold Change values of WT no O_2_/WT O_2_ comparison.

^c^Log2 of Fold Change values of WT no O_2_/WT O_2_ and Δ*fnr* no O_2_/WT no O_2_ comparisons are presented. Only values considered differentially expressed are shown (*p* adjust <0.05).

The expression of other genes involved in transport displayed altered expression in anaerobiosis. For instance, *emrD,* coding for a drug transporter, *cysP*, involved in the binding and uptake of sulfate and thiosulfate, *yjcE,* coding for a Na^+^/H^+^ exchanger, *ybgH,* which encodes a peptide transporter and genes involved in nucleoside transport and catabolism (*tsx, nupC, nupG* and *udp*) are induced in anaerobiosis (Table 1, Figure 1A) [33–40].

We found several metabolic genes induced under anaerobic growth such as *cra*, coding for the catabolite repressor/activator protein, Cra, *tpiA*, encoding a key enzyme of the gluconeogenic and glycolytic pathways, *gapA*, involved in glycolysis, *yehU/yehT,* coding for a two component system involved in responses to carbon starvation, *malT*, the transcriptional activator of the genes responsible for uptake and metabolism of maltodextrins and *proA*, which encodes an enzyme in proline biosynthesis [41–47]. The expression of these genes was not FNR-dependent (Table 1, Figure 1A).

**Figure 2:**
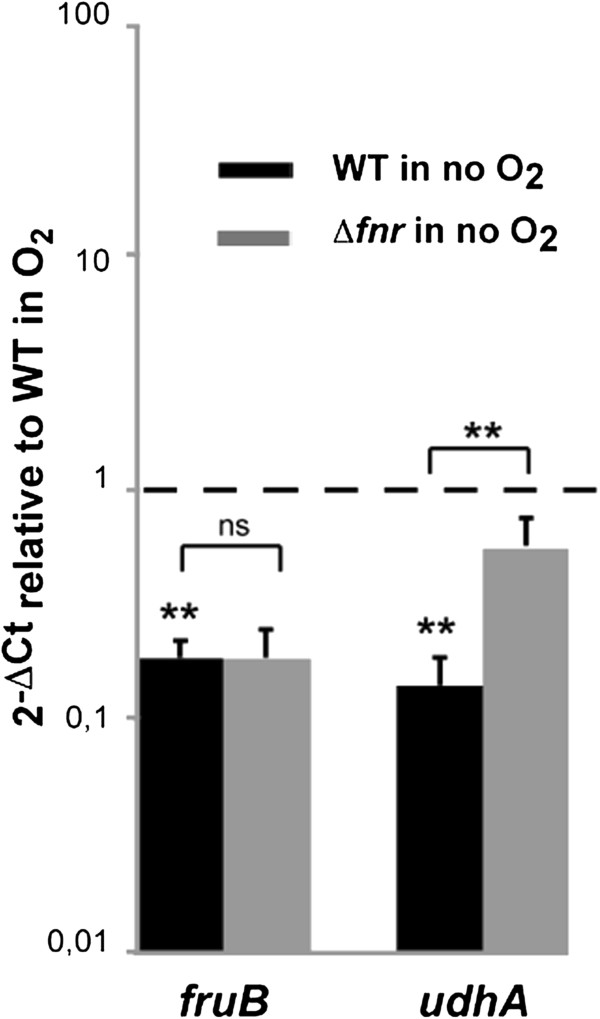
**qRT-PCR verification of** ***S. flexneri*** **chromosomal genes repressed under anaerobic growth conditions and the role of FNR in the repression.** Strand specific qRT-PCR analysis of mRNA levels of *S. flexneri* M90T chromosomal genes shown to be repressed in RNA-seq analysis. Data were calculated as the n-fold difference relative to *polA* (2^-Δ*Ct*^, where ΔCt represents the difference in threshold cycle between the target and control genes). Results are shown in relation to wild-type 2^-Δ*Ct*^ levels under aerobic conditions, here referred to as 1. Thus, values greater than 1 indicate increased transcription under anaerobiosis and lower than 1 indicates the opposite. Significant differences were detected when wild-type 2^-Δ*Ct*^ levels under aerobic and anaerobic conditions or wild-type *vs.* Δ*fnr* 2^-Δ*Ct*^ levels under anaerobiosis were compared. *P* <0.01, **; n = 4; Mann–Whitney test. Error bars show Standard Deviation (SD).

In addition to metabolism, we observed anaerobic up-regulation of: genes involved in stress response such as *cspC*; genes coding for outer membrane proteins (OMPs) such as NmpC, OmpA and SlyB; genes with global regulatory functions such as *yjgD* that codes for RraB, which interacts with the endonuclease RNase E; *yfiA,* encoding a ribosome-associated protein that inhibits protein translation; and *yejK*, *hns* and its paralogue *stpA* coding for nucleoid-associated proteins responsible for chromosomal DNA compaction and global gene regulation [48–56]. Interestingly, anaerobic induction of *cspC*, *nmpC, slyB, yjgD, hns* and *stpA* was dependent, at least in part, on FNR (Table 1, Figure 1B). Anaerobiosis can also down-regulate transcription. This is the case for *fruBKA*, encoding the fructose PTS [29] (Table 1, Figure 2).

The analysis of genes known to be influenced by anaerobiosis revealed further functions of FNR. This is the case for *ygiP*, encoding a nucleoid-associated protein induced under anaerobic growth conditions, which we found is FNR-dependent [57]. Furthermore, we observed that *menDBCE*, genes required for the biosynthesis of quinones with essential roles in anaerobic electron transport systems, are affected by the presence of FNR in contrast to *E. coli* (Table 1, Figure 1A and B) [58–60].

Our study revealed extended regulatory roles for FNR, such as in the biosynthesis of L-cysteine. Previous work has demonstrated that *cysK*, which encodes an enzyme in L-cysteine biosynthesis, is subject to FNR regulation and identified an FNR-like domain in *cysJ,* which encodes a component of the sulfite reductase [16, 61]. Here, we found that loss of FNR affects the entire L-cysteine biosynthetic pathway including genes involved in the uptake and transport of sulfate (*i.e. cysPUWAM*), sulfate activation (*cysDN*), reduction to sulfide (*cysJIH*) and transformation into L-cysteine (*cysK*) (Table 1, Figure 1A, see Additional file 1: Table S2) [62–64].

### Reprogramming of T3SS related genes under anaerobic conditions

**Figure 3:**
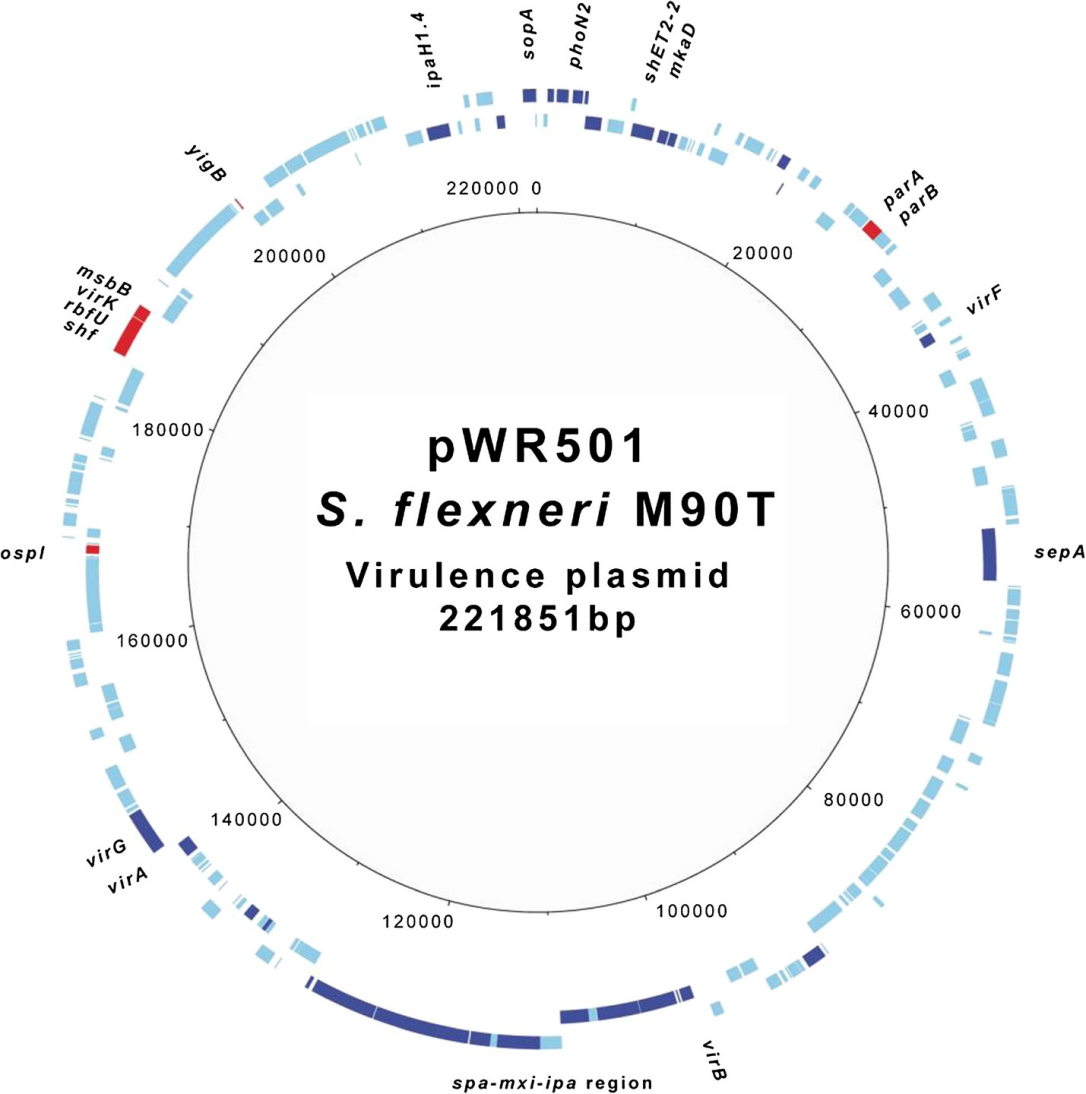
**Circular map of genes differentially expressed in the virulence plasmid under anaerobiosis.** Outer ring shows ORFs and their orientations. Genes differentially repressed and induced in the wild type M90T strain under anaerobiosis in relation to aerobic conditions were marked in deep blue and red respectively. Scale is in base pairs. The figure was generated with DNAPlotter.

**Figure 4:**
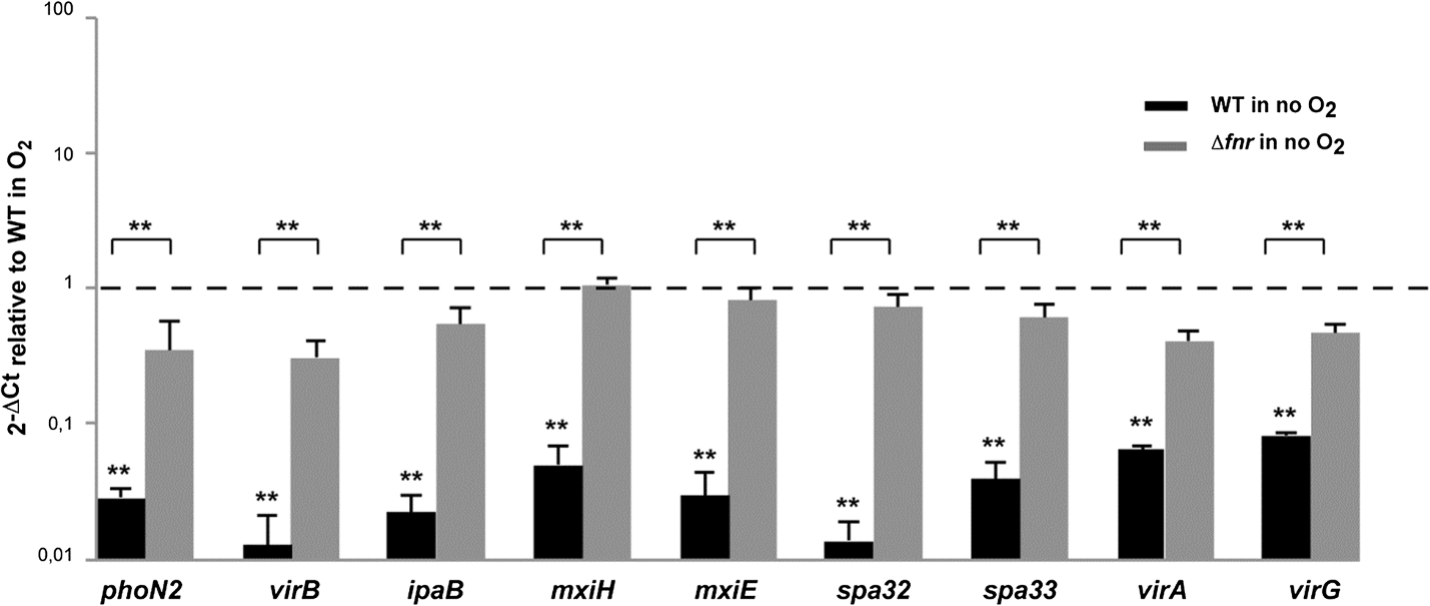
**qRT-PCR verification of** ***S. flexneri*** **virulence plasmid genes repressed under anaerobic growth conditions and the role of FNR in the repression.** Strand specific qRT-PCR analysis of *S. flexneri* M90T virulence plasmid genes mRNA levels shown to be repressed in RNA-seq analysis. Data were calculated as the n-fold difference relative to *polA* (2^-Δ*Ct*^, where ΔCt represents the difference in threshold cycle between the target and control genes). Results are shown in relation to the wild-type 2^-Δ*Ct*^ levels under aerobic conditions (referred to as 1). Values greater than 1 indicate increased transcription under anaerobiosis, while lower than 1 indicate the opposite. Significant differences were detected with the wild-type strain 2^-Δ*Ct*^ levels under aerobic and anaerobic conditions, or wild-type *vs.* Δ*fnr* 2^-Δ*Ct*^ levels under anaerobiosis were compared. P <0.01, **; n = 4; Mann–Whitney test. Error bars show Standard Deviation (SD).

**Figure 5:**
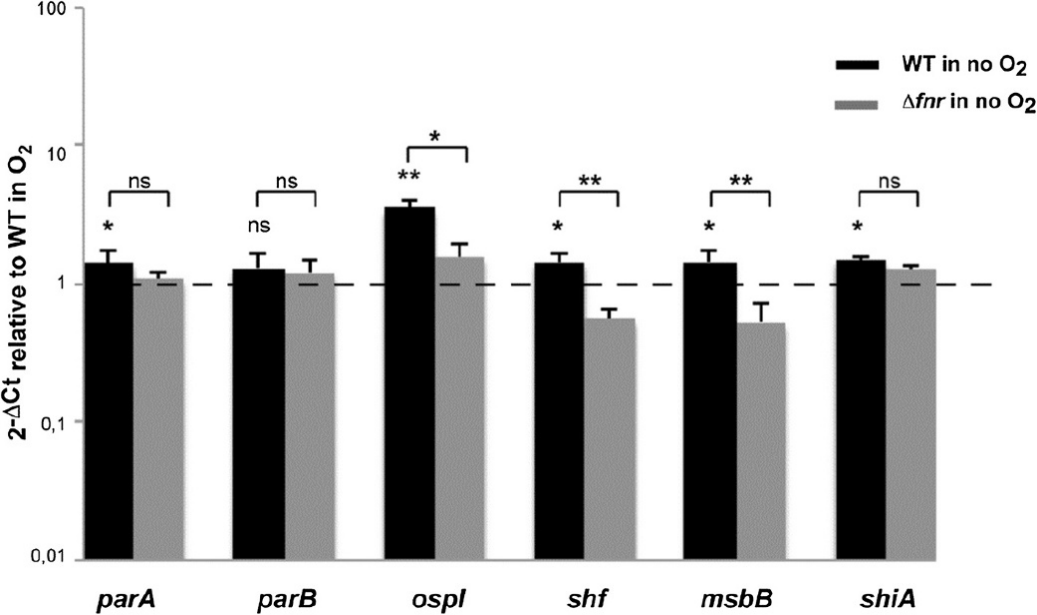
**qRT-PCR verification of** ***S. flexneri*** **virulence plasmid genes induced under anaerobic growth conditions and the role of FNR in the induction.** Strand specific qRT-PCR analysis of *S. flexneri* M90T virulence genes mRNA levels shown to be induced in RNA-seq analysis. Data were calculated as the n-fold difference relative to *polA* (2^-Δ*Ct*^, where ΔCt represents the difference in threshold cycle between the target and control genes). Results are shown in relation to the wild-type 2^-Δ*Ct*^ levels under aerobic conditions (referred to as 1). Values greater than 1 indicate increased transcription under anaerobiosis, while lower than 1 indicate the opposite. Significant differences were detected with the wild-type strain 2^-Δ*Ct*^ levels under aerobic and anaerobic conditions, or wild-type *vs.* Δ*fnr* 2^-Δ*Ct*^ levels under anaerobiosis were compared. *P* < 0.05, *; P <0.01, **; n = 4; Mann–Whitney test. Error bars show standard deviation (SD).

Analysis of genes involved in *Shigella* virulence revealed that multiple genes on the *Shigella* virulence plasmid, including *ipa-mxi-spa* genes, were repressed under anaerobic growth in an FNR-dependent manner (Table 2). In contrast, only seven genes on the plasmid (*yigB, ospI*, *shf, rfbU, virK, msbB* and *parA*) were up-regulated in the absence of oxygen; all of these are regulated by FNR except *parA* and *yigB* (Table 2). Figure 3 shows effect of oxygen on expression of genes on the virulence plasmid genes. These findings were confirmed by strand specific qRT-PCR for several genes (Figures 4 and 5). Since excess ParA levels compared with ParB can affect plasmid partitioning, we also examined the transcription profile of *parB*[65]. Similar to *parA*, mRNA levels of *parB* are elevated during anaerobic growth (Figure 5). Consistent with this finding, there was no significant difference in loss of the virulence plasmid from bacteria grown in aerobic and anaerobic conditions (not shown).

**Table 2:** **Virulence plasmid genes differentially expressed in response to anaerobic conditions**

ORF ID ^ab^	Gene	Description	RNA-seq ^c^ log2FC	RNA-seq ^c^ log2FC	FRT-seq ^c^ log2FC
			WT no O _2_ /O _2_	Δ ***fnr*** /WT no O _2_	Δ ***fnr*** /WT no O _2_
pWR501_0265	*yigB*	hypothetical protein	**2.56**		
pWR501_0225	*ospI*	T3SS effector	**2.00**	**-2.21**	
pWR501_0250	*shf*	peptidoglycan deacetylase	**1.24**	**-3.42**	**-1.30**
pWR501_0251	*rfbU*	glycosiltransferase	**1.21**	**-1.65**	**-1.16**
pWR501_0252	*virK*	virulence protein	**1.15**	**-2.16**	**-1.03**
pWR501_0039	*parA*	plasmid segregation protein	**1.13**		
pWR501_0253	*msbB*	acyltransferase	**1.07**	**-3.03**	**-1.08**
pWR501_0074	*sepA*	secreted protease	**-1.12**	**1.34**	**1.52**
pWR501_0177		hypothetical protein	**-1.55**		**1.04**
pWR501_0283	*ipaH1.4*	T3SS effector	**-1.56**		
pWR501_0175		hypothetical protein	**-1.58**		**2.29**
pWR501_0176		hypothetical protein	**-1.76**		**2.02**
pWR501_0015		hypothetical protein	**-2.00**	**1.52**	**1.05**
pWR501_0002		putative resolvase	**-2.04**	**1.97**	
pWR501_0007		hypothetical protein	**-2.25**	**1.63**	
pWR501_0014		hypothetical protein	**-2.25**	**1.60**	**1.27**
pWR501_0192	*virG*	invasion protein	**-2.25**		**2.36**
pWR501_0144	*ipgF*	unknown function	**-2.38**		**3.20**
pWR501_0051	*virF*	transcriptional activator of virulence	**-2.47**		
pWR501_0006		hypothetical protein	**-2.54**		
pWR501_0143	*ipgE*	chaperon	**-2.55**		**3.39**
pWR501_0146	*mxiH*	T3SS component	**-2.56**	**1.55**	**4.10**
pWR501_0122		hypothetical protein	**-2.58**	**1.53**	**1.95**
pWR501_0121		hypothetical protein	**-2.65**		
pWR501_0191	*virA*	T3SS effector	**-2.66**		**2.42**
pWR501_0147	*mxiI*	T3SS component	**-2.80**	**2.00**	**4.01**
pWR501_0013	*mkaD*	mouse killing factor	**-2.81**	**1.97**	**3.33**
pWR501_0145	*mxiG*	T3SS component	**-3.02**		**3.45**
pWR501_0148	*mxiJ*	T3SS component	**-3.06**	**2.17**	**4.33**
pWR501_0031		hypothetical protein	**-3.14**		
pWR501_0005		hypothetical protein	**-3.14**		**2.49**
pWR501_0292	*sopA*	VirG-specific protease	**-3.25**		**2.49**
pWR501_0291		hypothetical protein	**-3.31**		
pWR501_0138	*ipgB*	invasion protein	**-3.34**		**3.66**
pWR501_0157	*spa15*	chaperon	**-3.34**	**3.52**	**4.43**
pWR501_0012	*shET2-2*	enterotoxin	**-3.38**		**3.41**
pWR501_0156	*mxiA*	T3SS component	**-3.44**		**4.67**
pWR501_0160	*spa32*	invasion protein	**-3.45**	**1.34**	**4.63**
pWR501_0141		hypothetical protein	**-3.50**		**3.71**
pWR501_0004	*phoN2*	apyrase	**-3.52**	**2.38**	**3.25**
pWR501_0150	*mxiL*	hypothetical protein	**-3.59**	**2.07**	**4.64**
pWR501_0132	*acp*	hypothetical protein	**-3.62**		**5.19**
pWR501_0166	*spa-orf10*	hypothetical protein	**-3.64**	**1.97**	**4.52**
pWR501_0158	*spa47*	T3SS component	**-3.65**	**3.19**	**4.69**
pWR501_0140	*icsB*	T3SS effector	**-3.65**	**2.16**	**4.43**
pWR501_0162	*spa24*	T3SS component	**-3.70**	**2.28**	**3.71**
pWR501_0159	*spa13*	T3SS component	**-3.75**		**5.13**
pWR501_0161	*spa33*	T3SS component	**-3.75**	**1.99**	**4.03**
pWR501_0151	*mxiM*	T3SS component	**-3.81**	**2.40**	**4.25**
pWR501_0155	*mxiC*	T3SS component	**-3.86**	**2.43**	**4.95**
pWR501_0290		hypothetical protein	**-3.86**		**2.00**
pWR501_0030		putative enterotoxin fragment	**-3.90**	**2.20**	**4.54**
pWR501_0163	*spa9*	T3SS component	**-3.92**	**1.93**	**3.56**
pWR501_0137	*ipgC*	chaperon	**-3.93**	**1.86**	**4.04**
pWR501_0135	*ipaC*	T3SS effector	**-3.93**	**2.65**	**5.01**
pWR501_0165	*spa40*	T3SS component	**-3.94**		**4.02**
pWR501_0139	*ipgA*	chaperon	**-3.95**	**2.45**	**4.25**
pWR501_0153	*mxiD*	T3SS component	**-3.98**	**2.33**	**4.81**
pWR501_0152	*mxiE*	transcriptional activator	**-4.06**	**2.51**	**4.48**
pWR501_0154	*mxiD*	T3SS component	**-4.08**	**2.66**	**4.54**
pWR501_0134	*ipaD*	T3SS effector	**-4.16**	**2.99**	**4.92**
pWR501_0167	*spa-orf11*	hypothetical protein	**-4.19**	**2.85**	**4.07**
pWR501_0136	*ipaB*	T3SS effector	**-4.24**	**2.97**	**4.59**
pWR501_0133	*ipaA*	T3SS effector	**-4.24**	**3.09**	**4.98**
pWR501_0003		hypothetical protein	**-4.57**		**3.29**
pWR501_0164	*spa29*	T3SS component	**-5.06**	**2.36**	**3.32**
pWR501_0131	*virB*	transcriptional activator	**-5.17**	**2.40**	**4.26**

^a^ *S. flexneri* 5a str. M90T pWR501 virulence plasmid sequence was used as reference GenBank accession numbers AF348706.

^b^Genes are arranged in descending order in relation to Log2 of Fold Change values of WT no O_2_/WT O_2_ comparison.

^c^Log2 of Fold Change values of WT no O_2_/WT O_2_ and Δ*fnr* no O_2_/WT no O_2_ comparisons are presented. Only values considered differentially expressed are shown (*p* adjust <0.05).

The *Shigella* pathogenicity island SHI-1 is not present in *S. flexneri* M90T. Therefore, we examined the transcriptional profile of the SHI-2 pathogenicity island that includes the aerobactin, iron-uptake system [66]. As previously reported, we found that genes encoding the aerobactin system (*iucABCD* and *iutA*) were down-regulated under anaerobic conditions, as was *shiF*, a gene which is also involved in iron acquisition [6, 67]. In contrast, *shiA*, a SHI-2 gene involved in attenuating host inflammatory responses, was over-expressed under anaerobic conditions when compared to aerobic conditions [68]. Of note, no SHI-2 gene is subject to FNR regulation (Table 1, Figure 5, see Additional file 1: Table S1).

### csrB and csrC sRNAs are induced in the absence of oxygen in *S. flexneri*M90T

**Figure 6:**
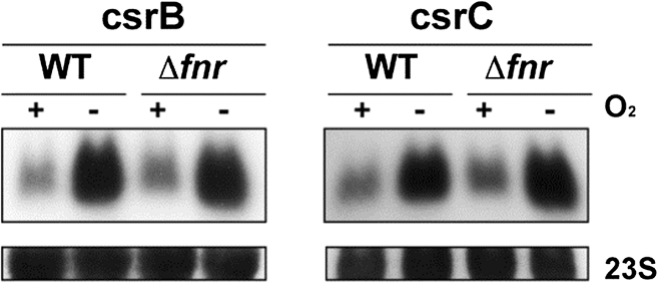
**Verification of sRNAs results by Northern Blot.** Northern blot analysis of csrB and crsC sRNAs expression under aerobic/anaerobic conditions. 10 μg of total RNA obtained from *S. flexneri* M90T wild‒type strain and its isogenic Δ*fnr* mutant grown under aerobic and anaerobic conditions until OD_600_ = 0.2 were separated in 1,25% MOPS‒agarose gels, transferred to membranes and detected using probes specific for the sense strand.

Little is known about the small RNAs (sRNAs) in *Shigella* or their expression under anaerobic conditions. We analysed the sRNAs already described in *Shigella* as well as potential sRNAs homologues to those described in *S. enterica* serovar Typhimurium and found that anaerobic growth conditions induce the expression of csrB and csrC in an FNR-independent manner (Table 3, Figure 6) [69–71].

**Table 3:** **sRNAs differentially expressed in response to anaerobic conditions**

sRNA ^a^	Adjacent genes	Description/class	Length (nt)	RNA-seq ^b^ log2FC	RNA-seq ^b^ log2FC
				WT no O _2_ /O _2_	Δ ***fnr*** /WT no O _2_
csrB	*syd/*SF5M90T_2595	protein-binding sRNA	**360**	**4.97**	
csrC	*yihi/yihA*	protein-binding sRNA	**245**	**3.38**	

^a^sRNAs are arranged in descending order in relation to Log2 of Fold Change values of WT no O_2_/WT O_2_ comparison.

^b^Log2 of Fold Change values of WT no O_2_/WT O_2_ and Δ*fnr* no O_2_/WT no O_2_ comparisons are presented. Only values considered differentially expressed are shown (*p* adjust <0.05).

## Discussion

*In vitro* studies have several limitations in relation to *in vivo* studies; e.g., they cannot mimic the amount and type of carbon sources available for bacteria and lack the signals derived from the interaction with intestinal epithelium, human immune system or other bacteria present in the gut. However, if conducted accurately can provide valuable information.

In the current study we have, for the first time, employed RNA-sequencing to identify oxygen regulated genes in an enteric pathogen. Our findings confirm previous results, but as this method is more sensitive than array based approaches, we identified an extended repertoire of genes modulated by oxygen in an FNR-dependent or -independent manner. For instance, little is known about the role of Cra, a transcriptional regulator of carbon flux (that represses glycolysis and activates gluconeogenesis) here shown to be induced under anaerobic conditions [41]. Interestingly, mutation of *cra* increases both epithelial cell attachment and invasion by *Shigella* in aerobic conditions [72]. However, Cra has an entirely distinct role in the virulence of enterohemorrhagic *E. coli* (EHEC), a close relative of *Shigella*, when investigated under conditions mimicking the anaerobic environment of the intestinal tract. Under these circumstances, loss of Cra reduces attachment of bacteria to enterocytes [73]. Additionally, *Salmonella cra* mutants are avirulent when administered orally, indicating that Cra may have key roles in enteric pathogens in anaerobic conditions [74]*.*

While there is an increasing recognition that carbon metabolism affects microbial virulence, it is still not clear whether distinct carbon energy sources are important or preferable for different members of the *Enterobacteriaceae*[72, 75–80]. For example, our results show that the expression pattern under anaerobic conditions of *ptsG*, *manXYC and fruBKA* involved in the transport of sugars is opposite in *Shigella* to that observed in *E. coli*[18, 20]. This could be simply due to the different growth medium used in the experiments or to distinct metabolic strategies between *Shigella* and other *Enterobacteriaceae*. In favour of the latter and its relationship with virulence it has been shown that mutation of *ptsG* induces the adherence and invasive capacity of enteroinvasive *E. coli* (EIEC) strains but not in *Salmonella*[81]. Further differences between *Shigella* and other *Enterobacteriaceae* include *adiY,* an AraC-like regulator, which activates expression of *adiA* and *adiC,* encoding the arginine-dependent acid resistance system (AR3). In *Salmonella adiY* expression is elevated under aerobic conditions, whereas in *Shigella* and in *E. coli,* increased expression of *adiY* occurs in anaerobiosis [20, 82]. These differences could be due to the strikingly different acid survival strategies that these bacteria seem to develop in spite of being close relatives [83]. Deletion of *cad* locus, a typical pathoadaptive mutation in *Shigella* spp., also induces the AR3 system suggesting that this system contributes to the survival of *Shigella* in its particular niche in the intestinal tract [84, 85].

Interestingly, we observed an FNR-dependent elevated expression under anaerobiosis of *hns* and overall of *stpA* and *ygiP* that encode nucleoid-associated proteins responsible for DNA compaction and global gene regulation, indicating that lack of oxygen profoundly modifies DNA topology in *Shigella*. Recently, it has been shown that FNR function is strongly inhibited by this kind of nucleoid-associated proteins, which block FNR access to many binding sites [20]. Our findings suggest that FNR is involved in this inhibition, probably indirectly, due to the absence of putative FNR binding-boxes in the promoter region of these genes [20].

To distinguish between direct and indirect effects of FNR, *in vivo* approaches based in chromatin immunoprecipitation followed by micro-array hybridization (ChIP-chip) or high-throughput sequencing (ChIP-seq) have been performed in *E. coli*[20, 86]. Correlation of FNR ChIP-seq peaks with transcriptomic data showed that less than half of the FNR-regulated operons could be attributed to direct FNR binding. Of note, FNR occupancy does not always correlate with the presence of a consensus FNR binding site or a change in expression [20, 86]. A total of 19 of *E. coli* ChIP-seq peaks are located in promoter regions of genes identified in Table 1 (*i.e. ptsG, pfkA, gapA, yegT, ptsH, tpiA, lysC, menD, ribB, uspA, slyB, ompA, tonB, yjeA, cspH, deaD, dbpA, yccA* and *yhhX*); only one of these, *dbpA,* has a canonical FNR binding sequence in its promoter region. Consistent with previous findings, only six of these 19 genes (*lysC, menD, slyB, yjeA, yccA* and *yhhX*) were influenced by FNR in our transcriptomic analysis. This result suggests that many FNR effects in Table 1 are likely to be indirect. However, we cannot rule out differences in regulation between *E. coli* and *Shigella* that could affect FNR function. Of note, this is the first time that *menD, slyB, yjeA* and *yhhX* have been identified as FNR regulated by transcriptome analysis, corroborating previous ChIP findings performed in *E. coli.*

sRNAs are widespread in bacteria and play critical roles in regulating physiological processes [87]. In *Shigella*, putative sRNAs have been identified by bioinformatics [69, 70]. However, the expression of these sRNAs has not been confirmed in all cases and little is known about their function or the physiological conditions that induce their expression. Here, we found that anaerobic growth induces expression of two sRNAs, csrB and csrC, independently of FNR*.* In *E. coli* csrB and csrC regulate the activity of CsrA, the carbon storage regulator although their function in *Shigella* has not been characterised so far [88, 89].

For genes directly involved in host:pathogen interactions, we found that oxygen influences the expression of almost all genes in the *mxi-spa* operon. These T3SS-related genes were down-regulated in the absence of oxygen in an FNR-dependent manner. This is likely to be mediated by VirB as this transcription factor controls many genes in this operon, is influenced by H-NS dependent DNA supercoiling and our findings demonstrate that *virB* gene is repressed in anaerobiosis [90]. The effect of oxygen on the *Shigella* T3SS is opposite to *Salmonella* in which FNR induces expression of invasion genes, and probably reflects the different sites occupied in the host by these two related intestinal pathogens [19]. The results further emphasise that the *Shigella* T3SS is inactive in anaerobic environments as we previously reported [7].

Inflammation at the site of invasive infection is a hallmark of intestinal shigellosis [91, 92]. Of note, expression of *shiA* is induced under anaerobiosis. This gene in the SHI-2 pathogenicity island encodes a factor that attenuates the intestinal inflammatory response in shigellosis by decreasing the recruitment of polymorphonuclear leukocytes and T-cells [68, 93]. Similarly OspI is the only T3SS-effector protein that was overexpressed in anaerobiosis; it also serves to dampen inflammatory responses by deaminating a glutamine in host ubiquitin-conjugating enzyme (UBC13) [94]. Thus, expression of both ShiA and OspI under low oxygen tension might dampen the extent of inflammatory responses to *Shigella* while it is in the anoxic environment of the intestinal lumen, impairing immune responses. Only one operon on the virulence plasmid, *shf-rfbU-virK-msbB*, was induced under anaerobiosis in an FNR-dependent manner. Interestingly, all these genes are implicated in modification of *Shigella* lipopolysaccharide (LPS), an important pro-inflammatory mediator [95–99].

The transcription of several genes encoding OMPs was induced under anaerobic growth. Both OmpA and OmpC have been implicated in *Shigella* virulence, while our results suggest that Tsx, Slp, NmpC, SlyB and YciD (OmpW) could also contribute to pathogenesis and be considered as potential vaccine targets [100, 101]. Indeed, *Salmonella* OmpW, Tsx and NmpC have already been demonstrated to be immunogenic [102, 103]. In addition to OMPs, transcription of *gapA,* which encodes glyceraldehyde-3-phosphate dehydrogenase, was induced under anaerobic conditions. Interestingly, this enzyme is exported by EHEC and enteropathogenic *E. coli* (EPEC) strains but not by non-pathogenic strains. Due to its ability to interact with plasminogen, fibrinogen and intestinal epithelial cells, it has been suggested that GapA might contribute *in vivo* to the interaction of EHEC and EPEC with the gut epithelium [104].

## Conclusions

**Figure 7:**
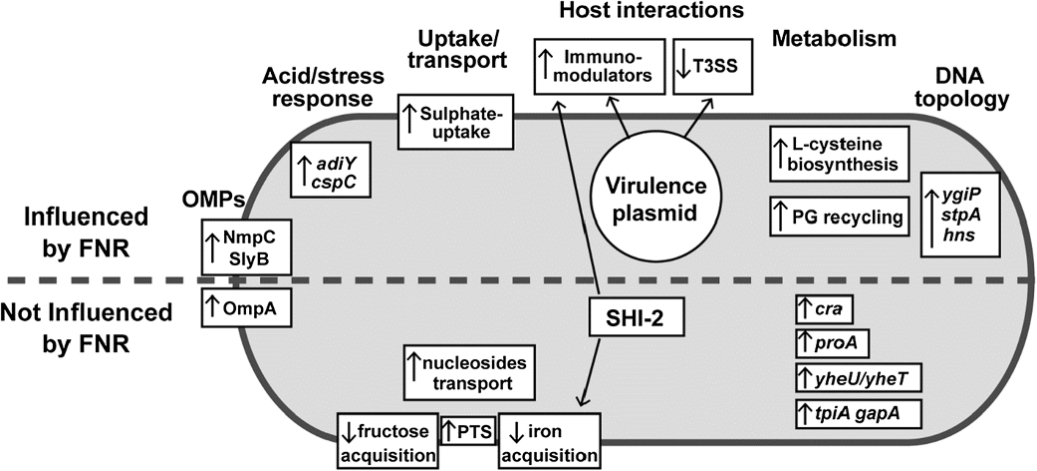
**Summary of novel genes influenced by the absence of oxygen in** ***Shigella*** **identified by RNA-seq.**

Overall, our RNA-seq based analysis revealed that in the anaerobic lumen of the intestine *Shigella* is predicted to prompt both survival and anti-host immune-modulatory activities of the bacterium. This occurs through a reprogramming of bacterial metabolism including altered transcription of genes encoding transport systems and metabolic pathways (Figure 7), likely reflecting the carbon energy sources available in the intestine. Modulation of LPS, along with ShiA and OspI may enable *Shigella* to subvert inflammatory responses prior to mucosal invasion. Our results highlight the central role of oxygen and FNR in these processes and how it governs bacterial interactions and entry into host cells [7, 68].

## Methods

### Bacterial strains and culture conditions

Bacterial strains and plasmids used in this study are shown in Additional file 1: Table S4. *E. coli* strains were grown in Luria-Bertani (LB; Invitrogen) broth or on LB agar plates while *S. flexneri* was propagated either in LB broth, tryptic soy broth (TCS; Sigma) or on TCS plates with Congo red (0.01%, Sigma). Experiments under anaerobiosis were performed in an anaerobic workstation (Whitley A35). When required, antibiotics were added at the following concentrations: chloramphenicol 20 μg/ml, ampicillin 100 μg/ml.

### Deletion of *fnr*gene and complementation experiments

The *fnr* deletion mutant was generated by allelic exchange using pKO3blue plasmid as previously described [105]. Oligonucleotide primers used in this study are listed in Additional file 1: Table S5. Complementation of Δ*fnr* mutant was performed with pBM2, a derivative of pBBR1MCS-4 plasmid that carries a copy of *fnr* gene under the control of its native promoter. The plasmid pBBR1MCS-4 was used as a control (See Additional file 1: Table S4). The absence of FNR in the Δ*fnr* mutant and its presence in the complemented strain was confirmed by western blot using polyclonal antibodies against FNR as previously described [7] (See Additional file 1: Figure S1).

### DNA and RNA extraction methods

*S. flexneri* M90T genomic DNA for sequencing was isolated as previously described [106]. For RNA extraction bacteria were grown in LB medium with and without oxygen. A 5 ml pre-inoculum was grown over night aerobically or anaerobically with shaking conditions. The pre-inoculums were diluted proportionally to their OD_600nm_ to standardize the input of bacteria to a starting OD_600nm_ of 0.005. Cultures (volume, 175 ml in 1 L flasks) were grown at 37°C, under shaking conditions (200 rpm) until the OD_600nm_ reached 0.2. Three biological replicates were performed for each condition. Total RNA from bacterial pellets was extracted using TRIzol reagent method as previously described [107]. RNA qualities were determined using Agilent RNA Nano Chips (Agilent Technologies).

Genomic DNA was removed from RNA samples using TURBO DNase (Ambion) followed by a second DNase treatment with DNase I (Roche). DNase I treatment was repeated until DNA was not detected by genome-specific PCRs targeting four housekeeping genes (*trpB, thrB, purN* and *mdh*) (Additional file 1: Table S5). The RNA quality after DNase treatments was checked using Agilent RNA Nano Chips.

For RNA-seq, total RNA was reverse transcribed using SuperScript III reverse transcriptase (Invitrogen). Actinomycin D (6 μg/ml, Sigma) was added to the reaction to avoid spurious second-strand cDNA synthesis [108]. cDNA was purified using QIAquick PCR purification kit (Qiagen) and used for single stranded cDNA library construction as previously [109, 110]. FRT-seq Illumina libraries were constructed as previously described [111].

### Reference genome, sequencing, read mapping and statistic analysis

The genome of *S. flexneri* M90T was sequenced at Wellcome Trust Sanger Institute using an Illumina HiSeq 2000 sequencer. A total of 0.7 Gb sequence data, in 75-bp paired reads, was obtained (acc. no. ERS033387) and assembled *de novo* using Velvet [112]. This assembled sequence, which is rich in IS*1* elements and for which no attempt of gap closure was performed, is comprised of 501 contigs with a total size of 4.43 Mb. A M90T draft annotated genome was prepared and the annotation transferred from *S. flexneri* strain 8401 (acc. no. CP000266). Rfam searches were performed and the features identified were included in the annotation as well as *Shigella* published sRNAs [69, 70]. This draft genome was used as reference for the mapping of RNA-seq reads [113]. During the course of our study the *S. flexneri* M90T genome was published [114]. Therefore, final expression results are given using this latter locus tag systematic names for coding sequences.

RNA Sequencing was performed using an Illumina HiSeq 2000 sequencer. Raw data as well as mapped reads obtained per replicate were averaged per sample/condition and summarized, together with other interesting quality control parameters, in Additional file 1: Table S3. Processing of reads after mapping included the unmarking of duplicate reads followed by correction to allow for directional fidelity of the data [115]. Output files included per sample, a matrix of readcounts and RPKM values on both sense and antisense strands for genes as well as for automatic 50 bp+/- trimmed intergenic features created in the + strand. The R package *DESeq*, which implements negative binomial distribution statistics for RNA-seq data was used for statistical analysis [116]. A logarithmic transformed version of the count data (log(x + 1)) was used to avoid zero count values [117]. A *p* adjust value <0.05, which controls false discovery rate, was used for the cut-off calling of differential expression between conditions. Independent runs of analysis were carried out for sense and antisense directions. Ribosomal genes and repeated sequences, such as transposases or insertion sequences, were filtered out from final tables.

### Strand-specific quantitative RT-PCR and Northern blot

A StepOnePlus Real Time PCR system (Applied Biosystems) was used to monitor real-time quantitative PCR. First-strand cDNA was synthesized as previously described but using genome specific primers carrying a tag sequence in the 5′-end instead of random primers. This tag sequence was unique and not found in the genome of *S. flexneri* M90T. Subsequent PCRs were performed using Power SYBR Green PCR Master Mix (Applied Biosystems) and the tag sequence as one of the paired primers (See Additional file 1: Table S5). As a result, only cDNAs synthesized with a 5′-end tagged primer were amplified. Results are the average of triplicate experiments performed, on at least four independent occasions. Data were expressed relative to *polA* mRNA levels. To monitor the specificity, final PCR products were analyzed by melting curves. Only samples with no amplification in the control aliquots (not subjected to reverse transcription) were included in the study. The amount of transcripts was expressed as the *n*-fold difference relative to the control gene (2^-Δ*Ct*^ where Δ*Ct* represents the difference in threshold cycles between the target and control genes). Results were shown in relation to wild type 2^-ΔCt^ levels under aerobic conditions, which were referred as 1. Thus, values greater than 1 indicate increased transcription in relation to the wild-type under aerobic conditions, and lower than 1 indicate the opposite. Significant differences were detected with Mann–Whitney test; values with *P* <0.05 were considered as significant.

Northern blots were performed as previously described [118]. Radiolabeled RNA probes synthesized with the MAXIscript kit (Ambion) were used to detect specifically the sense of the RNA-targets. The primers used for probes synthesis are listed in Additional file 1: Table S5.

## Availability of supporting data

RNA-seq data has been submitted to the European Nucleotide Archive with accession code ERP003817 and the experiment has an ArrayExpress acc. no. E-ERAD-204.

## Electronic supplementary material

Additional file 1: Table S1: Chromosomal genes differentially expressed in response to anaerobic conditions and the role of FNR in the induction. This table shows the chromosomal genes differentially expressed in RNA-seq analysis in wild-type *S. flexneri* M90T grown under anaerobic conditions compared to aerobic conditions, and in Δ*fnr* mutant in relation to wild-type *S. flexneri* M90T when grown under anaerobic conditions. Genes are classified into functional categories based on the database of Clusters of Orthologous Groups (COGs). **Table S2.** FNR regulon under anaerobic conditions. This table contains all genes differentially expressed in the Δ*fnr* mutant in relation to the wild-type *S. flexneri* M90T when grown under anaerobic conditions. RNA-seq and FRT-seq results are presented. **Table S3.** Summary of mapping statistics. **Table S4.** Strains and plasmids used in this study. **Table S5.** Oligonucleotides used in this study. **Figure S1.** Characterization of M90T Δ*fnr* mutant. This figure confirms the absence of FNR in the Δ*fnr* mutant and shows the growth curve of the mutant in comparison to the wild-type strain M90T and the complemented mutant under anaerobic conditions. (DOCX 4 MB)
