# Supplementary material for: RNA-seq analysis of the influence of anaerobiosis and FNR on Shigella flexneri
Source: BMC Genomics. 2014 Jun 6;15:438. doi: 10.1186/1471-2164-15-438 (PMC4229854; doi:10.1186/1471-2164-15-438)
Supplement: Supplementary file 1 — Additional file 1: Table S1: Chromosomal genes differentially expressed in response to anaerobic conditions and the role of FNR in the induction. This table shows the chromosomal genes differentially expressed in RNA-seq analysis in wild-type S. flexneri M90T grown under anaerobic conditions compared to aerobic conditions, and in Δfnr mutant in relation to wild-type S. flexneri M90T when grown under anaerobic conditions. Genes are classified into functional categories based on the database of Clusters of Orthologous Groups (COGs). Table S2. FNR regulon under anaerobic conditions. This table contains all genes differentially expressed in the Δfnr mutant in relation to the wild-type S. flexneri M90T when grown under anaerobic conditions. RNA-seq and FRT-seq results are presented. Table S3. Summary of mapping statistics. Table S4. Strains and plasmids used in this study. Table S5. Oligonucleotides used in this study. Figure S1. Characterization of M90T Δfnr mutant. This figure confirms the absence of FNR in the Δfnr mutant and shows the growth curve of the mutant in comparison to the wild-type strain M90T and the complemented mutant under anaerobic conditions. (DOCX 4 MB) [file 12864_2013_7055_MOESM1_ESM.docx]

Table S1. Chromosomal genes differentially expressed in response to anaerobic conditions

| **ORF ID^ab^** | **Gene** | **Description** | **RNA-seq^c^ log2FC**  **WT no O_2_/O_2_** | | | **RNA-seq^c^ log2FC**  **Δ*fnr*/WT no O_2_** |
| --- | --- | --- | --- | --- | --- | --- |
|  | | | |  |  | |
| ***Metabolism*** | | | |  |  | |
| Energy production and conversion | | | |  |  | |
| SF5M90T_2223 | *napA* | probable nitrate reductase 3 | | **7.40** | **-4.85** | |
| SF5M90T_2975 | *hybA* | hydrogenase-2 small subunit | | **6.78** | **-2.34** | |
| SF5M90T_1706 | *fdnG* | formate dehydrogenase-N alpha subunit | | **6.67** | **-5.67** | |
| SF5M90T_2220 | *napB* | cytochrome c-type protein | | **6.39** | **-3.89** | |
| SF5M90T_4218 | *frdB* | fumarate reductase, anaerobic, iron-sulfur protein subunit | | **6.24** | **-2.08** | |
| SF5M90T_2707 | *hycB* | small subunit of hydrogenase-3, iron- sulfur protein | | **6.22** | **-2.96** | |
| SF5M90T_2221 | *napH* | ferredoxin-type protein: electron transfer | | **6.19** | **-4.62** | |
| SF5M90T_2976 |  | putative hydrogenase subunit | | **5.97** | **-2.49** | |
| SF5M90T_2720 | *hydN* | electron transport protein | | **5.87** | **-3.96** | |
| SF5M90T_4018 | *fumB* | fumarate hydratase class I, anaerobic | | **5.83** | **-3.65** | |
| SF5M90T_4219 | *frdA* | fumarate reductase, anaerobic, flavoprotein subunit | | **5.81** | **-2.34** | |
| SF5M90T_2222 | *napG* | ferredoxin-type protein: electron transfer | | **5.45** | **-4.47** | |
| SF5M90T_2219 | *napC* | cytochrome c-type protein | | **4.92** | **-4.09** | |
| SF5M90T_2974 | *hybB* | probable cytochrome Ni/Fe component of hydrogenase-2 | | **4.83** | **-1.92** | |
| SF5M90T_3080 | *pflB* | formate acetyltransferase 1 | | **4.79** | **-1.93** | |
| SF5M90T_4217 | *frdC* | fumarate reductase, anaerobic, membrane anchor polypeptide | | **4.67** | **-2.16** | |
| SF5M90T_3296 | *nirB* | nitrite reductase (NAD(P)H) subunit | | **4.61** | **-6.22** | |
| SF5M90T_4216 | *frdD* | fumarate reductase, anaerobic, membrane anchor polypeptide | | **4.54** |  | |
| SF5M90T_968 | *hyaA* | hydrogenase-1 small subunit | | **4.54** |  | |
| SF5M90T_567 | *citD* | citrate lyase acyl carrier protein (gamma chain) | | **4.53** | **-3.30** | |
| SF5M90T_1229 | *adhE* | CoA-linked acetaldehyde dehydrogenase and iron- dependent alcohol dehydrogenase | | **4.41** |  | |
| SF5M90T_2973 | *hybC* | probable large subunit, hydrogenase-2 | | **4.36** | **-1.75** | |
| SF5M90T_1705 | *fdnH* | formate dehydrogenase-N beta subunit | | **4.20** | **-4.23** | |
| SF5M90T_1654 |  | putative oxidoreductase, Fe-S subunit | | **4.18** |  | |
| SF5M90T_970 | *hyaC* | probable Ni/Fe-hydrogenase 1 b-type cytochrome subunit | | **4.13** |  | |
| SF5M90T_1704 | *fdnI* | formate dehydrogenase-N gamma subunit | | **4.05** | **-3.54** | |
| SF5M90T_602 | *appC* | probable third cytochrome oxidase, subunit I | | **3.96** |  | |
| SF5M90T_1519 |  | putative oxidoreductase, major subunit | | **3.80** | **-4.97** | |
| SF5M90T_4050 | *nrfB* | cytochrome c nitrite reductase pentaheme subunit | | **3.67** | **-3.28** | |
| SF5M90T_565 | *citF* | citrate lyase alpha chain | | **3.31** | **-2.88** | |
| SF5M90T_969 | *hyaB* | hydrogenase-1 large subunit | | **3.31** |  | |
| SF5M90T_3856 | *yiaY* | putative oxidoreductase | | **3.18** | **-2.73** | |
| SF5M90T_1560 |  | putative oxidoreductase, major subunit | | **3.06** |  | |
| SF5M90T_2254 | *glpA* | sn-glycerol-3-phosphate dehydrogenase (anaerobic), large subunit | | **2.88** | **-1.09** | |
| SF5M90T_2972 | *hybD* | probable processing element for hydrogenase-2 | | **2.77** | **-1.96** | |
| SF5M90T_2712 | *hycG* | hydrogenase activity | | **2.75** |  | |
| SF5M90T_1003 | *wrbA* | NAD(P)H dehydrogenase (quinone) | | **2.60** | **2.44** | |
| SF5M90T_4049 | *nrfC* | formate-dependent nitrite reductase | | **2.52** | **-3.31** | |
| SF5M90T_1217 | *narG* | nitrate reductase 1, alpha subunit | | **2.49** | **-2.63** | |
| SF5M90T_317 | *ykgF* | uncharacterized protein with a ferredoxin-like domain | | **2.49** | **1.80** | |
| SF5M90T_3204 | *yhdH* | putative dehydrogenase | | **2.44** |  | |
| SF5M90T_2711 | *hycF* | probable iron-sulfur protein of hydrogenase 3 (part of FHL complex) | | **2.36** |  | |
| SF5M90T_856 | *ybjW* | putative prismane | | **2.36** |  | |
| SF5M90T_2708 | *hycC* | membrane-spanning protein of hydrogenase 3 | | **2.30** | **-2.73** | |
| SF5M90T_1562 |  | putative oxidoreductase, Fe-S subunit | | **2.02** | **-1.98** | |
| SF5M90T_3333 | *pckA* | phosphoenolpyruvate carboxykinase | | **1.99** |  | |
| SF5M90T_3877 | *yiaK* | putative dehydrogenase | | **1.93** | **1.22** | |
| SF5M90T_2256 | *glpC* | sn-glycerol-3-phosphate dehydrogenase (anaerobic), K-small subunit | | **1.92** |  | |
| SF5M90T_3374 | *ugpQ* | glycerophosphodiester phosphodiesterase, cytosolic | | **1.89** |  | |
| SF5M90T_316 | *ykgE* | putative dehydrogenase subunit | | **1.81** | **1.26** | |
| SF5M90T_2710 | *hycE* | large subunit of hydrogenase 3 (part of FHL complex) | | **1.77** | **-1.44** | |
| SF5M90T_2252 | *glpQ* | periplasmic glycerophosphodiester phosphodiesterase | | **1.73** |  | |
| SF5M90T_2534 | *hmpA* | dihydropteridine reductase, ferrisiderophore reductase activity | | **1.47** | **5.38** | |
| SF5M90T_33 | *caiB* | l-carnitine dehydratase | | **1.32** |  | |
| SF5M90T_3679 | *atpF* | membrane-bound ATP synthase, F0 sector, subunit b | | **1.04** |  | |
| SF5M90T_3680 | *atpE* | membrane-bound ATP synthase, F0 sector, subunit c | | **1.04** |  | |
| SF5M90T_2304 | *ackA* | acetate kinase | | **0.94** | **-1.33** | |
| SF5M90T_602 | *cydA* | cytochrome d terminal oxidase, polypeptide subunit I | | **0.93** | **2.14** | |
| SF5M90T_3937 | *ppc* | phosphoenolpyruvate carboxylase | | **0.91** |  | |
| SF5M90T_579 | *galT* | galactose-1-phosphate uridylyltransferase | | **0.77** |  | |
| SF5M90T_3523 | *fdoH* | formate dehydrogenase-O, iron-sulfur subunit | | **-0.98** | **2.65** | |
| SF5M90T_1419 | *ydjA* | predicted oxidoreductase | | **-1.17** | **1.69** | |
| SF5M90T_2771 | *ygaF* | hydroxyglutarate oxidase | | **-1.31** | **4.12** | |
| SF5M90T_4044 | *gltP* | glutamate-aspartate symport protein | | **-1.32** |  | |
| SF5M90T_614 | *gltA* | citrate synthase | | **-1.39** | **2.96** | |
| SF5M90T_1603 | *rnfB* | electron transport complex protein | | **-1.46** |  | |
| SF5M90T_2869 | *fldB* | flavodoxin 2 | | **-1.56** |  | |
| SF5M90T_109 | *acnB* | aconitate hydrase B | | **-1.58** | **1.69** | |
| SF5M90T_1602 | *rnfA* | Na+-translocating NADH-quinone reductase subunit E | | **-1.75** |  | |
| SF5M90T_106 | *aceF* | pyruvate dehydrogenase (dihydrolipoyltransacetylase component) | | **-1.95** | **2.43** | |
| SF5M90T_3840 | *lldP* | L-lactate permease | | **-2.11** | **6.41** | |
| SF5M90T_105 | *aceE* | pyruvate dehydrogenase (decarboxylase component) | | **-2.14** | **2.25** | |
| SF5M90T_610 | *sdhB* | succinate dehydrogenase, iron sulfur protein | | **-2.31** | **3.61** | |
| SF5M90T_611 | *sdhA* | succinate dehydrogenase, flavoprotein subunit | | **-2.34** | **4.49** | |
| SF5M90T_1151 | *icdA* | isocitrate dehydrogenase, specific for NADP+ | | **-2.35** | **1.73** | |
| SF5M90T_1745 | *aldA* | aldehyde dehydrogenase, NAD-linked | | **-2.47** |  | |
| SF5M90T_3946 | *udhA* | soluble pyridine nucleotide transhydrogenase | | **-2.69** | **2.16** | |
| SF5M90T_612 | *sdhD* | succinate dehydrogenase, hydrophobic subunit | | **-2.88** | **4.61** | |
| SF5M90T_1585 | *fumC* | fumarate hydratase | | **-2.99** | **2.55** | |
| SF5M90T_1011 | *rutA* | pyrimidine monooxygenase | | **-3.07** |  | |
| SF5M90T_613 | *sdhC* | succinate dehydrogenase, cytochrome b556 | | **-3.15** | **4.54** | |
| SF5M90T_322 | *betB* | NAD+-dependent betaine aldehyde dehydrogenase | | **-3.50** | **3.19** | |
| SF5M90T_3356 | *glpD* | sn-glycerol-3-phosphate dehydrogenase (aerobic) | | **-3.64** | **3.12** | |
|  |  |  | |  |  | |
| Carbohydrate transport and metabolism | | | |  |  | |
| SF5M90T_566 | *citE* | citrate lyase beta chain (acyl lyase subunit) | | **4.69** | **-3.43** | |
| SF4250 | *treB* | PTS system trehalose(maltose)-specific transporter subunits IIBC | | **3.66** |  | |
| SF5M90T_4160 | *treC* | trehalase 6-P hydrolase | | **3.56** |  | |
| SF5M90T_1809 | *pykA* | pyruvate kinase | | **3.39** | **-1.57** | |
| SF5M90T_1379 | *manX* | PTS enzyme IIAB, mannose-specific | | **3.36** |  | |
| SF5M90T_1378 | *manY* | PTS enzyme IIC, mannose-specific | | **3.11** |  | |
| SF5M90T_1377 | *manZ* | PTS enzyme IID, mannose-specific | | **2.89** |  | |
| SF5M90T_991 | *torT* | TMAO reductase system periplasmic protein | | **2.89** |  | |
| SF5M90T_1463 | *pfkB* | 6-phosphofructokinase II | | **2.76** | **1.62** | |
| SF5M90T_3670 | *rbsD* | high affinity ribose transport protein | | **2.71** |  | |
| SF5M90T_1192 |  | dihydroxyacetone kinase subunit DhaK | | **2.41** | **2.03** | |
| SF5M90T_2253 | *glpT* | sn-glycerol-3-phosphate permease | | **2.28** |  | |
| SF5M90T_1101 | *ptsG* | PTS system, glucose-specific IIBC component | | **2.27** |  | |
| SF5M90T_3491 | *treF* | cytoplasmic trehalase | | **2.12** |  | |
| SF5M90T_2419 | *murP* | PTS system N-acetylmuramic acid transporter subunits EIIBC | | **2.09** |  | |
| SF5M90T_3499 | *pfkA* | 6-phosphofructokinase I | | **2.08** |  | |
| SF5M90T_2096 |  | fructose-bisphosphate aldolase | | **2.02** | **2.08** | |
| SF5M90T_1001 | *agp* | periplasmic glucose-1-phosphatase | | **2.00** | **1.40** | |
| SF5M90T_2606 | *eno* | enolase | | **1.93** |  | |
| SF5M90T_1190 | *ycgC* | putative PTS system enzyme I | | **1.90** | **1.83** | |
| SF5M90T_1191 |  | dihydroxyacetone kinase subunit DhaL | | **1.84** | **1.92** | |
| SF5M90T_2887 | *rpiA* | ribosephosphate isomerase, constitutive | | **1.84** |  | |
| SF5M90T_2898 | *pgk* | phosphoglycerate kinase | | **1.84** |  | |
| SF5M90T_1403 | *gapA* | glyceraldehyde-3-phosphate dehydrogenase A | | **1.83** |  | |
| SF5M90T_2097 | *yegT* | putative nucleoside permease protein | | **1.74** |  | |
| SF5M90T_2897 | *fba* | fructose-bisphosphate aldolase, class II | | **1.56** |  | |
| SF5M90T_2404 | *ptsH* | PTS system protein HPr | | **1.56** |  | |
| SF5M90T_4098 | *pgi* | glucosephosphate isomerase | | **1.53** |  | |
| SF5M90T_3850 | *mtlA* | PTS system, mannitol-specific enzyme IIABC components | | **1.52** |  | |
| SF5M90T_1640 | *ydhC* | putative transport protein | | **1.51** |  | |
| SF5M90T_2359 |  | beta-fructosidase | | **1.49** |  | |
| SF5M90T_3496 | *tpiA* | triosephosphate isomerase | | **1.45** |  | |
| SF5M90T_2405 | *ptsI* | PEP-protein phosphotransferase system enzyme I | | **1.42** |  | |
| SF5M90T_2808 | *fucI* | L-fucose isomerase | | **1.41** |  | |
| SF5M90T_2875 | *bglA* | 6-phospho-beta-glucosidase A | | **1.27** |  | |
| SF5M90T_3348 | *malP* | maltodextrin phosphorylase | | **1.16** |  | |
| SF5M90T_1107 | *ycfO* | beta-hexosaminidase | | **1.13** |  | |
| SF5M90T_8 | *talB* | transaldolase B | | **1.10** |  | |
| SF5M90T_1587 | *manA* | mannose-6-phosphate isomerase | | **1.04** |  | |
| SF5M90T_1657 | *pykF* | pyruvate kinase | | **1.01** |  | |
| SF5M90T_2033 | *gnd* | gluconate-6-phosphate dehydrogenase | | **1.01** |  | |
| SF5M90T_581 | *galM* | galactose-1-epimerase | | **1.01** | **1.41** | |
| SF5M90T_1805 | *eda* | keto-hydroxyglutarate-aldolase/keto-deoxy-phosp hogluconate aldolase | | **0.97** |  | |
| SF5M90T_580 | *galK* | galactokinase | | **0.95** |  | |
| SF5M90T_2913 | *tktA* | transketolase 1 isozyme | | **0.80** |  | |
| SF5M90T_2187 | *fruB* | PTS system fructose-specific transporter subunit IIA/HPr protein | | **-1.33** |  | |
| SF5M90T_1044 | *ymdD* | glucans biosynthesis protein C | | **-1.35** |  | |
| SF5M90T_2186 | *fruK* | fructose-1-phosphate kinase | | **-1.75** |  | |
| SF5M90T_3161 | *ptsO* | phosphocarrier protein NPr | | **-1.76** |  | |
| SF5M90T_1637 |  | putative transport protein | | **-1.93** | **1.58** | |
| SF5M90T_582 | *gpmA* | phosphoglyceromutase 1 | | **-1.97** | **2.38** | |
| SF5M90T_2185 | *fruA* | PTS system, fructose-specific transport protein | | **-1.99** |  | |
| SF5M90T_2582 | *kgtP* | alpha-ketoglutarate permease | | **-3.00** |  | |
| SF5M90T_115 | *gcd* | glucose dehydrogenase | | **-4.17** | **2.96** | |
|  | | | | |  | |
| Aminoacid transport and metabolism | | | | |  | |
| SF5M90T_4201 | *aspA* | aspartate ammonia-lyase (aspartase) | | **5.37** | **-2.59** | |
| SF5M90T_3401 | *nikA* | periplasmic binding protein for nickel | | **3.57** | **-5.12** | |
| SF5M90T_4023 | *adiA* | biodegradative arginine decarboxylase | | **3.19** | **-1.15** | |
| SF5M90T_4025 | *yjdE* | putative amino acid/amine transport protein, cryptic | | **3.11** | **-1.27** | |
| SF5M90T_2941 | *ansB* | periplasmic L-asparaginase II | | **3.03** | **-4.49** | |
| SF5M90T_3082 | *tdcC* | anaerobically inducible L-threonine, L-serine permease | | **3.01** |  | |
| SF5M90T_2403 | *cysK* | cysteine synthase A | | **3.00** | **-1.15** | |
| SF5M90T_455 | *ybaS* | putative glutaminase | | **2.82** | **3.98** | |
| SF5M90T_1125 | *pepT* | putative peptidase T | | **2.82** | **-2.66** | |
| SF5M90T_4003 | *pepE* | peptidase E | | **2.75** | **-2.09** | |
| SF5M90T_2255 | *glpB* | sn-glycerol-3-phosphate dehydrogenase (anaerobic), membrane anchor subunit | | **2.43** | **-2.51** | |
| SF5M90T_1689 | *gadB* | glutamate decarboxylase isozyme | | **2.27** | **5.19** | |
| SF5M90T_2823 | *argA* | N-acetylglutamate synthase | | **1.94** |  | |
| SF5M90T_1910 | *fliY* | putative periplasmic binding transport protein | | **1.80** |  | |
| SF5M90T_2802 | *sdaC* | probable serine transporter | | **1.69** | **-2.23** | |
| SF5M90T_625 | *ybgH* | peptide transporter | | **1.64** | **-1.60** | |
| SF5M90T_292 | *pepD* | aminoacyl-histidine dipeptidase (peptidase D) | | **1.54** | **1.78** | |
| SF5M90T_2879 | *gcvT* | aminomethyltransferase | | **1.53** |  | |
| SF5M90T_284 | *proA* | gamma-glutamylphosphate reductase | | **1.48** |  | |
| SF5M90T_1121 | *potD* | spermidine/putrescine periplasmic transport protein | | **1.44** |  | |
| SF5M90T_2674 | *cysD* | ATP:sulfurylase (ATP:sulfate adenylyltransferase), subunit 2 | | **1.39** | **-2.30** | |
| SF5M90T_1514 | *dcp* | dipeptidyl carboxypeptidase II | | **1.35** |  | |
| SF5M90T_285 | *proB* | gamma-glutamate kinase | | **1.26** |  | |
| SF5M90T_2533 | *glyA* | serine hydroxymethyltransferase | | **1.16** |  | |
| SF5M90T_2967 | *gsp* | glutathionylspermidine synthetase/amidase | | **1.16** |  | |
| SF5M90T_1806 | *edd* | 6-phosphogluconate dehydratase | | **1.15** |  | |
| SF5M90T_807 |  | glutathione transporter ATP-binding protein | | **1.08** |  | |
| SF5M90T_2317 | *hisJ* | histidine-binding periplasmic protein of high- affinity histidine transport system | | **1.05** |  | |
| SF5M90T_806 | *ybiK* | putative asparaginase | | **1.02** |  | |
| SF5M90T_1122 | *potC* | spermidine/putrescine transport system permease | | **0.97** | **-1.09** | |
| SF5M90T_2882 | *pepP* | proline aminopeptidase P II | | **0.94** |  | |
| SF5M90T_2877 | *gcvP* | glycine decarboxylase | | **0.90** | **2.13** | |
| SF5M90T_3687 | *asnA* | asparagine synthetase A | | **-1.23** |  | |
| SF5M90T_4099 | *lysC* | aspartokinase III, lysine sensitive | | **-1.33** | **1.37** | |
| SF5M90T_111 | *speD* | S-adenosylmethionine decarboxylase | | **-1.34** |  | |
| SF5M90T_112 | *speE* | spermidine synthase | | **-1.44** |  | |
| SF5M90T_1286 |  | putative glutamine synthetase | | **-1.49** |  | |
| SF5M90T_1253 | *trpE* | anthranilate synthase component I | | **-1.57** |  | |
| SF5M90T_4187 | *cycA* | transport of D-alanine, D-serine, and glycine | | **-1.69** |  | |
| SF5M90T_1946 | *yedA* | putative transmembrane subunit | | **-1.79** |  | |
| SF5M90T_3626 | *yifK* | putative amino acid/amine transport protein | | **-1.94** |  | |
| SF5M90T_3385 | *livJ* | Leu/Ile/Val-binding protein precursor | | **-1.95** |  | |
| SF5M90T_2843 | *lysA* | diaminopimelate decarboxylase | | **-2.11** | **2.44** | |
| SF5M90T_4029 | *proP* | low-affinity transport system; proline permease II | | **-2.65** | **1.05** | |
| SF5M90T_321 | *betA* | choline dehydrogenase | | **-2.76** | **2.87** | |
| SF5M90T_4185 | *ytfF* | putative transmembrane subunit | | **-3.70** | **2.71** | |
|  | | | | |  | |
| Nucleotide transport and metabolism | | | | |  | |
| SF5M90T_4161 | *nrdD* | anaerobic ribonucleoside-triphosphate reductase | | **4.18** | **-2.74** | |
| SF5M90T_3587 | *udp* | uridine phosphorylase | | **3.12** |  | |
| SF5M90T_2161 | *cdd* | cytidine/deoxycytidine deaminase | | **2.82** |  | |
| SF5M90T_2387 | *nupC* | permease of transport system for 3 nucleosides | | **2.81** |  | |
| SF5M90T_2949 | *nupG* | nucleoside permease | | **2.47** |  | |
| SF5M90T_4321 | *deoC* | 2-deoxyribose-5-phosphate aldolase | | **2.40** |  | |
| SF5M90T_4322 | *deoA* | thymidine phosphorylase | | **2.39** |  | |
| SF5M90T_674 | *ybeK* | putative tRNA synthetase | | **1.60** |  | |
| SF5M90T_444 | *adk* | adenylate kinase | | **1.51** |  | |
| SF5M90T_2456 | *purC* | phosphoribosylaminoimidazole-succinocarboxamidesynthetase | | **1.35** |  | |
| SF5M90T_4182 | *cpdB* | 2':3'-cyclic-nucleotide 2'-phosphodiesterase | | **1.16** |  | |
| SF5M90T_291 | *gpt* | guanine-hypoxanthine phosphoribosyltransferase | | **1.10** |  | |
| SF5M90T_1598 | *add* | adenosine deaminase | | **0.95** |  | |
| SF5M90T_2249 | *nrdB* | ribonucleoside-diphosphate reductase 1, beta subunit, B2 | | **-1.36** |  | |
| SF5M90T_478 | *purE* | phosphoribosylaminoimidazole carboxylase | | **-1.41** |  | |
| SF5M90T_2248 | *nrdA* | ribonucleoside diphosphate reductase 1, alpha subunit, B1 | | **-2.97** | **2.35** | |
|  | | | |  |  | |
| Coenzyme transport and metabolism | | | |  |  | |
| SF5M90T_1566 | *bioD* | ATP-dependent dethiobiotin synthetase | | **4.42** | **-3.78** | |
| SF5M90T_2274 | *menB* | dihydroxynaphtoic acid synthetase | | **2.63** | **-1.75** | |
| SF5M90T_2687 |  | phenylacrylic acid decarboxylase-like protein | | **1.83** |  | |
| SF5M90T_2276 | *menD* | 2-oxoglutarate decarboxylase | | **1.76** | **-1.63** | |
| SF5M90T_2273 | *menC* | o-succinylbenzoate synthase | | **1.76** | **-1.72** | |
| SF5M90T_761 | *moaC* | molybdopterin biosynthesis, protein C | | **1.14** | **-0.98** | |
| SF5M90T_3142 | *ispB* | octaprenyl diphosphate synthase | | **1.06** |  | |
| SF5M90T_1613 | *pdxH* | pyridoxinephosphate oxidase | | **1.06** |  | |
| SF5M90T_2880 | *visC* | putative FAD-dependent oxidoreductase | | **0.89** |  | |
| SF5M90T_3011 | *ribB* | 3,4 dihydroxy-2-butanone-4-phosphate synthase | | **-1.10** |  | |
| SF5M90T_3577 | *yigC* | putative oxidoreductase | | **-1.31** |  | |
| SF5M90T_2885 | *ygfA* | putative ligase | | **-1.59** |  | |
| SF5M90T_3957 | *birA* | biotin--protein ligase | | **-1.62** |  | |
| SF5M90T_2103 | *thiM* | hydoxyethylthiazole kinase | | **-1.95** |  | |
|  | | | |  |  | |
| Lipid transport and metabolism | | | |  |  | |
| SF5M90T_1094 | *acpP* | acyl carrier protein | | **1.70** | **-3.04** | |
| SF5M90T_2272 | *menE* | o-succinylbenzoate-CoA ligase | | **1.60** | **-1.54** | |
| SF5M90T_2416 | *ucpA* | putative oxidoreductase | | **1.45** |  | |
| SF5M90T_3112 | *yhbT* | putative lipid carrier protein | | **1.40** | **-1.33** | |
| SF5M90T_2351 | *fadL* | Long-chain fatty acid transport protein precursor | | **-1.46** | **-1.87** | |
| SF5M90T_339 | *sbmA* | sensitivity to microcin B17, possibly envelope protein | | **-1.64** |  | |
|  | | | |  |  | |
| Inorganic ion transport and metabolism | | | |  |  | |
| SF2292 | *napF* | ferredoxin-type protein | | **6.94** | **-4.73** | |
| SF5M90T_2224 | *napD* | assembly protein for periplasmic nitrate reductase | | **6.38** | **-4.52** | |
| SF5M90T_3405 | *nikE* | ATP-binding protein of nickel transport system | | **5.00** | **-4.43** | |
| SF5M90T_3404 | *nikD* | ATP-binding protein of nickel transport system | | **4.82** | **-4.29** | |
| SF5M90T_3402 | *nikB* | transport of nickel, membrane protein | | **4.52** | **-4.38** | |
| SF5M90T_3403 | *nikC* | transport of nickel, membrane protein | | **4.39** | **-4.66** | |
| SF5M90T_3297 | *nirD* | nitrite reductase (NAD(P)H) subunit | | **4.38** | **-5.58** | |
| SF5M90T_3298 | *nirC* | Nitrite transporter | | **4.24** | **-4.86** | |
| SF5M90T_4051 | *nrfA* | cytochrome c552 precursor | | **4.04** | **-3.68** | |
| SF5M90T_1895 | *ftn* | Ferritin 1 | | **3.96** |  | |
| SF5M90T_895 | *focA* | probable formate transporter (formate channel 1) | | **3.42** | **-2.16** | |
| SF5M90T_2903 |  | hypothetical lipoprotein | | **3.41** |  | |
| SF5M90T_929 | *ycbO* | alkanesulfonate transporter substrate-binding subunit | | **3.04** |  | |
| SF5M90T_2415 | *cysP* | thiosulfate binding protein | | **2.81** | **-1.51** | |
| SF5M90T_1636 | *sodB* | superoxide dismutase | | **2.52** | **2.13** | |
| SF5M90T_1187 |  | putative ATP-binding protein of ABC transporter | | **2.14** |  | |
| SF5M90T_791 | *dps* | global regulator, starvation conditions | | **2.05** | **1.98** | |
| SF5M90T_3924 | *katG* | catalase; hydroperoxidase HPI(I) | | **1.95** |  | |
| SF5M90T_454 | *copA* | copper exporting ATPase | | **1.95** |  | |
| SF5M90T_1186 |  | putative iron compound ABC transporter permease | | **1.69** |  | |
| SF5M90T_1185 |  | iron ABC transporter ATP-binding protein | | **1.52** |  | |
| SF5M90T_4057 | *yjcE* | predicted cation/proton antiporter | | **1.49** |  | |
| SF5M90T_1198 | *ychM* | C4-dicarboxyl acid transporter | | **1.41** |  | |
| SF5M90T_2675 | *cysN* | ATP-sulfurylase (ATP:sulfate adenylyltransferase), subunit 1 | | **1.08** | **-2.08** | |
| SF5M90T_2420 | *yfeX* | deferrochelatase/peroxidase | | **1.02** | **-0.92** | |
| SF5M90T_448 | *ybaL* | putative transport protein | | **-1.01** |  | |
| SF5M90T_1795 |  | putative resistance protein | | **-1.49** | **1.47** | |
| SF5M90T_3355 | *glpE* | thiosulfate sulfurtransferase | | **-1.64** |  | |
| SF5M90T_2386 | *mntH* | divalent metal cation transporter | | **-1.93** | **1.61** | |
| SF5M90T_330 | *tauC* | taurine transport system permease protein | | **-2.09** |  | |
| SF5M90T_3769 | *shiF* | putative membrane transport protein | | **-2.16** |  | |
| SF5M90T_117 | *yadF* | putative carbonic anhdrase | | **-2.17** | **1.49** | |
| SF5M90T_1209 | *chaA* | sodium-calcium/proton antiporter | | **-2.22** |  | |
| SF5M90T_3764 | *iutA* | putative ferric siderophore receptor | | **-2.25** |  | |
| SF5M90T_1660 | *sufE* | cysteine desulfuration protein | | **-2.39** | **1.42** | |
| SF5M90T_3054 | *ygjT* | putative transport protein | | **-2.41** |  | |
| SF5M90T_138 | *fhuB* | hydroxamate-dependent iron uptake, cytoplasmic membrane component | | **-2.43** |  | |
| SF5M90T_1102 | *fhuE* | outer membrane receptor for ferric iron uptake | | **-2.46** |  | |
| SF5M90T_1573 | *mdtJ* | spermidine export protein | | **-2.74** |  | |
| SF5M90T_537 | *fepB* | ferrienterobactin-binding periplasmic protein precursor | | **-2.80** |  | |
| SF5M90T_1483 | *ydiE* | hemin uptake protein | | **-2.93** | **-2.20** | |
| SF5M90T_3765 | *iucD* | lysine:N6-hydroxylase | | **-2.96** |  | |
| SF5M90T_135 | *fhuA* | outer membrane protein receptor for ferrichrome | | **-3.00** |  | |
| SF5M90T_1661 | *sufS* | cysteine desulfurase | | **-3.15** | **2.52** | |
| SF5M90T_1345 | *sitD* | iron transport protein, inner membrane component | | **-3.27** |  | |
| SF5M90T_3766 | *iucC* | siderophore biosynthesis protein | | **-3.47** |  | |
| SF5M90T_1572 | *mdtI* | spermidine export protein | | **-3.61** |  | |
| SF5M90T_4306 | *fhuF* | ferric hydroximate transport ferric iron reductase | | **-3.61** |  | |
| SF5M90T_534 | *fepG* | ferric enterobactin transport protein | | **-3.90** |  | |
| SF5M90T_3768 | *iucA* | siderophore biosynthesis protein | | **-4.06** |  | |
| SF5M90T_533 | *fepC* | ATP-binding component of ferric enterobactin transport | | **-4.10** |  | |
| SF5M90T_1346 | *sitC* | Iron transport protein | | **-4.21** |  | |
| SF5M90T_3767 | *iucB* | siderophore biosynthesis protein | | **-4.23** |  | |
| SF5M90T_1347 | *sitB* | iron transport protein, ATP-binding component | | **-4.38** |  | |
| SF5M90T_1348 | *sitA* | iron transport protein, periplasmic-binding protein | | **-4.87** |  | |
| SF5M90T_3508 | *sodA* | superoxide dismutase, manganese | | **-5.83** | **1.22** | |
|  | | | | |  | |
| Secondary metabolites biosynthesis, transport and catabolism | | | | |  | |
| SF5M90T_1184 |  | putative SAM-dependent methyltransferase | | **2.12** |  | |
| SF5M90T_32 | *caiC* | probable crotonobetaine/carnitine-CoA ligase | | **2.09** |  | |
| SF5M90T_529 | *entF* | enterobactin synthetase component F | | **-1.95** |  | |
| SF5M90T_331 | *tauD* | taurine dioxygenase, 2-oxoglutarate-dependent | | **-2.97** | **1.94** | |
|  |  |  | |  |  | |
| ***Cellular processes and signalling*** | | | |  |  | |
| Cell cycle control, cell division, chromosome partitioning | | | |  |  | |
| SF5M90T_1243 | *yciB* | probable intracellular septation protein A | | **0.93** |  | |
|  | | | |  |  | |
| Defense mechanisms | | | |  |  | |
| SF5M90T_3444 | *hdeB* | acid stress chaperone | | **5.63** |  | |
| SF5M90T_3445 | *hdeA* | acid stress chaperone | | **4.60** | **1.67** | |
| SF5M90T_3446 | *hdeD* | acid-resistance membrane protein | | **3.79** | **2.53** | |
| SF5M90T_3901 | *gadA* | glutamate decarboxylase isozyme | | **2.03** | **5.14** | |
| SF5M90T_4215 | *ampC* | beta-lactamase; penicillin resistance | | **1.55** |  | |
| SF5M90T_3751 | *emrD* | multidrug resistance protein D | | **1.41** |  | |
| SF5M90T_4273 |  | putative restriction modification enzyme R subunit | | **1.41** | **-0.94** | |
| SF5M90T_3781 | *shiA* | virulence factor | | **1.30** |  | |
| SF5M90T_101 | *ampD* | N-acetyl-anhydromuranmyl-L-alanine amidase | | **1.18** |  | |
| SF5M90T_772 | *ybhF* | putative ABC-type multidrug transport system component | | **1.16** |  | |
| SF5M90T_771 | *ybhS* | putative ABC-type multidrug transport system component | | **1.15** |  | |
| SF5M90T_770 | *ybhR* | putative ABC-type multidrug transport system component | | **0.90** |  | |
| SF5M90T_418 | *mdlA* | ATP-binding component of a transport system | | **-1.29** |  | |
| SF5M90T_2227 | *yojI* | putative ATP-binding component of a transport system | | **-3.82** |  | |
|  | | | |  |  | |
| Signal transduction mechanisms | | | |  |  | |
| SF5M90T_814 | *bssR* | biofilm formation regulatory protein | | **3.76** | **-2.00** | |
| SF5M90T_558 | *ybdQ* | universal stress protein | | **1.99** |  | |
| SF5M90T_2126 | *yehU* | putative 2-component sensor protein | | **1.36** |  | |
| SF5M90T_3428 | *uspA* | universal stress protein | | **0.86** |  | |
| SF5M90T_2388 | *yfeA* | predicted diguanylate cyclase | | **-1.20** |  | |
| SF5M90T_1779 | *fnr* | fumarate and nitrate reduction regulatory protein | | **-1.36** | **-3.51** | |
| SF5M90T_4339 | *creC* | sensory histidine kinase | | **-1.63** | **1.43** | |
| SF5M90T_4279 | *yjiY* | putative carbon starvation protein | | **-3.46** | **2.66** | |
|  | | | |  |  | |
| Cell wall/membrane/envelope biogenesis | | | |  |  | |
| SF5M90T_1245 | *yciD* | putative outer membrane protein | | **4.06** |  | |
| SF5M90T_3441 | *slp* | outer membrane protein induced after carbon starvation | | **3.41** | **2.74** | |
| SF5M90T_1923 | *nmpC* | outer membrane porin protein | | **2.04** | **-1.34** | |
| SF5M90T_1618 | *slyB* | putative outer membrane protein | | **1.82** | **-1.43** | |
| SF5M90T_952 | *ompA* | outer membrane protein 3a | | **1.59** |  | |
| SF5M90T_374 | *tsx* | outer membrane protein | | **1.53** |  | |
| SF5M90T_1641 | *cfa* | cyclopropane fatty acyl phospholipid synthase | | **1.40** |  | |
| SF5M90T_256 | *gtrB* | bactoprenol glucosyl transferase | | **1.36** | **-2.59** | |
| SF5M90T_2039 | *rfbC* | dTDP-4-dehydrorhamnose 3,5-epimerase | | **1.20** | **-2.64** | |
| SF5M90T_2231 | *ompC* | outer membrane protein 1b | | **1.12** |  | |
| SF5M90T_4332 | *slt* | soluble lytic murein transglycosylase | | **0.96** | **1.54** | |
| SF5M90T_3951 | *murI* | glutamate racemase | | **0.93** |  | |
| SF5M90T_3821 | *rfaD* | ADP-L-glycero-D-mannoheptose-6-epimerase | | **0.82** |  | |
| SF5M90T_2373 | *ddg* | putative heat shock protein | | **-1.55** | **1.52** | |
| SF5M90T_1241 | *tonB* | transport protein | | **-1.72** |  | |
| SF5M90T_3956 | *murB* | UDP-N-acetylenolpyruvoylglucosamine reductase | | **-2.23** |  | |
|  |  |  | |  |  | |
| Cell motility |  |  | |  |  | |
| SF5M90T_1938 | *fliQ* | flagellar biosynthetic protein | | **3.55** |  | |
|  | | | |  |  | |
| Intracellular trafficking, secretion and vesicular transport | | | |  |  | |
| SF5M90T_698 | *tatE* | probable sec-independent protein translocase | | **2.42** |  | |
| SF5M90T_3964 | *secE* | preprotein translocase | | **0.87** | **-1.06** | |
| SF5M90T_3580 | *tatC* | sec-independent protein translocase | | **0.84** |  | |
| SF5M90T_2982 | *exbD* | biopolymer transport | | **-2.57** | **-1.15** | |
| SF5M90T_2983 | *exbB* | biopolymer transport | | **-3.01** |  | |
| SF5M90T_3501 | *yiiO* | uncharacterized periplasmic protein | | **-4.72** |  | |
|  | | | |  |  | |
| Posttranslational modification, protein turnover, chaperones | | | |  |  | |
| SF5M90T_3113 | *yhbU* | putative collagenase | | **6.80** | **-6.25** | |
| SF5M90T_2704 | *hypB* | hydrogenase nickel incorporation protein | | **5.58** | **-3.05** | |
| SF5M90T_3114 | *yhbV* | predicted protease | | **5.37** | **-5.06** | |
| SF5M90T_2703 | *hypC* | hydrogenase assembly chaperone | | **5.08** | **-3.16** | |
| SF5M90T_972 | *hyaE* | processing of HyaA and HyaB proteins | | **4.68** |  | |
| SF5M90T_2702 | *hypD* | hydrogenase isoenzyme | | **4.18** | **-2.96** | |
| SF5M90T_2701 | *hypE* | hydrogenase maturation protein | | **3.95** | **-2.14** | |
| SF5M90T_2217 | *ccmB* | heme exporter protein B, cytochrome c-type biogenesis protein | | **3.91** | **-2.29** | |
| SF5M90T_2218 | *ccmA* | ATP binding protein of heme exporter A | | **3.85** | **-3.35** | |
| SF5M90T_4162 | *nrdG* | anaerobic ribonucleotide reductase activating protein | | **3.70** | **-2.81** | |
| SF5M90T_4319 | *yjjW* | putative activating enzyme | | **3.68** | **-3.10** | |
| SF5M90T_2721 | *hypF* | transcriptional regulatory protein | | **3.40** | **-2.66** | |
| SF5M90T_2971 | *hybE* | hydrogenase 2-specific chaperone | | **3.24** | **-1.82** | |
| SF5M90T_2213 | *ccmF* | cytochrome c-type biogenesis protein | | **3.11** | **-2.11** | |
| SF5M90T_2214 | *ccmE* | cytochrome c-type biogenesis protein | | **3.07** | **-2.44** | |
| SF5M90T_893 | *pflA* | pyruvate formate lyase activating enzyme 1 | | **2.89** |  | |
| SF5M90T_2216 | *ccmC* | heme exporter protein C | | **2.70** | **-2.85** | |
| SF5M90T_2212 | *dsbE* | thiol:disulfide interchange protein | | **2.68** | **-2.09** | |
| SF5M90T_2211 | *ccmH* | possible subunit of heme lyase | | **2.38** | **-2.13** | |
| SF5M90T_2591 | *clpB* | heat shock protein | | **2.25** |  | |
| SF5M90T_999 | *cbpA* | curved DNA-binding protein | | **1.82** | **1.53** | |
| SF5M90T_998 | *yccD* | chaperone modulatory protein CbpM | | **1.42** | **2.01** | |
| SF5M90T_4204 | *mopB* | co-chaperonin GroES | | **1.41** |  | |
| SF5M90T_3279 | *slyD* | FKBP-type peptidyl-prolyl cis-trans isomerase | | **1.31** |  | |
| SF5M90T_4106 | *iadA* | isoaspartyl dipeptidase | | **0.97** |  | |
| SF5M90T_462 | *ybbN* | putative thioredoxin-like protein | | **0.90** |  | |
| SF5M90T_407 | *clpP* | ATP-dependent proteolytic subunit of clpA-clpP serine protease | | **0.84** |  | |
| SF5M90T_3738 | *ibpA* | heat shock protein | | **-1.17** |  | |
| SF5M90T_1314 | *tpx* | thiol peroxidase | | **-1.43** | **2.29** | |
| SF5M90T_2577 | *trxC* | putative thioredoxin-like protein | | **-2.09** | **1.80** | |
| SF5M90T_2074 | *yegD* | putative heat shock protein | | **-2.80** |  | |
| SF5M90T_1665 | *sufA* | iron-sulfur cluster assembly scaffold protein | | **-3.15** | **2.24** | |
| SF5M90T_1663 | *sufC* | cysteine desulfurase ATPase component | | **-3.26** | **2.73** | |
| SF5M90T_1662 | *sufD* | cysteine desulfurase activator complex subunit | | **-3.47** | **3.01** | |
| SF5M90T_1664 | *sufB* | cysteine desulfurase activator complex subunit | | **-3.68** | **3.00** | |
|  |  |  | |  |  | |
| ***Information storage and processing*** | | | |  |  | |
| Translation, ribosomal structure and biogenesis | | | |  |  | |
| SF5M90T_2801 | *yfiA* | translation inhibitor protein RaiA | | **2.70** |  | |
| SF5M90T_2392 | *gltX* | glutamate tRNA synthetase, catalytic subunit | | **1.65** |  | |
| SF5M90T_155 | *frr* | ribosome releasing factor | | **1.21** |  | |
| SF5M90T_650 | *glnS* | glutamine tRNA synthetase | | **1.08** |  | |
| SF5M90T_3893 | *glyQ* | glycine tRNA synthetase, alpha subunit | | **1.06** |  | |
| SF5M90T_4220 | *yjeA* | putative lysyl-tRNA synthetase | | **0.95** | **-1.75** | |
| SF5M90T_3894 | *glyS* | glycine tRNA synthetase, beta subunit | | **0.81** |  | |
|  |  |  | |  |  | |
| Transcription |  |  | |  |  | |
| SF5M90T_2706 | *hycA* | transcriptional repression of hyc and hyp operons | | **6.63** | **-4.36** | |
| SF5M90T_4024 | *adiY* | putative ARAC-type regulatory protein | | **4.81** |  | |
| SF5M90T_3447 | *yhiE* | putative DNA binding transcription factor | | **3.27** |  | |
| SF5M90T_3025 | *ygiP* | putative transcriptional regulator/ nucleoid-associated protein | | **3.04** | **-4.57** | |
| SF5M90T_2417 | *murR* | HTH-type transcriptional regulator | | **2.35** |  | |
| SF5M90T_4341 | *arcA* | aerobic respiration control protein | | **2.33** | **-1.54** | |
| SF5M90T_3510 | *rhaR* | positive regulator for rhaRS operon | | **2.33** |  | |
| SF5M90T_2297 | *lrhA* | NADH dehydrogenase transcriptional regulator, LysR family | | **2.02** |  | |
| SF5M90T_3406 | *yhhG* | nickel-responsive regulator | | **2.01** | **-1.30** | |
| SF5M90T_1595 | *malI* | repressor of *malX* and *Y* genes | | **1.98** |  | |
| SF5M90T_2700 | *fhlA* | formate hydrogenlyase transcriptional activator | | **1.81** |  | |
| SF5M90T_2125 | *yehT* | putative two-component response regulator | | **1.80** |  | |
| SF5M90T_1373 | *cspC* | cold shock protein | | **1.59** | **-1.38** | |
| SF5M90T_3349 | *malT* | positive regulator of *mal* regulon | | **1.58** |  | |
| SF5M90T_3335 | *ompR* | osmolarity response regulator | | **1.42** |  | |
| SF5M90T_3453 | *yiaG* | putative transcriptional regulator | | **1.38** |  | |
| SF5M90T_2089 | *gatR* | galactitol utilization operon repressor | | **1.33** |  | |
| SF5M90T_3037 | *yqjI* | putative transcriptional regulator | | **1.30** | **-1.21** | |
| SF5M90T_71 | *cra* | transcriptional repressor of fru operon and others | | **1.16** |  | |
| SF5M90T_4197 | *yjdC* | putative transcriptional regulator | | **1.15** |  | |
| SF5M90T_1370 |  | putative regulator | | **1.09** |  | |
| SF5M90T_3452 | *cspA* | cold shock protein | | **-0.90** |  | |
| SF5M90T_2513 | *iscR* | HTH-type transcriptional regulator | | **-1.49** |  | |
| SF5M90T_3578 | *rfaH* | transcriptional activator | | **-1.57** |  | |
| SF5M90T_104 | *pdhR* | transcriptional regulator for pyruvate dehydrogenase complex | | **-1.69** | **1.82** | |
| SF5M90T_4242 | *yjeB* | HTH-type transcriptional repressor | | **-1.95** |  | |
| SF5M90T_2961 | *glcC* | transcriptional activator for *glc* operon | | **-2.06** | **1.51** | |
| SF5M90T_3839 | *lldR* | transcriptional regulator | | **-2.68** | **5.94** | |
| SF5M90T_984 | *cspH* | cold shock-like protein | | **-3.35** |  | |
| SF5M90T_1738 |  | putative transcriptional regulator LYSR-type | | **-3.44** | **2.60** | |
| SF5M90T_323 | *betI* | probably transcriptional repressor of *bet* genes | | **-4.24** | **3.58** | |
|  | | | |  |  | |
| Replication, recombination and repair | | | |  |  | |
| SF5M90T_2925 | *endA* | DNA-specific endonuclease I | | **1.42** | **-3.26** | |
| SF5M90T_3034 | *ygjF* | G/U mismatch-specific DNA glycosylase | | **1.23** |  | |
| SF5M90T_410 | *hupB* | DNA-binding protein HU-beta, NS1 (HU-1) | | **1.08** |  | |
| SF5M90T_775 | *rhlE* | putative ATP-dependent RNA helicase | | **-1.16** |  | |
| SF5M90T_3117 | *deaD* | inducible ATP-independent RNA helicase | | **-1.20** |  | |
| SF5M90T_1769 | *dbpA* | ATP-dependent RNA helicase | | **-1.86** |  | |
|  |  |  | |  |  | |
| ***Poorly characterized*** | | | |  |  | |
| General function prediction only | | | |  |  | |
| SF5M90T_4017 | *dcuB* | anaerobic dicarboxylate transport | | **6.45** | **-4.09** | |
| SF5M90T_2705 | *hypA* | probable hydrogenase nickel incorporation protein | | **4.38** | **-2.62** | |
| SF5M90T_2574 | *yfiD* | putative formate acetyltransferase | | **3.71** | **-3.14** | |
| SF5M90T_2762 | *stpA* | DNA-binding protein | | **3.51** | **-3.00** | |
| SF5M90T_4200 | *dcuA* | anaerobic dicarboxylate transport | | **3.41** | **-1.85** | |
| SF5M90T_2970 | *hybF* | probable hydrogenase nickel incorporation protein | | **3.20** | **-2.32** | |
| SF5M90T_2447 | *yffG* | putative oxidoreductase, Fe-S subunit | | **2.98** | **-2.74** | |
| SF5M90T_275 |  | putative crossover junction endodeoxyribonuclease | | **2.84** |  | |
| SF5M90T_2418 | *muQ* | N-acetylmuramic acid 6-phosphate etherase | | **2.77** |  | |
| SF5M90T_1724 |  | putative acetyltransferase | | **2.06** | **-2.41** | |
| SF5M90T_2435 |  | putative amino acid antiporter | | **2.03** | **1.90** | |
| SF5M90T_2301 | *yfbT* | putative phosphatase | | **2.00** |  | |
| SF5M90T_773 | *ybhG* | putative membrane protein | | **1.78** | **1.02** | |
| SF5M90T_1227 | *hns* | DNA-binding protein H-NS | | **1.69** | **-1.24** | |
| SF5M90T_2275 | *yfbB* | putative enzyme | | **1.62** | **-1.90** | |
| SF5M90T_2305 | *pta* | phosphotransacetylase | | **1.55** |  | |
| SF5M90T_3225 | *yrdA* | putative transferase | | **1.51** |  | |
| SF5M90T_4236 | *hfq* | RNA-binding protein Hfq | | **1.45** | **-2.10** | |
| SF5M90T_3315 | *gph* | phosphoglycolate phosphatase | | **1.25** |  | |
| SF5M90T_3111 | *yhbS* | putative acetyltransferase | | **1.19** | **-1.17** | |
| SF5M90T_751 | *ybhB* | putative kinase inhibitor protein | | **1.14** | **1.98** | |
| SF5M90T_861 | *ybjX* | putative enzyme | | **1.12** |  | |
| SF5M90T_2192 | *yeiR* | putative GTPases | | **1.03** |  | |
| SF5M90T_1919 | *yedE* | putative transport system permease protein | | **1.03** | **-1.83** | |
| SF5M90T_3651 | *ilvM* | acetolactate synthase 2 regulatory subunit | | **0.99** |  | |
| SF5M90T_3295 | *yhfC* | putative transport | | **0.97** | **-1.30** | |
| SF5M90T_2205 | *yejK* | nucleoid-associated protein | | **0.95** |  | |
| SF5M90T_2066 | *yegH* | putative transport protein | | **0.84** |  | |
| SF5M90T_3344 | *yhgH* | putative gluconate periplasmic binding protein | | **0.81** |  | |
| SF5M90T_3102 | *yraM* | putative glycosylase | | **0.73** |  | |
| SF5M90T_3118 | *yhbM* | putative control proteins | | **-1.02** |  | |
| SF5M90T_794 | *ybiP* | putative enzyme | | **-1.14** |  | |
| SF5M90T_1796 |  | putative homolog of Cu resistance protein CopC | | **-1.29** |  | |
| SF5M90T_2207 | *yejM* | putative sulfatase | | **-1.36** |  | |
| SF5M90T_3139 | *yhbE* | putative permeases of drug/metabolite transporter superfamily | | **-1.36** |  | |
| SF5M90T_966 | *yccA* | putative carrier/transport protein | | **-1.38** |  | |
| SF5M90T_4316 | *osmY* | hyperosmotically inducible periplasmic protein | | **-1.42** | **4.24** | |
| SF5M90T_190 | *yafB* | putative aldose reductase | | **-1.50** |  | |
| SF5M90T_2742 | *yqaB* | putative phosphatase | | **-1.51** |  | |
| SF5M90T_3882 | *bax* | putative ATP-binding protein | | **-1.70** |  | |
| SF5M90T_3370 | *yhhX* | putative regulator | | **-1.97** |  | |
| SF5M90T_3327 | *nudE* | ADP-ribose diphosphatase | | **-2.25** |  | |
| SF5M90T_3621 | *aslB* | putative arylsulfatase regulator | | **-2.73** |  | |
| SF5M90T_2516 |  | putative enzyme | | **-3.91** |  | |
| SF5M90T_1058 | *yceA* | predicted sulfurtransferase | | **-4.81** | **1.71** | |
|  | | | |  |  | |
| Function unknown | | | |  |  | |
| SF5M90T_1428 |  | pseudogene | | **5.24** | **-4.26** | |
| SF5M90T_4320 | *yjjI* | conserved hypothetical protein | | **4.74** | **-4.87** | |
| SF5M90T_4042 | *fdhF* | pseudogene | | **4.45** | **-2.87** | |
| SF5M90T_3166 | *yhcC* | conserved hypothetical protein | | **4.02** | **-2.57** | |
| SF5M90T_2306 | *yfcC* | conserved hypothetical protein | | **4.00** | **-5.16** | |
| SF5M90T_703 | *dcuC* | pseudogene | | **3.98** | **-5.66** | |
| SF5M90T_1511 |  | conserved hypothetical protein | | **3.93** | **-5.08** | |
| SF5M90T_3927 | *gldA* | pseudogene | | **3.73** | **-2.41** | |
| SFxv_3833 |  | conserved hypothetical protein | | **3.59** | **-4.23** | |
| SF5M90T_1155 |  | conserved hypothetical protein | | **3.44** | **-2.98** | |
| SF5M90T_1561 |  | pseudogene | | **3.25** | **-3.40** | |
| SF5M90T_3666 | *rbsB* | pseudogene | | **3.24** | **-2.31** | |
| SF5M90T_3443 | *yhiD* | pseudogene | | **3.12** | **2.39** | |
| SF5M90T_1156 | *ymgG* | conserved hypothetical protein | | **2.90** |  | |
| SF5M90T_2431 |  | conserved hypothetical protein | | **2.89** |  | |
| SF5M90T_1002 | *yccJ* | conserved hypothetical protein | | **2.79** | **2.82** | |
| SF5M90T_2432 |  | conserved hypothetical protein | | **2.69** |  | |
| SF5M90T_11 |  | uncharacterized protein | | **2.57** |  | |
| SF5M90T_2350 | *yfcZ* | conserved hypothetical protein | | **2.51** |  | |
| SF5M90T_1402 | *yeaD* | conserved hypothetical protein | | **2.45** |  | |
| SF5M90T_4300 | *yjjB* | conserved hypothetical protein | | **2.38** |  | |
| SF1275 | *yciN* | conserved hypothetical protein | | **2.29** | **-2.36** | |
| SF5M90T_828 | *ybjO* | conserved hypothetical protein | | **2.23** |  | |
| SF5M90T_1869 | *flhA* | pseudogene | | **2.16** |  | |
| SF5M90T_4032 | *phnA* | conserved hypothetical protein | | **2.14** |  | |
| SF5M90T_6 | *yaaJ* | pseudogene | | **2.05** |  | |
| SF5M90T_879 | *dmsA* | pseudogene | | **2.04** | **-2.66** | |
| SF5M90T_1941 | *dsrB* | conserved hypothetical protein | | **2.03** | **-2.43** | |
| SF5M90T_619 | *abrB* | pseudogene | | **1.99** | **-2.97** | |
| SF5M90T_3448 | *yhiU* | pseudogene | | **1.97** | **2.07** | |
| SF5M90T_2302 | *yfbU* | conserved hypothetical protein | | **1.87** |  | |
| SF5M90T_451 | *ybaK* | conserved hypothetical protein | | **1.86** | **-1.21** | |
| SSJG_00311 |  | conserved hypothetical protein | | **1.75** |  | |
| SF5M90T_2448 | *narQ* | pseudogene | | **1.64** |  | |
| SF5M90T_5 | *yaaA* | conserved hypothetical protein | | **1.54** |  | |
| SF5M90T_1387 |  | conserved hypothetical protein | | **1.51** |  | |
| SF5M90T_2165 | *yeiA* | pseudogene | | **1.47** |  | |
| SF5M90T_4067 | *yjbQ* | conserved hypothetical protein | | **1.46** |  | |
| SF5M90T_3911 | *yiiU* | conserved hypothetical protein | | **1.45** | **-2.49** | |
| SF5M90T_957 |  | conserved hypothetical protein | | **1.37** |  | |
| SF5M90T_4301 | *yjjP* | putative structural protein | | **1.33** |  | |
| SF5M90T_3012 |  | conserved hypothetical protein | | **1.33** | **-2.10** | |
| SF5M90T_4146 | *yjgD* | conserved hypothetical protein | | **1.28** | **-2.30** | |
| SF5M90T_2622 |  | conserved hypothetical protein | | **1.24** | **-1.46** | |
| SF5M90T_3375 | *ugpC* | pseudogene | | **1.19** |  | |
| SF5M90T_498 | *nfrA* | pseudogene | | **1.17** |  | |
| SF5M90T_1831 |  | pseudogene | | **1.08** |  | |
| SF5M90T_3155 | *yhbN* | conserved hypothetical protein | | **0.81** |  | |
| SF5M90T_479 | *ybbF* | conserved hypothetical protein | | **-1.19** |  | |
| SF5M90T_2991 | *ygiRQ* | pseudogene | | **-1.42** |  | |
| SF5M90T_2195 | *rtn* | conserved hypothetical protein | | **-1.50** |  | |
| SF5M90T_438 | *ybaN* | conserved hypothetical protein | | **-1.73** |  | |
| SF5M90T_2472 | *focB* | pseudogene | | **-1.87** |  | |
| SF5M90T_1853 |  | conserved hypothetical protein | | **-2.03** | **-2.58** | |
| SF5M90T_1647 |  | conserved hypothetical protein | | **-2.11** |  | |
| SF5M90T_1482 | *ydiU* | conserved hypothetical protein | | **-2.13** |  | |
| SF5M90T_147 | *yaeG* | pseudogene | | **-2.13** |  | |
| SF5M90T_58 | *yabI* | conserved hypothetical protein | | **-2.14** |  | |
| SF5M90T_1405 |  | conserved hypothetical protein | | **-2.26** | **1.90** | |
| SF5M90T_983 | *ymcD* | conserved hypothetical protein | | **-2.34** |  | |
| SF5M90T_1504 |  | pseudogene | | **-2.36** |  | |
| SF5M90T_3598 | *yigI* | conserved hypothetical protein | | **-2.48** |  | |
| SF5M90T_4094 | *yjbA* | P-starvation inducible protein PsiE | | **-2.51** | **1.69** | |
| SF5M90T_1110 | *ycfJ* | conserved hypothetical protein | | **-2.52** |  | |
| SF2861 |  | hypothetical protein; remnant | | **-2.64** |  | |
| SF5M90T_2146 | *yohO* | membrane protein | | **-2.96** |  | |
| SF5M90T_1952 |  | putative outer membrane pore protein | | **-2.98** |  | |
| SF5M90T_4307 |  | putative inner membrane protein | | **-3.40** |  | |
| SF5M90T_1683 | *yddA* | pseudogene | | **-3.58** |  | |
| SF1231 |  | conserved hypothetical protein | | **-3.71** |  | |
| SF5M90T_427 | *ybaA* | conserved hypothetical protein | | **-3.88** |  | |
| SF5M90T_2200 | *yejG* | conserved hypothetical protein | | **-4.16** |  | |
| SF5M90T_1022 | *ycdS* | pseudogene | | **-4.36** | **2.37** | |
| SF5M90T_2226 | *yojH* | pseudogene | | **-4.61** |  | |
| SF5M90T_1723 |  | conserved hypothetical protein | | **-5.28** | **2.45** | |
|  |  |  | |  |  | |
| Phage related proteins | | | | | | |
| S1668 | *relF* | prophage maintenance protein | | **1.75** |  | |
| SF5M90T_1793 |  | putative phage integrase protein | | **1.45** | **-1.60** | |
| SF5M90T_1056 |  | hypothetical bacteriophage protein | | **1.14** |  | |
| SF5M90T_740 |  | putative bacteriophage protein | | **-1.93** |  | |
|  |  |  | |  |  | |

1. Genomes used as reference are: *S. flexneri* 5a str. M90T, *S. flexneri* 2a str. 301, *S. flexneri* 2002017, *Shigella* sp. D9 and *S. flexneri* 2457T with GenBank accession numbers AGNM00000000, NC_004337, NC_017328, NZ_GG657384 and NC_004741 respectively.
2. Genes are classified in functional categories based on the database of Clusters of Orthologous Groups (COGs). <http://www.ncbi.nlm.nih.gov/COG/grace/fiew.cgi>. Inside each subgroup, genes are arranged in descending order in relation to Log2 of Fold Change values of WT no O_2_/WT O_2_ comparison.
3. Log2 of Fold Change values of WT no O_2_/WT O_2_ and Δ*fnr* no O_2_/WT no O_2_ comparisons are presented. Only values with *p* adjust <0.05 were considered differentially expressed.

Table S2. FNR regulon under anaerobic conditions

| **ORF ID^a^** | **Gene** | **Description** | **RNA-seq^b^ log2FC**  **Δ*fnr*/WT no O_2_** | | **FRT-seq^b^ log2FC Δ*fnr*/WT no O_2_** |
| --- | --- | --- | --- | --- | --- |
| SF5M90T_1 | *thrA* | aspartokinase I, homoserine dehydrogenase I | |  | **1.09** |
| SF5M90T_2 | *thrB* | homoserine kinase | |  | **0.84** |
| SF5M90T_3 | *thrC* | threonine synthase | |  | **0.82** |
| SF5M90T_6 | *yaaJ* | pseudogene | |  | **-1.44** |
| SF5M90T_10 | *yaaH* | conserved hypothetical protein | |  | **-1.10** |
| SF5M90T_15 | *nhaA* | Na+/H antiporter. pH dependent | | **1.48** | **1.49** |
| SF5M90T_16 | *nhaR* | transcriptional activator of nhaA | |  | **1.63** |
| SFxv_5153 |  | hypothetical protein | | **-1.27** |  |
| SF5M90T_29 | *caiF* | transcriptional regulator of cai operon | |  | **-1.39** |
| SF5M90T_31 | *caiD* | carnitine racemase | | **-2.04** |  |
| SF5M90T_41 | *yabF* | putative NAD(P)H oxidoreductase | | **1.53** | **1.08** |
| SF5M90T_42 | *kefC* | glutathione-regulated potassium-efflux system protein | | **1.29** | **1.39** |
| SF5M90T_43 | *folA* | dihydrofolate reductase type I | | **-1.70** | **-1.11** |
| SF5M90T_55 | *araA* | L-arabinose isomerase | | **2.22** |  |
| SF5M90T_60 | *yabK* | putative transport system permease protein | |  | **-0.76** |
| SF5M90T_63 | *yabM* | pseudogene | |  | **-0.90** |
| SF5M90T_65 | *leuC* | 3-isopropylmalate isomerase (dehydratase) subunit | |  | **1.72** |
| SF5M90T_70 | *ilvH* | acetolactate synthase III | | **1.93** |  |
| SF5M90T_74 | *yabB* | conserved hypothetical protein | | **-1.40** | **-0.82** |
| SF5M90T_75 | *yabC* | putative apolipoprotein | | **-1.14** |  |
| SF5M90T_76 | *ftsL* | cell division protein | | **-1.16** |  |
| SF5M90T_102 | *ampE* | regulatory protein | |  | **0.81** |
| SF5M90T_104 | *pdhR* | transcriptional regulator for pyruvate dehydrogenase complex | | **1.82** | **1.29** |
| SF5M90T_105 | *aceE* | pyruvate dehydrogenase (decarboxylase component) | | **2.25** | **2.41** |
| SF5M90T_106 | *aceF* | dihydrolipoamide acetyltransferase | | **2.43** | **2.55** |
| SF5M90T_109 | *acnB* | aconitate hydrase B | | **1.69** | **1.32** |
| SF5M90T_114 | *yacK* | conserved hypothetical protein | | **1.75** | **1.33** |
| SF5M90T_115 | *gcd* | glucose dehydrogenase | | **2.96** | **2.54** |
| SF5M90T_116 | *hpt* | hypoxanthine phosphoribosyltransferase | |  | **-0.93** |
| SF5M90T_117 | *yadF* | putative carbonic anhdrase | | **1.49** | **1.12** |
| SF5M90T_131 | *sfsA* | probable regulator for maltose metabolism | |  | **0.64** |
| SF5M90T_135 | *fhuA* | outer membrane protein receptor for ferrichrome | |  | **-0.82** |
| SF5M90T_139 | *hemL* | glutamate-1-semialdehyde aminotransferase (aminomutase) | |  | **-0.84** |
| SF5M90T_140 | *yadQ* | putative channel transporter | |  | **0.57** |
| SF5M90T_141 | *yadR* | conserved hypothetical protein | |  | **-0.68** |
| SF5M90T_146 | *htrA* | periplasmic serine protease | |  | **0.55** |
| SF5M90T_154 | *pyrH* | uridylate kinase | | **-1.20** | **-0.68** |
| SF5M90T_158 | *cdsA* | CDP-diglyceride synthetase | |  | **-0.51** |
| SF5M90T_164 | *lpxA* | UDP-N-acetylglucosamine acetyltransferase | |  | **-0.68** |
| SF5M90T_169 | *ldcC* | lysine decarboxylase 2 | | **2.22** | **1.55** |
| SF5M90T_170 | *yaeR* | conserved hypothetical protein | |  | **0.99** |
| SF5M90T_183 | *abc* | ATP-binding component of a transporter | |  | **-0.60** |
| SF5M90T_190 | *yafB* | putative aldose reductase | |  | **-1.76** |
| SF5M90T_193 | *yafD* | conserved hypothetical protein | |  | **0.81** |
| SF5M90T_195 | *dniR* | transcriptional regulator for nitrite reductase (cytochrome c552) | |  | **-0.51** |
| SF5M90T_211 |  | endopeptidase | |  | **-1.62** |
| SF5M90T_212 |  | putative lysozyme | |  | **-1.39** |
| SF5M90T_219 |  | putative terminase large subunit | | **-1.70** |  |
| SF5M90T_226 |  | putative packaged DNA stabilization protein | | **-3.61** |  |
| SF5M90T_246 |  | putative bacteriophage protein | | **-2.53** |  |
| SF5M90T_255 | *gtrV* | serotype-specific glucosyl transferase | | **-2.79** |  |
| SF5M90T_256 | *gtrB* | bactoprenol glucosyl transferase | | **-2.59** |  |
| SFxv_0315 |  | predicted protein | |  | **2.00** |
| SF5M90T_269 |  | putative prophage repressor CI | | **-1.60** | **-0.81** |
| SF5M90T_280 |  | putative bacteriophage protein | | **-2.57** |  |
| SF5M90T_285 | *proB* | gamma-glutamate kinase | |  | **-0.58** |
| SF5M90T_289 | *crl* | sigma factor-binding protein | |  | **1.54** |
| SF5M90T_291 | *gpt* | guanine-hypoxanthine phosphoribosyltransferase | |  | **-0.89** |
| SF5M90T_292 | *pepD* | aminoacyl-histidine dipeptidase (peptidase D) | | **1.78** | **1.75** |
| SF5M90T_297 | *yafN* | conserved hypothetical protein | | **-2.06** |  |
| SF5M90T_303 | *yafK* | conserved hypothetical protein | |  | **0.53** |
| SF5M90T_307 | *ykfE* | conserved hypothetical protein | |  | **1.67** |
| SF5M90T_308 | *yafV* | putative amidase-type enzyme | |  | **0.77** |
| SF2457T_4837 |  | hypothetical protein | |  | **0.80** |
| SF0264 |  | conserved hypothetical protein | | **-3.16** |  |
| SF5M90T_316 | *ykgE* | putative dehydrogenase subunit | | **1.26** | **1.81** |
| SF5M90T_317 | *ykgF* | uncharacterized protein with a ferredoxin-like domain | | **1.80** | **2.17** |
| SF5M90T_318 | *ykgG* | putative transporter | | **1.79** | **1.84** |
| SF5M90T_319 |  | conserved hypothetical protein | |  | **2.52** |
| SF5M90T_320 | *ykgH* | conserved hypothetical protein | |  | **1.76** |
| SF5M90T_321 | *betA* | choline dehydrogenase | | **2.87** | **2.54** |
| SF5M90T_322 | *betB* | NAD+-dependent betaine aldehyde dehydrogenase | | **3.19** | **3.36** |
| SF5M90T_323 | *betI* | probably transcriptional repressor of *bet* genes | | **3.58** | **3.50** |
| SF5M90T_324 | *betT* | high-affinity choline transport | |  | **1.74** |
| SF5M90T_331 | *tauD* | taurine dioxygenase. 2-oxoglutarate-dependent | | **1.94** |  |
| SF5M90T_337 | *yaiV* | conserved hypothetical protein | |  | **-3.51** |
| SF5M90T_344 | *yaiB* | conserved hypothetical protein | |  | **1.72** |
| SF5M90T_345 | *phoA* | alkaline phosphatase | |  | **1.68** |
| SF5M90T_346 | *psiF* | induced by phosphate starvation | |  | **2.05** |
| SF5M90T_349 | *proC* | pyrroline-5-carboxylate reductase | |  | **0.56** |
| SF5M90T_352 | *yaiA* | conserved hypothetical protein | |  | **1.45** |
| SF5M90T_358 | *yajF* | possible NAGC-like transcriptional regulator | |  | **0.70** |
| SF5M90T_364 | *brnQ* | branched chain amino acid transport system II carrier protein | |  | **-0.53** |
| SF5M90T_365 | *proY* | proline permease transport protein | |  | **-0.68** |
| SF5M90T_373 | *yajD* | conserved hypothetical protein | | **-1.74** |  |
| SF5M90T_379 | *nusB* | transcription termination L factor | |  | **-0.51** |
| SF5M90T_380 | *thiL* | thiamin-monophosphate kinase | |  | **-0.92** |
| SF5M90T_381 | *pgpA* | phosphatidylglycerophosphatase | |  | **-0.63** |
| SF5M90T_382 | *yajO* | putative NAD(P)H-dependent xylose reductase | | **1.95** | **1.74** |
| SF5M90T_386 | *yajK* | putative oxidoreductase | |  | **-0.76** |
| SF5M90T_391 |  | conserved hypothetical protein | |  | **1.58** |
| SF5M90T_395 |  | conserved hypothetical protein | |  | **0.97** |
| SF5M90T_396 |  | conserved hypothetical protein | |  | **0.90** |
| SF5M90T_404 | *ampG* | regulates beta-lactamase synthesis | |  | **-0.59** |
| SF0379 | *bolA* | possible regulator of murein genes | |  | **1.00** |
| SF5M90T_406 | *tig* | trigger factor | | **-1.02** |  |
| SF5M90T_414 | *ybaX* | conserved hypothetical protein | |  | **-0.56** |
| SF5M90T_423 | *ybaY* | glycoprotein/polysaccharide metabolism | | **2.38** | **3.36** |
| SF5M90T_434 | *acrR* | *acrAB* operon repressor | |  | **-1.01** |
| SF5M90T_439 | *apt* | adenine phosphoribosyltransferase | |  | **-0.88** |
| SF5M90T_441 | *ybaB* | conserved hypothetical protein | | **-2.57** |  |
| SF5M90T_444 | *adk* | adenylate kinase | |  | **-0.67** |
| SF5M90T_447 | *gsk* | inosine-guanosine kinase | |  | **-0.66** |
| SF5M90T_448 | *ybaL* | putative transport protein | |  | **-0.50** |
| SF5M90T_451 | *ybaK* | conserved hypothetical protein | | **-1.21** | **-1.10** |
| SF5M90T_455 | *ybaS* | putative glutaminase | | **3.98** | **3.77** |
| SF5M90T_456 | *ybaT* | putative amino acid/amine transport protein | | **3.23** | **3.34** |
| SF5M90T_457 | *ybbI* | putative transcriptional regulator | |  | **1.51** |
| SF5M90T_466 | *ybbP* | putative oxidoreductase | |  | **0.76** |
| SF5M90T_467 | *ybbB* | putative capsule anchoring protein | |  | **-0.75** |
| SF5M90T_470 | *ybbU* | putative regulator | |  | **0.93** |
| SF5M90T_481 | *cysS* | cysteine tRNA synthetase | |  | **-0.79** |
| SF5M90T_485 | *sfmA* | putative fimbrial-like protein | |  | **1.85** |
| SF5M90T_486 | *sfmC* | pseudogene | | **-3.10** |  |
| SF5M90T_498 | *nfrA* | pseudogene | |  | **-0.91** |
| SF5M90T_499 | *nfrB* | bacteriophage N4 receptor | |  | **-0.74** |
| SF5M90T_501 | *ylcA* | putative 2-component transcriptional regulator | |  | **-0.65** |
| SF5M90T_513 | *pheP* | phenylalanine-specific transport system | |  | **-0.69** |
| SF5M90T_514 | *ybdG* | putative transport | |  | **-0.79** |
| SF5M90T_518 | *ybdK* | conserved hypothetical protein | | **2.91** | **2.60** |
| SF5M90T_535 | *fepD* | ferric enterobactin (enterochelin) transport | |  | **-1.60** |
| SF5M90T_562 | *ybdS* | putative a membrane protein | |  | **-1.97** |
| SF5M90T_563 | *citG* | conserved hypothetical protein | |  | **-2.32** |
| SF5M90T_564 | *ybdU* | conserved hypothetical protein | |  | **-3.28** |
| SF5M90T_565 | *citF* | citrate lyase alpha chain | | **-2.88** | **-2.16** |
| SF5M90T_566 | *citE* | citrate lyase beta chain (acyl lyase subunit) | | **-3.43** | **-2.54** |
| SF5M90T_567 | *citD* | citrate lyase acyl carrier protein (gamma chain) | | **-3.30** |  |
| SF5M90T_568 | *citC* | pseudogene | | **-3.10** | **-1.87** |
| SF5M90T_580 | *galK* | galactokinase | |  | **0.62** |
| SF5M90T_581 | *galM* | galactose-1-epimerase | | **1.41** | **1.00** |
| SF5M90T_582 | *gpmA* | phosphoglyceromutase 1 | | **2.38** | **2.30** |
| SF5M90T_584 |  | putative homeobox protein | |  | **2.64** |
| SF5M90T_587 | *nadA* | quinolinate synthetase A protein | | **-2.08** |  |
| SF5M90T_599 | *ybgC* | conserved hypothetical protein | |  | **-0.56** |
| SF5M90T_600 | *ybgE* | conserved hypothetical protein | | **2.30** | **1.75** |
| SF5M90T_601 | *cydB* | cytochrome d terminal oxidase polypeptide subunit II | | **2.23** | **2.07** |
| SF5M90T_602 | *cydA* | cytochrome d terminal oxidase polypeptide subunit I | | **2.14** | **1.90** |
| SF5M90T_606 | *sucD* | succinyl-CoA synthetase alpha subunit | | **2.77** | **1.98** |
| SF5M90T_607 | *sucC* | succinyl-CoA synthetase beta subunit | | **1.79** | **1.88** |
| SF5M90T_608 | *sucB* | 2-oxoglutarate dehydrogenase | | **1.47** | **1.26** |
| SF5M90T_609 | *sucA* | 2-oxoglutarate dehydrogenase | | **1.62** | **1.74** |
| SF0572 |  | conserved hypothetical protein | |  | **1.93** |
| SF5M90T_610 | *sdhB* | succinate dehydrogenase iron sulfur protein | | **3.61** | **3.40** |
| SF5M90T_611 | *sdhA* | succinate dehydrogenase flavoprotein subunit | | **4.49** | **4.29** |
| SF5M90T_612 | *sdhD* | succinate dehydrogenase hydrophobic subunit | | **4.61** | **4.30** |
| SF5M90T_613 | *sdhC* | succinate dehydrogenase cytochrome b556 | | **4.54** | **4.44** |
| SF5M90T_614 | *gltA* | citrate synthase | | **2.96** | **2.73** |
| SF5M90T_619 | *abrB* | pseudogene | | **-2.97** | **-2.67** |
| SF5M90T_621 | *ybgL* | putative lactam utilization protein | |  | **1.00** |
| SF5M90T_622 | *ybgK* | pseudogene | |  | **0.98** |
| SF5M90T_623 | *ybgJ* | putative carboxylase | | **1.94** | **1.10** |
| SF5M90T_624 | *ybgI* | conserved hypothetical protein | | **1.25** | **0.95** |
| SF5M90T_625 | *ybgH* | peptide transporter | | **-1.60** | **-1.21** |
| SF5M90T_633 | *ybfA* | conserved hypothetical protein | |  | **-1.02** |
| SF5M90T_645 | *ybfE* | conserved hypothetical protein | | **-1.90** | **-1.10** |
| SF5M90T_655 | *nagD* | N-acetylglucosamine metabolism | |  | **-0.56** |
| SF5M90T_656 | *asnB* | asparagine synthetase B | |  | **0.58** |
| SF5M90T_682 | *ybeL* | putative alpha helical protein | |  | **1.44** |
| SF5M90T_689 | *ybeA* | conserved hypothetical protein | |  | **-0.53** |
| SF5M90T_699 | *ybeM* | putative amidase | |  | **0.96** |
| SF5M90T_703 | *dcuC* | pseudogene | | **-5.66** | **-3.07** |
| SF5M90T_705 |  | putative repressor protein | | **-2.06** | **-0.58** |
| SF5M90T_717 |  | putative bacteriophage protein | | **-1.64** |  |
| SF5M90T_731 |  | conserved hypothetical protein | |  | **-0.80** |
| SF0876 | *dicA* | transcriptional repressor | | **-2.66** | **-1.22** |
| SF5M90T_745 | *ybhE* | putative isomerase | | **1.44** | **1.13** |
| SF5M90T_751 | *ybhB* | putative kinase inhibitor protein | | **1.98** | **1.67** |
| SF5M90T_759 | *moaA* | molybdopterin biosynthesis protein A | | **-1.39** | **-1.42** |
| SF5M90T_760 | *moaB* | molybdopterin biosynthesis protein B | |  | **-1.19** |
| SF5M90T_761 | *moaC* | molybdopterin biosynthesis protein C | | **-0.98** | **-1.29** |
| SF5M90T_762 | *moaD* | molybdopterin biosynthesis | | **-1.18** | **-0.82** |
| SF5M90T_763 | *moaE* | molybdopterin converting factor | |  | **-0.84** |
| SF5M90T_766 | *ybhN* | conserved hypothetical protein | | **3.11** | **1.90** |
| SF5M90T_767 | *ybhO* | putative synthetase | | **2.30** | **2.84** |
| SF5M90T_768 | *ybhP* | conserved hypothetical protein | | **2.72** | **3.20** |
| SF5M90T_772 | *ybhF* | putative ATP-binding component of a transport system | |  | **0.54** |
| SF5M90T_773 | *ybhG* | putative membrane protein | | **1.02** |  |
| SF5M90T_779 | *ybiC* | putative dehydrogenase | | **2.43** | **1.81** |
| SF5M90T_785 | *ybiM* | conserved hypothetical protein | |  | **1.97** |
| SF5M90T_786 | *ybiN* | conserved hypothetical protein | |  | **-1.16** |
| SF5M90T_791 | *dps* | global regulator. Starvation conditions | | **1.98** | **2.50** |
| SF5M90T_796 | *ybiR* | conserved hypothetical protein | |  | **0.82** |
| SF5M90T_802 | *ybiY* | putative pyruvate formate-lyase 2 activating enzyme | |  | **-2.49** |
| SF5M90T_811 |  | conserved hypothetical protein | | **-1.72** | **-0.90** |
| SF5M90T_812 |  | conserved hypothetical protein | | **-2.48** | **-0.98** |
| SF5M90T_814 | *bssR* | biofilm formation regulatory protein | | **-2.00** | **-1.66** |
| SF5M90T_815 | *yliI* | pseudogene | |  | **1.57** |
| SF5M90T_816 | *yliJ* | putative transferase | |  | **0.82** |
| SF5M90T_817 | *dacC* | pseudogene | |  | **0.77** |
| SF5M90T_820 | *deoR* | transcriptional repressor | |  | **-0.55** |
| SF5M90T_821 | *ybjG* | conserved hypothetical protein | |  | **-1.36** |
| SF5M90T_822 | *cmr* | proton motive force efflux pump | |  | **-0.75** |
| SF5M90T_828 | *ybjO* | conserved hypothetical protein | |  | **-2.52** |
| SF5M90T_834 | *potF* | putrescine-binding periplasmic protein precursor | |  | **2.25** |
| SF5M90T_835 | *potG* | pseudogene | |  | **1.83** |
| SF5M90T_839 | *ybjF* | putative enzyme | |  | **-1.05** |
| SF5M90T_841 | *artM* | arginine 3rd transport system permease protein | |  | **0.57** |
| SF5M90T_842 | *artQ* | arginine 3rd transport system permease protein | |  | **0.71** |
| SF5M90T_843 | *artI* | arginine 3rd transport system periplasmic binding protein | |  | **0.87** |
| SF5M90T_845 | *ybjP* | putative enzyme | | **1.99** | **1.10** |
| SF5M90T_849 |  | conserved hypothetical protein | |  | **0.83** |
| SF5M90T_850 |  | putative regulator | |  | **0.95** |
| SF5M90T_852 | *ybjT* | putative dTDP-glucose enzyme | |  | **0.60** |
| SF5M90T_853 | *ybjU* | putative arylsulfatase | |  | **1.14** |
| SF5M90T_854 | *poxB* | pyruvate oxidase | | **3.54** | **3.22** |
| SF5M90T_860 | *ybjD* | conserved hypothetical protein | |  | **-0.72** |
| SF5M90T_861 | *ybjX* | putative enzyme | |  | **-0.67** |
| SF5M90T_863 |  | putative membrane protein | |  | **1.01** |
| SF5M90T_865 | *cspD* | cold shock protein | |  | **1.49** |
| SF5M90T_867 | *clpA* | ATP-binding component of serine protease | |  | **0.49** |
| SF5M90T_869 | *infA* | protein chain initiation factor IF-1 | |  | **-0.72** |
| SF5M90T_879 | *dmsA* | pseudogene | | **-2.66** | **-2.57** |
| SF5M90T_880 | *dmsB* | anaerobic dimethyl sulfoxide reductase subunit B | | **-3.77** |  |
| SF5M90T_882 | *ycaC* | conserved hypothetical protein | | **2.13** | **2.61** |
| SF5M90T_893 | *pflA* | pyruvate formate lyase activating enzyme 1 | |  | **-0.82** |
| SF5M90T_3080 | *pflB* | formate acetyltransferase 1 | | **-1.93** | **-2.05** |
| SF5M90T_895 | *focA* | probable formate transporter (formate channel 1) | | **-2.16** | **-2.87** |
| SF5M90T_897 | *ycaP* | pseudogene | |  | **1.61** |
| SF5M90T_898 | *serC* | 3-phosphoserine aminotransferase | |  | **0.64** |
| SF5M90T_912 | *smtA* | S-adenosylmethionine-dependentmethyltransferase | |  | **-1.02** |
| SF5M90T_913 | *mukF* | chromosome partition protein | |  | **-0.69** |
| SF5M90T_914 | *mukE* | conserved hypothetical protein | |  | **-0.58** |
| SF5M90T_917 | *ycbB* | putative amidase | | **1.17** | **0.83** |
| SF5M90T_920 | *aspC* | aspartate aminotransferase | |  | **0.82** |
| SF5M90T_921 | *ompF* | outer membrane protein | | **-2.09** | **-1.68** |
| SF5M90T_923 | *pncB* | nicotinate phosphoribosyltransferase | |  | **-0.52** |
| SF5M90T_924 | *pepN* | aminopeptidase N | |  | **0.63** |
| SF5M90T_941 | *pyrD* | dihydro-orotate dehydrogenase | | **-1.51** | **-1.61** |
| SF5M90T_962 |  | conserved hypothetical protein | |  | **1.08** |
| SF5M90T_965 | *yccK* | putative sulfite reductase | | **-1.83** |  |
| SF5M90T_966 | *yccA* | putative carrier/transport protein | |  | **-1.18** |
| SF5M90T_988 | *gnsA* | GnsA protein | |  | **-1.34** |
| SF5M90T_998 | *yccD* | conserved hypothetical protein | | **2.01** | **1.63** |
| SF5M90T_999 | *cbpA* | curved DNA-binding protein | | **1.53** | **1.32** |
| SF5M90T_1001 | *agp* | periplasmic glucose-1-phosphatase | | **1.40** | **1.57** |
| SF5M90T_1002 | *yccJ* | conserved hypothetical protein | | **2.82** | **1.85** |
| SF5M90T_1003 | *wrbA* | NAD(P)H dehydrogenase (quinone) | | **2.44** | **1.24** |
| SF5M90T_1004 | *ycdF* | conserved hypothetical protein | |  | **3.02** |
| SF5M90T_1017 | *phoH* | pseudogene | | **2.08** | **2.51** |
| SF5M90T_1022 | *ycdS* | pseudogene | | **2.37** | **2.93** |
| SF5M90T_1024 |  | Rtn-like protein | |  | **1.64** |
| SF5M90T_1029 | *ycdW* | pseudogene | |  | **0.86** |
| SF5M90T_1032 | *ycdZ* | conserved hypothetical protein | |  | **0.76** |
| SF5M90T_1043 | *ymdC* | pseudogene | |  | **0.71** |
| SF5M90T_1048 |  | conserved hypothetical protein | | **-2.38** |  |
| SF5M90T_1057 | *htrB* | heat shock protein | |  | **-0.72** |
| SF5M90T_1058 | *yceA* | predicted sulfurtransferase | | **1.71** | **1.47** |
| SF5M90T_1059 | *yceI* | conserved hypothetical protein | |  | **2.21** |
| SF5M90T_1066 | *grxB* | glutaredoxin 2 | | **2.13** | **1.01** |
| SF5M90T_1067 | *yceL* | conserved hypothetical protein | | **1.33** |  |
| SF5M90T_1087 | *yceC* | conserved hypothetical protein | | **-1.22** | **-0.95** |
| SF5M90T_1088 | *yceF* | conserved hypothetical protein | |  | **1.02** |
| SF5M90T_1090 | *plsX* | glycerolphosphate auxotrophy in plsB background | |  | **-0.60** |
| SF5M90T_1091 | *fabH* | 3-oxoacyl-[acyl-carrier-protein] synthase III | |  | **-0.64** |
| SF5M90T_1094 | *acpP* | acyl carrier protein | | **-3.04** |  |
| SF5M90T_1109 | *ndh* | respiratory NADH dehydrogenase | |  | **0.93** |
| SF5M90T_1110 | *ycfJ* | conserved hypothetical protein | |  | **0.99** |
| SF5M90T_1112 | *ycfR* | conserved hypothetical protein | | **-1.73** | **-1.40** |
| SF5M90T_1114 | *mfd* | transcription-repair coupling factor | |  | **0.44** |
| SF5M90T_1121 | *potD* | spermidine/putrescine periplasmic transport protein | |  | **-0.69** |
| SF5M90T_1122 | *potC* | spermidine/putrescine transport system permease | | **-1.09** | **-0.92** |
| SF5M90T_1123 | *potB* | spermidine/putrescine transport system permease | |  | **-1.03** |
| SF5M90T_1124 | *potA* | ATP-binding component of spermidine/putrescine transport | | **-1.47** | **-0.93** |
| SF5M90T_1125 | *pepT* | putative peptidase T | | **-2.66** | **-2.20** |
| SF5M90T_1130 |  | bacteriophage P4 DNA primase | |  | **2.88** |
| SF5M90T_1131 |  | hypothetical bacteriophage protein | |  | **1.46** |
| SF5M90T_1132 |  | hypothetical bacteriophage protein | |  | **1.64** |
| SF1144 |  | putative uncharacterized protein | | **-3.37** |  |
| SF5M90T_1151 | *icdA* | isocitrate dehydrogenase specific for NADP+ | | **1.73** | **1.84** |
| SF5M90T_1155 |  | conserved hypothetical protein | | **-2.98** | **-1.47** |
| SF5M90T_1170 | *dsbB* | disulfide bond formation protein B | |  | **-0.95** |
| SF5M90T_1173 | *ycgB* | putative sporulation protein | | **3.34** | **2.99** |
| SF5M90T_1174 | *dadA* | D-amino acid dehydrogenase subunit | | **3.40** | **3.45** |
| SF5M90T_1175 | *dadX* | alanine racemase 2 | | **1.72** | **2.45** |
| SF5M90T_1177 |  | conserved hypothetical protein | |  | **0.69** |
| SF5M90T_1179 | *mltE* | murein transglycosylase E | |  | **-0.52** |
| SF5M90T_1181 | *ymgE* | conserved hypothetical protein | |  | **2.78** |
| SF5M90T_1189 | *treA* | trehalase periplasmic | | **2.42** | **2.25** |
| SF5M90T_1190 | *ycgC* | putative PTS system enzyme I | | **1.83** | **1.20** |
| SF5M90T_1191 |  | dihydroxyacetone kinase subunit DhaL | | **1.92** | **1.40** |
| SF5M90T_1192 |  | dihydroxyacetone kinase subunit DhaK | | **2.03** | **1.30** |
| SF5M90T_1197 | *ychH* | conserved hypothetical protein | |  | **1.53** |
| SF5M90T_1210 | *chaB* | cation transport regulator | |  | **1.45** |
| SF5M90T_1211 | *chaC* | cation transport regulator | |  | **1.04** |
| SF5M90T_1216 | *narK* | nitrite extrusion protein | | **-2.52** | **-3.13** |
| SF5M90T_1217 | *narG* | nitrate reductase 1 alpha subunit | | **-2.63** | **-1.59** |
| SF1231 |  | hypothetical protein | |  | **-1.60** |
| SF5M90T_1224 | *ychK* | conserved hypothetical protein | |  | **1.19** |
| SF5M90T_1225 | *hnr* | regulator of RpoS | |  | **1.56** |
| SF5M90T_1227 | *hns* | DNA-binding protein H-NS | | **-1.24** |  |
| SF5M90T_1231 | *oppA* | periplasmic oligopeptide-binding protein precursor | | **1.25** | **0.95** |
| SF5M90T_1233 | *oppC* | oligopeptide transport permease protein | |  | **0.83** |
| SF5M90T_1234 | *oppD* | ATP-binding protein of oligopeptide ABC transport system | | **1.21** | **0.93** |
| SF5M90T_1235 | *oppF* | ATP-binding protein of oligopeptide ABC transport system | |  | **0.97** |
| SF5M90T_1242 | *yciA* | conserved hypothetical protein | |  | **-1.06** |
| SF5M90T_1243 | *yciB* | probable intracellular septation protein A | |  | **-0.80** |
| SF5M90T_1247 | *yciF* | putative structural proteins | |  | **3.13** |
| SF5M90T_1248 | *yciG* | conserved hypothetical protein | |  | **3.52** |
| SF5M90T_1249 | *trpA* | tryptophan synthase alpha protein | | **1.52** |  |
| SF5M90T_1250 | *trpB* | tryptophan synthase beta protein | |  | **0.92** |
| SF5M90T_1251 | *trpC* | bifunctional indole-3-glycerol phosphate synthase | |  | **1.05** |
| SF5M90T_1256 | *yciL* | conserved hypothetical protein | |  | **-0.97** |
| SF1275 | *yciN* | conserved hypothetical protein | | **-2.36** | **-0.78** |
| SF5M90T_1264 | *acnA* | aconitate hydrase 1 | | **2.83** | **2.66** |
| SF5M90T_1269 | *pyrF* | orotidine-5'-phosphate decarboxylase | | **-0.84** | **-1.13** |
| SF5M90T_1270 | *yciH* | conserved hypothetical protein | |  | **-1.00** |
| SF5M90T_1274 | *yciR* | pseudogene | |  | **1.01** |
| SF5M90T_1276 | *yciW* | putative oxidoreductase | |  | **-0.62** |
| SF5M90T_1283 | *sapA* | peptide transport periplasmic protein | |  | **-0.76** |
| SF5M90T_1290 | *ordL* | probable oxidoreductase | | **2.09** |  |
| SF5M90T_1309 | *ompG* | pseudogene | | **-3.03** |  |
| SF5M90T_1311 | *ycjX* | putative EC 2.1 enzymes | |  | **0.65** |
| SF5M90T_1312 | *ycjF* | conserved hypothetical protein | |  | **0.82** |
| SF5M90T_1314 | *tpx* | thiol peroxidase | | **2.29** | **1.90** |
| SF5M90T_1319 |  | conserved hypothetical protein | |  | **1.19** |
| SF5M90T_1320 |  | conserved hypothetical protein | | **1.62** | **1.79** |
| SF5M90T_1330 |  | putative bacteriophage protein | |  | **1.61** |
| SF5M90T_1360 |  | conserved hypothetical protein | | **3.22** | **2.86** |
| SF5M90T_1362 |  | conserved hypothetical protein | |  | **1.72** |
| SF5M90T_1363 |  | putative paraquat-inducible protein | | **1.91** | **1.72** |
| SF5M90T_1365 |  | conserved hypothetical protein | |  | **1.42** |
| SF5M90T_1371 |  | conserved hypothetical protein | | **-3.32** | **-0.97** |
| SF5M90T_1372 |  | conserved hypothetical protein | | **-1.66** | **-0.88** |
| SF1402 |  | conserved hypothetical protein | | **-1.23** |  |
| SF5M90T_1373 | *cspC* | cold shock protein | | **-1.38** |  |
| SF5M90T_1375 |  | conserved hypothetical protein | |  | **-0.84** |
| SF5M90T_1389 | *yeaZ* | conserved hypothetical protein | | **-0.93** |  |
| SF5M90T_1392 | *rnd* | RNase D | |  | **-0.67** |
| SF5M90T_1404 | *yeaA* | conserved hypothetical protein | |  | **1.88** |
| SF5M90T_1405 |  | conserved hypothetical protein | | **1.90** | **1.35** |
| SF5M90T_1419 | *ydjA* | predicted oxidoreductase | | **1.69** | **1.52** |
| SF5M90T_1421 | *topB* | DNA topoisomerase III | |  | **-0.44** |
| SF5M90T_1424 | *gdhA* | NADP-specific glutamate dehydrogenase | |  | **0.76** |
| SF5M90T_1428 |  | pseudogene | | **-4.26** | **-4.24** |
| SF5M90T_1435 | *ydjY* | conserved hypothetical protein | |  | **-2.94** |
| SF5M90T_1436 | *ydjX* | conserved hypothetical protein | | **-2.22** | **-2.23** |
| SF5M90T_1438 | *cstC* | pseudogene | | **1.92** | **2.18** |
| SF5M90T_1442 | *ydjS* | conserved hypothetical protein | |  | **2.06** |
| SF5M90T_1443 | *spy* | periplasmic protein | | **1.71** | **1.96** |
| SF5M90T_1447 | *osmE* | activator of ntrL gene | |  | **2.08** |
| SF5M90T_1454 | *katE* | catalase | | **4.18** | **3.99** |
| SF5M90T_1457 |  | pseudogene | | **-1.76** | **-2.34** |
| SF5M90T_1458 |  | conserved hypothetical protein | |  | **-1.71** |
| SF5M90T_1461 |  | conserved hypothetical protein | | **1.84** | **1.59** |
| SF5M90T_1462 |  | conserved hypothetical protein | |  | **2.43** |
| SF5M90T_1463 | *pfkB* | 6-phosphofructokinase II | | **1.62** | **1.76** |
| SF5M90T_1470 | *infC* | protein chain initiation factor IF-3 | | **-1.18** |  |
| SF5M90T_1478 | *btuE* | vitamin B12 transport | |  | **0.93** |
| SF5M90T_1479 | *btuD* | ATP-binding component of vitamin B12 transport system | |  | **0.58** |
| SF5M90T_1483 | *ydiE* | hemin uptake protein | | **-2.20** | **-1.55** |
| SF5M90T_1485 | *ydiA* | conserved hypothetical protein | | **1.33** | **0.98** |
| SF5M90T_1486 | *ppsA* | phosphoenolpyruvate synthase | |  | **0.85** |
| SF5M90T_1495 |  | putative ARAC-type regulatory protein | |  | **2.31** |
| SF5M90T_1496 |  | putative oxidoreductase major subunit | | **3.09** | **3.12** |
| SF5M90T_1511 |  | conserved hypothetical protein | | **-5.08** | **-5.23** |
| SF5M90T_1514 | *dcp* | dipeptidyl carboxypeptidase II | |  | **0.63** |
| SF5M90T_1515 | *ydeJ* | conserved hypothetical protein | |  | **2.00** |
| SF5M90T_1516 | *ydeI* | conserved hypothetical protein | |  | **2.14** |
| SF5M90T_1519 |  | putative oxidoreductase major subunit | | **-4.97** | **-3.30** |
| SF5M90T_1520 |  | conserved hypothetical protein | | **2.24** | **1.66** |
| SF5M90T_1523 |  | conserved hypothetical protein | | **-1.46** |  |
| SF5M90T_1529 |  | putative ATP-binding component of a transport system | | **1.99** |  |
| SF5M90T_1560 |  | putative oxidoreductase major subunit | |  | **-3.87** |
| SF5M90T_1561 |  | pseudogene | | **-3.40** | **-2.62** |
| SF5M90T_1562 |  | putative oxidoreductase Fe-S subunit | | **-1.98** | **-1.50** |
| SF5M90T_1563 |  | putative DMSO reductase anchor subunit | |  | **-1.24** |
| SF5M90T_1566 | *bioD* | ATP-dependent dethiobiotin synthetase | | **-3.78** | **-3.71** |
| SF5M90T_1568 | *ynfL* | putative transcriptional regulator LYSR-type | |  | **0.67** |
| SF5M90T_1569 | *ynfM* | pseudogene | | **2.44** | **1.15** |
| SF5M90T_1571 |  | conserved hypothetical protein | |  | **1.09** |
| SF5M90T_1578 |  | putative arginine/ornithine antiporter | | **-0.93** | **-1.30** |
| SF5M90T_1581 | *rstA* | response transcriptional regulatory protein | |  | **-0.64** |
| SF5M90T_1582 |  | putative transporter | |  | **-1.25** |
| SF5M90T_1585 | *fumC* | fumarate hydratase | | **2.55** | **1.85** |
| SF5M90T_1586 | *fumA* | Fumarate hydratase class I | | **2.22** | **2.30** |
| SF5M90T_1588 | *ydgA* | conserved hypothetical protein | |  | **0.83** |
| SF5M90T_1600 |  | conserved hypothetical protein | | **-1.95** | **-2.25** |
| SF5M90T_1601 |  | conserved hypothetical protein | | **-1.26** | **-1.45** |
| SF5M90T_1604 | *rnfC* | electron transport complex protein | |  | **-0.62** |
| SF5M90T_1605 | *ydgO* | conserved hypothetical protein | |  | **-0.86** |
| SF5M90T_1607 | *ydgQ* | conserved hypothetical protein | |  | **-0.96** |
| SF5M90T_1608 | *nth* | endonuclease III | | **-1.70** | **-0.99** |
| SF5M90T_1609 | *ydgR* | putative transport protein | |  | **-1.14** |
| SF5M90T_1618 | *slyB* | putative outer membrane protein | | **-1.43** | **-0.92** |
| SF5M90T_1619 | *slyA* | transcriptional regulator for cryptic hemolysin | | **-1.43** |  |
| SF5M90T_1625 |  | conserved hypothetical protein | |  | **0.98** |
| SF5M90T_1635 | *ydhO* | putative lipoprotein | |  | **-0.78** |
| SF5M90T_1636 | *sodB* | superoxide dismutase | | **2.13** | **1.67** |
| SF5M90T_1637 |  | putative transport protein | | **1.58** |  |
| SF5M90T_1640 | *ydhC* | putative transport protein | |  | **0.69** |
| SF5M90T_1644 |  | possible enzyme | |  | **1.32** |
| SF5M90T_1648 |  | conserved hypothetical protein | | **2.12** | **2.75** |
| SF5M90T_1654 |  | putative oxidoreductase. Fe-S subunit | |  | **-2.14** |
| SF5M90T_1659 | *ynhG* | conserved hypothetical protein | | **1.86** | **1.97** |
| SF5M90T_1660 | *sufE* | cysteine desulfuration protein | | **1.42** | **1.83** |
| SF5M90T_1661 | *sufS* | cysteine desulfurase | | **2.52** | **2.35** |
| SF5M90T_1662 | *sufD* | cysteine desulfurase activator complex subunit | | **3.01** | **2.36** |
| SF5M90T_1663 | *sufC* | cysteine desulfurase ATPase component | | **2.73** | **2.73** |
| SF5M90T_1664 | *sufB* | cysteine desulfurase activator complex subunit | | **3.00** | **2.55** |
| SF5M90T_1665 | *sufA* | Iron-sulfur cluster assembly scaffold protein | | **2.24** | **2.70** |
| SF5M90T_1668 | *ydiJ* | putative oxidase | | **1.34** | **0.74** |
| SF5M90T_1683 | *yddA* | pseudogene | |  | **-1.53** |
| SF5M90T_1689 | *gadB* | glutamate decarboxylase isozyme | | **5.19** | **5.33** |
| SF5M90T_1690 | *xasA* | acid sensitivity protein, putative transporter | | **5.30** | **5.20** |
| SF5M90T_1691 |  | conserved hypothetical protein | |  | **-0.59** |
| SF5M90T_1692 |  | conserved hypothetical protein | |  | **1.45** |
| SF5M90T_1698 | *osmC* | osmotically inducible protein | | **2.28** | **2.21** |
| SF5M90T_1701 | *sfcA* | NAD-linked malate dehydrogenase | | **1.46** | **1.60** |
| SF5M90T_1702 | *adhP* | pseudogene | | **3.44** | **3.24** |
| SF5M90T_1703 | *yddM* | conserved hypothetical protein | | **-1.53** |  |
| SF5M90T_1704 | *fdnI* | formate dehydrogenase-N gamma subunit | | **-3.54** | **-3.40** |
| SF5M90T_1705 | *fdnH* | formate dehydrogenase-N beta subunit | | **-4.23** | **-4.89** |
| SF5M90T_1706 | *fdnG* | formate dehydrogenase-N alpha subunit | | **-5.67** | **-5.70** |
| SF5M90T_1713 | *narU* | nitrite extrusion protein 2 | | **4.90** | **2.73** |
| SF5M90T_1217 | *narZ* | cryptic nitrate reductase 2 alpha subunit | | **2.16** | **1.91** |
| SF5M90T_1715 | *narY* | putative cryptic nitrate reductase 2 beta subunit | |  | **2.25** |
| SF5M90T_1718 |  | conserved hypothetical protein | |  | **1.50** |
| SF5M90T_1719 | *yncB* | putative oxidoreductase | | **1.94** | **1.65** |
| SF5M90T_1722 |  | conserved hypothetical protein | | **-1.62** |  |
| SF5M90T_1723 |  | conserved hypothetical protein | | **2.45** |  |
| SF5M90T_1724 |  | putative acetyltransferase | | **-2.41** |  |
| SF5M90T_1730 |  | conserved hypothetical protein | |  | **1.59** |
| SF5M90T_1731 | *tehB* | tellurite resistance | | **1.41** | **2.91** |
| SF5M90T_1732 | *tehA* | tellurite resistance | | **2.33** | **2.48** |
| SF5M90T_1733 |  | conserved hypothetical protein | | **2.03** | **2.70** |
| SF5M90T_1734 | *rimL* | ribosomal-protein-serine acetyltransferase | | **-2.42** |  |
| SF5M90T_1735 | *ydcH* | conserved hypothetical protein | | **-2.76** |  |
| SF5M90T_1737 |  | conserved hypothetical protein | | **1.96** | **1.90** |
| SF5M90T_1738 |  | putative transcriptional regulator LYSR-type | | **2.60** | **2.58** |
| SF5M90T_1743 | *cybB* | cytochrome b(561) | |  | **1.33** |
| SF5M90T_1744 | *gapC* | glyceraldehyde-3-phosphate dehydrogenase | | **2.62** | **2.11** |
| SF5M90T_1749 | *acpD* | acyl carrier protein phosphodiesterase | |  | **1.04** |
| SF5M90T_1772 |  | conserved hypothetical protein | |  | **2.21** |
| SF5M90T_1776 |  | putative secretion protein | | **-3.26** |  |
| SF5M90T_1779 | *fnr* | fumarate and nitrate reduction regulatory protein | | **-3.51** | **-2.54** |
| SF5M90T_1781 | *ynaJ* | conserved hypothetical protein | |  | **0.97** |
| SF5M90T_1793 |  | putative phage integrase protein | | **-1.60** |  |
| SF5M90T_1795 |  | putative resistance protein | | **1.47** | **0.82** |
| SF5M90T_1796 |  | putative homolog of Cu resistance protein CopC | |  | **0.67** |
| SF5M90T_1800 | *ptrB* | protease II | |  | **1.37** |
| SF5M90T_1803 | *yebG* | pseudogene | | **-1.41** |  |
| SF5M90T_1806 | *edd* | 6-phosphogluconate dehydratase | |  | **-0.72** |
| SF5M90T_1809 | *pykA* | pyruvate kinase | | **-1.57** | **-1.76** |
| SF5M90T_1822 | *yecD* | pseudogene | |  | **1.03** |
| SF5M90T_1846 |  | putative bacteriophage protein | | **4.10** |  |
| SF5M90T_1850 |  | hypothetical bacteriophage protein | | **-2.27** |  |
| SF5M90T_1853 |  | conserved hypothetical protein | | **-2.58** |  |
| SF5M90T_1854 |  | conserved hypothetical protein | | **-1.92** |  |
| SF5M90T_1887 | *otsA* | pseudogene | | **1.99** | **2.25** |
| SF5M90T_1888 | *otsB* | trehalose-6-phosphate phophatase | | **3.69** | **3.49** |
| SF5M90T_1890 | *araG* | L-arabinose transport ATP-binding protein | |  | **1.96** |
| SF5M90T_1893 |  | conserved hypothetical protein | |  | **-1.00** |
| SF5M90T_1896 | *yecH* | conserved hypothetical protein | |  | **-3.01** |
| SF5M90T_1897 | *tyrP* | tyrosine-specific transport system | |  | **-1.12** |
| SF5M90T_1905 | *yecF* | conserved hypothetical protein | | **-4.33** | **-1.24** |
| SF5M90T_1917 | *amyA* | cytoplasmic alpha-amylase | | **2.98** | **3.29** |
| SF5M90T_1919 | *yedE* | putative transport system permease protein | | **-1.83** | **-1.96** |
| SF5M90T_1920 | *yedF* | conserved hypothetical protein | |  | **-1.09** |
| SF5M90T_1921 | *yedK* | pseudogene | | **1.54** | **1.49** |
| SF5M90T_1923 | *nmpC* | outer membrane porin protein | | **-1.34** | **-1.13** |
| SF5M90T_1941 | *dsrB* | conserved hypothetical protein | | **-2.43** |  |
| SF5M90T_1942 |  | conserved hypothetical protein | | **2.86** | **2.74** |
| SF5M90T_1943 |  | pseudogene | |  | **1.82** |
| SF5M90T_1944 |  | predicted diguanylate cyclase | |  | **1.10** |
| SF2001 |  | conserved hypothetical protein | |  | **2.06** |
| SF5M90T_1963 |  | conserved hypothetical protein | | **-2.18** |  |
| SF5M90T_1965 |  | unknown protein encoded within prophage | | **-2.43** |  |
| SF5M90T_1506 |  | conserved hypothetical protein | | **-3.55** |  |
| SF5M90T_1507 | *relB* | negative regulator of translation | | **-2.13** |  |
| SF5M90T_1997 | *amn* | AMP nucleosidase | |  | **1.04** |
| SF5M90T_1998 |  | conserved hypothetical protein | | **-2.09** |  |
| SF5M90T_2003 | *cbl* | transcriptional regulator cys regulon | |  | **-1.50** |
| SF5M90T_2015 | *yeeD* | conserved hypothetical protein | |  | **-1.41** |
| SF5M90T_2016 | *yeeE* | putative transport system permease protein | | **-1.91** | **-1.95** |
| SF5M90T_2017 | *yeeF* | putative amino acid/amine transport protein | | **-2.09** | **-2.44** |
| SF5M90T_2028 | *hisA* | 1-(5-phosphoribosyl)-5-[(5-phosphoribosylamino)methylideneamino] imidazole-4-carboxamide isomerase | |  | **0.67** |
| SF2092 |  | hypothetical protein | | **-1.33** |  |
| SF2094 |  | putative uncharacterized protein | | **-2.65** |  |
| SF2095 | *rfbJ* | protein Rfbj | | **-1.75** |  |
| SF5M90T_2035 | *rfc* | O-antigen polymerase | | **-1.76** |  |
| SF5M90T_2036 | *rfbG* | dTDP-rhamnosyl transferase | | **-3.02** |  |
| SF5M90T_2037 | *rfbF* | dTDP-rhamnosyl transferase | | **-2.54** |  |
| SF5M90T_2038 | *rfbE* | polysaccharide biosynthesis protein | | **-1.53** |  |
| SF5M90T_2039 | *rfbC* | dTDP-4-dehydrorhamnose 3.5-epimerase | | **-2.64** |  |
| SF5M90T_2040 | *rfbA* | glucose-1-phosphate thymidylyltransferase | | **-1.28** |  |
| SF5M90T_2068 | *dcd* | 2'-deoxycytidine 5'-triphosphate deaminase | |  | **-0.63** |
| SF5M90T_2074 | *yegD* | putative heat shock protein | |  | **-1.11** |
| SF5M90T_2082 | *yegB* | putative transport protein | |  | **1.85** |
| SF5M90T_2085 |  | conserved hypothetical protein | |  | **3.47** |
| SF5M90T_2086 | *yegQ* | conserved hypothetical protein | | **-0.89** | **-0.83** |
| SFxv_2376a |  | conserved hypothetical protein | |  | **2.68** |
| SF5M90T_2087 |  | conserved hypothetical protein | |  | **2.77** |
| SF5M90T_2088 |  | conserved hypothetical protein | | **3.48** | **3.13** |
| SF5M90T_2096 |  | fructose-bisphosphate aldolase | | **2.08** | **1.86** |
| SF5M90T_2104 | *yohL* | conserved hypothetical protein | |  | **1.26** |
| SF5M90T_2105 | *yohM* | conserved hypothetical protein | | **-2.17** | **-2.75** |
| SF5M90T_2106 |  | conserved hypothetical protein | |  | **-0.71** |
| SF5M90T_2107 | *yehA* | putative type-1 fimbrial protein | | **-4.23** |  |
| SF5M90T_2112 | *yehD* | putative fimbrial-like protein | | **-3.05** | **-3.01** |
| SF5M90T_2147 | *yehW* | putative transport system permease protein | | **1.89** | **2.26** |
| SF5M90T_2148 | *yehX* | putative ATP-binding component of a transport system | | **2.98** | **2.71** |
| SF5M90T_2149 | *yehY* | putative transport system permease protein | | **2.91** | **2.27** |
| SF5M90T_2150 | *yehZ* | putative transport system permease protein | | **1.98** | **1.83** |
| SF5M90T_2152 | *dld* | D-lactate dehydrogenase | | **2.58** | **2.21** |
| SF5M90T_2156 | *yohF* | pseudogene | |  | **2.20** |
| SF5M90T_2158 | *yohI* | putative regulator protein | |  | **-1.00** |
| SF5M90T_2161 | *cdd* | cytidine/deoxycytidine deaminase | |  | **0.75** |
| SF5M90T_2166 | *mglC* | methyl-galactoside transport and galactose taxis | | **3.12** | **1.66** |
| SF5M90T_2167 | *mglA* | galactoside transport ATP-binding protein | | **1.95** | **2.39** |
| SF5M90T_2168 | *mglB* | D-galactose-binding periplasmic protein precursor | | **1.86** | **2.37** |
| SF5M90T_2170 | *yeiB* | conserved hypothetical protein | |  | **0.65** |
| SF5M90T_2171 | *folE* | GTP cyclohydrolase I | |  | **0.69** |
| SF5M90T_2172 | *yeiG* | putative esterase | | **2.09** | **1.40** |
| SF5M90T_2174 | *lysP* | lysine-specific permease | |  | **0.65** |
| SF5M90T_2176 | *yeiH* | conserved hypothetical protein | |  | **-1.06** |
| SF5M90T_2191 | *yeiQ* | putative oxidoreductase | | **-1.54** |  |
| SF5M90T_2193 |  | conserved hypothetical protein | | **-1.33** | **-1.41** |
| SF5M90T_2199 | *yejF* | putative ATP-binding component of a transport system | | **1.30** |  |
| SF5M90T_2206 | *yejL* | conserved hypothetical protein | | **-1.42** |  |
| SF5M90T_2210 | *narP* | nitrate/nitrite response regulator | |  | **-0.88** |
| SF5M90T_2211 | *ccmH* | possible subunit of heme lyase | | **-2.13** | **-2.32** |
| SF5M90T_2212 | *dsbE* | Thiol:disulfide interchange protein | | **-2.09** | **-2.36** |
| SF5M90T_2213 | *ccmF* | cytochrome c-type biogenesis protein | | **-2.11** | **-2.43** |
| SF5M90T_2214 | *ccmE* | cytochrome c-type biogenesis protein | | **-2.44** | **-2.74** |
| SF5M90T_2215 | *ccmD* | heme exporter protein C | | **-2.78** | **-2.31** |
| SF5M90T_2216 | *ccmC* | heme exporter protein C | | **-2.85** | **-2.96** |
| SF5M90T_2217 | *ccmB* | heme exporter protein B cytochrome c-type biogenesis protein | | **-2.29** | **-3.11** |
| SF5M90T_2218 | *ccmA* | ATP binding protein of heme exporter A | | **-3.35** | **-3.40** |
| SF5M90T_2219 | *napC* | cytochrome c-type protein | | **-4.09** | **-3.61** |
| SF5M90T_2220 | *napB* | cytochrome c-type protein | | **-3.89** | **-4.27** |
| SF5M90T_2221 | *napH* | ferredoxin-type protein: electron transfer | | **-4.62** | **-4.52** |
| SF5M90T_2222 | *napG* | ferredoxin-type protein: electron transfer | | **-4.47** | **-4.44** |
| SF5M90T_2223 | *napA* | probable nitrate reductase 3 | | **-4.85** | **-4.69** |
| SF5M90T_2224 | *napD* | assembly protein for periplasmic nitrate reductase | | **-4.52** | **-4.81** |
| SF2292 | *napF* | ferredoxin-type protein | | **-4.73** | **-5.40** |
| SF5M90T_2225 | *eco* | serine protease inhibitor | | **-1.03** |  |
| SF5M90T_2226 | *yojH* | pseudogene | |  | **2.31** |
| SF5M90T_2234 | *rcsC* | sensor protein | |  | **0.62** |
| SF5M90T_2248 | *nrdA* | ribonucleoside diphosphate reductase 1, alpha subunit. B1 | | **2.35** | **1.96** |
| SF5M90T_2249 | *nrdB* | ribonucleoside-diphosphate reductase 1, beta subunit. B2 | |  | **1.20** |
| SF5M90T_2250 | *yfaE* | conserved hypothetical protein | |  | **1.22** |
| SF5M90T_2251 | *inaA* | pH-inducible protein involved in stress response | |  | **1.16** |
| SF5M90T_2254 | *glpA* | sn-glycerol-3-phosphate dehydrogenase large subunit | | **-1.09** | **-1.49** |
| SF5M90T_2255 | *glpB* | sn-glycerol-3-phosphate dehydrogenase subunit B | | **-2.51** |  |
| SF5M90T_2256 | *glpC* | sn-glycerol-3-phosphate dehydrogenase K-small subunit | |  | **-1.81** |
| SF5M90T_2258 |  | conserved hypothetical protein | | **2.41** |  |
| SF5M90T_2261 |  | conserved hypothetical protein | |  | **-1.42** |
| SF5M90T_2265 |  | putative sugar transferase | | **2.14** |  |
| SF5M90T_2266 |  | putative transformylase | | **1.69** | **1.69** |
| SF5M90T_2267 |  | conserved hypothetical protein | |  | **2.08** |
| SF5M90T_2268 |  | conserved hypothetical protein | |  | **1.09** |
| SF5M90T_2269 |  | sucrose-6 phosphate hydrolase | |  | **1.46** |
| SF5M90T_2271 | *pmrD* | pseudogene | | **-2.14** | **-1.31** |
| SF5M90T_2272 | *menE* | o-succinylbenzoate-CoA ligase | | **-1.54** | **-1.80** |
| SF5M90T_2273 | *menC* | O-succinylbenzoate synthase | | **-1.72** | **-1.57** |
| SF5M90T_2274 | *menB* | dihydroxynaphtoic acid synthetase | | **-1.75** | **-1.65** |
| SF5M90T_2275 | *yfbB* | putative enzyme | | **-1.90** | **-1.85** |
| SF5M90T_2276 | *menD* | 2-oxoglutarate decarboxylase | | **-1.63** | **-1.75** |
| SF5M90T_2278 | *elaB* | conserved hypothetical protein | | **1.99** | **2.93** |
| SF5M90T_2284 | *nuoN* | NADH dehydrogenase I chain N | | **1.55** | **0.82** |
| SF5M90T_2285 | *nuoM* | NADH dehydrogenase I chain M | |  | **0.70** |
| SF5M90T_2286 | *nuoL* | NADH dehydrogenase I chain L | |  | **0.66** |
| SF5M90T_2287 | *nuoK* | NADH dehydrogenase I chain K | |  | **0.88** |
| SF5M90T_2288 | *nuoJ* | NADH dehydrogenase I chain J | |  | **0.87** |
| SF5M90T_2289 | *nuoI* | NADH dehydrogenase I chain I | |  | **0.87** |
| SF5M90T_2290 | *nuoH* | NADH dehydrogenase I chain H | |  | **0.84** |
| SF5M90T_2291 | *nuoG* | NADH dehydrogenase I chain G | |  | **0.82** |
| SF5M90T_2292 | *nuoF* | NADH dehydrogenase I chain F | |  | **0.80** |
| SF5M90T_2293 | *nuoE* | NADH dehydrogenase I chain E | |  | **0.74** |
| SF5M90T_2294 | *nuoC* | NADH dehydrogenase I chain C, D | |  | **0.61** |
| SF5M90T_2295 | *nuoB* | NADH dehydrogenase I chain B | |  | **0.55** |
| SSJG_00311 |  | conserved hypothetical protein | |  | **-1.60** |
| SF5M90T_2298 |  | putative aminotransferase | |  | **-0.57** |
| SF5M90T_2304 | *ackA* | acetate kinase | | **-1.33** | **-1.38** |
| SF5M90T_2305 | *pta* | phosphotransacetylase | |  | **-0.92** |
| SF5M90T_2306 | *yfcC* | conserved hypothetical protein | | **-5.16** | **-5.48** |
| SF5M90T_2307 |  | putative regulator | |  | **-0.54** |
| SF5M90T_2309 | *yfcF* | conserved hypothetical protein | |  | **2.23** |
| SF5M90T_2310 | *yfcG* | putative S-transferase | | **2.32** | **2.66** |
| SF5M90T_2311 | *folX* | D-erythro-7.8-dihydroneopterin tri P epimerase | |  | **1.65** |
| SF5M90T_2312 |  | putative sugar nucleotide epimerase | | **1.41** | **0.52** |
| SF5M90T_2330 |  | putative transport protein | |  | **-1.11** |
| SF5M90T_2331 | *fabB* | 3-oxoacyl-[acyl-carrier-protein] synthase I | |  | **-1.03** |
| SF5M90T_2332 |  | putative peptidase | |  | **-0.92** |
| SF5M90T_2338 | *yfcB* | putative adenine-specific methylase | |  | **-0.57** |
| SF5M90T_2350 | *yfcZ* | conserved hypothetical protein | |  | **-1.21** |
| SF5M90T_2351 | *fadL* | long-chain fatty acid transport protein precursor | | **-1.87** | **-1.52** |
| SF5M90T_2354 | *yfdC* | putative transport | |  | **1.37** |
| SF5M90T_2359 |  | beta-fructosidase | |  | **0.72** |
| SF5M90T_2361 |  | D-serine permease | | **-2.87** |  |
| SF5M90T_2363 | *emrY* | multidrug resistance protein Y | | **2.49** | **1.60** |
| SF5M90T_2364 | *emrK* | multidrug resistance protein K | |  | **3.79** |
| SF5M90T_2366 | *evgS* | putative sensor for regulator EvgA | |  | **1.40** |
| SF5M90T_2368 |  | putative receptor protein | |  | **2.37** |
| SF5M90T_2369 |  | putative enzyme | | **3.62** | **2.50** |
| SF5M90T_2370 |  | putative enzyme | | **3.99** | **4.01** |
| SF5M90T_2371 |  | conserved hypothetical protein | | **4.18** | **3.13** |
| SF5M90T_2373 | *ddg* | putative heat shock protein | | **1.52** | **1.11** |
| SF5M90T_2386 | *mntH* | divalent metal cation transporter | | **1.61** | **1.68** |
| SF5M90T_2388 | *yfeA* | predicted diguanylate cyclase | |  | **-0.81** |
| SF5M90T_2403 | *cysK* | cysteine synthase A | | **-1.15** | **-1.06** |
| SF5M90T_2411 | *cysM* | cysteine synthase B. O-acetylserine sulfhydrolase B | | **-1.81** | **-1.74** |
| SF5M90T_2412 | *cysA* | ATP-binding component of sulfate permease A protein | | **-1.28** | **-1.66** |
| SF5M90T_2413 | *cysW* | ABC-type sulfate transport system permease component | | **-1.28** | **-1.67** |
| SF5M90T_2414 | *cysU* | sulphate, thiosulfate transport system permease T protein | | **-1.18** | **-1.59** |
| SF5M90T_2415 | *cysP* | thiosulfate binding protein | | **-1.51** | **-1.88** |
| SF5M90T_2416 | *ucpA* | putative oxidoreductase | |  | **-0.56** |
| SF5M90T_2418 | *muQ* | N-acetylmuramic acid 6-phosphate etherase | |  | **-0.93** |
| SF5M90T_2419 | *murP* | PTS system N-acetylmuramic acid transporter subunits EIIBC | |  | **-0.71** |
| SF5M90T_2420 | *yfeX* | deferrochelatase/peroxidase | | **-0.92** | **-1.30** |
| SF5M90T_2435 |  | putative amino acid antiporter | | **1.90** | **1.58** |
| SF5M90T_2436 |  | conserved hypothetical protein | |  | **1.82** |
| SF5M90T_2442 |  | putative multimodular enzyme | | **1.58** | **1.40** |
| SF5M90T_2443 | *talA* | transaldolase A | | **3.45** | **3.10** |
| SF5M90T_2444 | *tktB* | transketolase 2 isozyme | | **3.58** | **3.46** |
| SF5M90T_2447 | *yffG* | putative oxidoreductase. Fe-S subunit | | **-2.74** | **-2.78** |
| SF5M90T_2468 | *hyfH* | hydrogenase 4 Fe-S subunit | | **2.00** |  |
| SF5M90T_2475 |  | putative oxidoreductase | |  | **0.80** |
| SF5M90T_2477 | *uraA* | uracil transport | | **-1.03** | **-1.33** |
| SF5M90T_2478 | *upp* | uracil phosphoribosyltransferase | | **-1.56** | **-1.38** |
| SF5M90T_2490 | *guaB* | IMP dehydrogenase | |  | **-0.54** |
| SF5M90T_2499 | *yfgB* | conserved hypothetical protein | |  | **-0.73** |
| SF5M90T_2504 | *sseB* | enhanced serine sensitivity | | **-1.06** |  |
| SF5M90T_2505 | *pepB* | putative peptidase | |  | **-0.54** |
| SF5M90T_2506 | *yfhJ* | conserved hypothetical protein | |  | **-0.49** |
| SF5M90T_2507 | *fdx* | [2FE-2S] ferredoxin. Electron carrer protein | |  | **-0.62** |
| SF5M90T_2508 | *hscA* | chaperone protein HscA | |  | **-0.89** |
| SF5M90T_2509 | *yfhE* | conserved hypothetical protein | | **-0.83** | **-0.74** |
| SF5M90T_2510 | *yfhF* | putative regulator | | **-1.82** | **-0.51** |
| SF5M90T_2511 |  | conserved hypothetical protein | | **-1.91** | **-0.63** |
| SF5M90T_2512 | *yfhO* | putative aminotransferase | |  | **-0.94** |
| SF5M90T_2513 | *iscR* | HTH-type transcriptional regulator | |  | **-0.78** |
| SF5M90T_2515 | *suhB* | inositol-1-monophosphatase | |  | **-1.30** |
| SF5M90T_2517 | *csiE* | conserved hypothetical protein | | **2.63** | **1.80** |
| SF5M90T_2525 | *yphA* | conserved hypothetical protein | | **2.45** | **2.17** |
| SF5M90T_2534 | *hmpA* | dihydropteridine reductase. Ferrisiderophore reductase activity | | **5.38** | **5.85** |
| SF5M90T_2535 | *glnB* | regulatory protein P-II for glutamine synthetase | |  | **-0.78** |
| SF5M90T_2540 | *yfhD* | putative periplasmic binding transport protein | |  | **-0.92** |
| SF5M90T_2541 | *yfhC* | putative deaminase | |  | **-0.81** |
| SF5M90T_2543 | *yfhH* | conserved hypothetical protein | | **-1.91** | **-0.77** |
| SF5M90T_2551 |  | putative tail fiber protein | |  | **-1.18** |
| SF5M90T_2574 | *yfiD* | putative formate acetyltransferase | | **-3.14** | **-3.32** |
| SF5M90T_2576 | *yfiF* | conserved hypothetical protein | |  | **-1.01** |
| SF5M90T_2577 | *trxC* | putative thioredoxin-like protein | | **1.80** |  |
| SF5M90T_2592 | *ygdH* | conserved hypothetical protein | | **1.21** | **0.76** |
| SF5M90T_2594 | *syd* | interacts with secY | | **-1.87** | **-0.52** |
| SF5M90T_2600 | *ygcX* | putative glucarate dehydratase | |  | **1.13** |
| SF5M90T_2605 | *pyrG* | CTP synthetase | |  | **-0.87** |
| SF5M90T_2622 |  | conserved hypothetical protein | | **-1.46** |  |
| SF0688 | *ybcQ* | Q protein | | **-1.98** |  |
| SF5M90T_2667 | *ygcN* | conserved hypothetical protein | | **-1.50** | **-1.13** |
| SF5M90T_2668 | *ygcM* | putative 6-pyruvoyl tetrahydrobiopterin synthase | |  | **-0.90** |
| SF5M90T_2669 | *cysJ* | sulfite reductase (NADPH), flavoprotein beta subunit | | **-2.19** | **-2.51** |
| SF5M90T_2670 | *cysI* | sulfite reductase, alpha subunit | | **-2.34** | **-2.33** |
| SF5M90T_2671 | *cysH* | 3'-phosphoadenosine 5'-phosphosulfate reductase | | **-2.19** | **-2.52** |
| SF5M90T_2674 | *cysD* | ATP:sulfurylase (ATP:sulfate adenylyltransferase), subunit 2 | | **-2.30** | **-2.21** |
| SF5M90T_2675 | *cysN* | ATP-sulfurylase (ATP:sulfate adenylyltransferase), subunit 1 | | **-2.08** | **-2.05** |
| SF5M90T_2676 | *cysC* | adenosine 5'-phosphosulfate kinase | |  | **-1.91** |
| SF5M90T_2678 |  | conserved hypothetical protein | | **-1.55** |  |
| SF5M90T_2684 | *nlpD* | lipoprotein | | **1.27** | **1.37** |
| SF5M90T_2685 | *rpoS* | RNA polymerase sigma factor | |  | **1.41** |
| SF5M90T_2689 |  | putative regulatory protein | | **-1.53** |  |
| SF5M90T_2698 | *mutS* | methyl-directed mismatch repair | |  | **-0.63** |
| SF5M90T_2699 | *ygbA* | conserved hypothetical protein | |  | **2.86** |
| SF5M90T_2701 | *hypE* | hydrogenase maturation protein | | **-2.14** | **-2.13** |
| SF5M90T_2702 | *hypD* | hydrogenase isoenzyme | | **-2.96** | **-2.57** |
| SF5M90T_2703 | *hypC* | hydrogenase assembly chaperone | | **-3.16** | **-3.91** |
| SF5M90T_2704 | *hypB* | hydrogenase nickel incorporation protein | | **-3.05** | **-2.62** |
| SF5M90T_2705 | *hypA* | probable hydrogenase nickel incorporation protein | | **-2.62** | **-2.46** |
| SF5M90T_2706 | *hycA* | transcriptional repression of hyc and hyp operons | | **-4.36** | **-3.33** |
| SF5M90T_2707 | *hycB* | small subunit of hydrogenase-3, iron- sulfur protein | | **-2.96** | **-3.87** |
| SF5M90T_2708 | *hycC* | membrane-spanning protein of hydrogenase 3 | | **-2.73** | **-2.62** |
| SF5M90T_2709 | *hycD* | membrane-spanning protein of hydrogenase 3 | |  | **-1.63** |
| SF5M90T_2710 | *hycE* | large subunit of hydrogenase 3 | | **-1.44** |  |
| SF5M90T_2716 |  | conserved hypothetical protein | | **-1.43** |  |
| SF5M90T_2720 | *hydN* | electron transport protein hydN | | **-3.96** | **-3.77** |
| SF5M90T_2721 | *hypF* | transcriptional regulatory protein | | **-2.66** | **-3.25** |
| SF5M90T_2724 | *ygaA* | putative 2-component transcriptional regulator | |  | **-0.97** |
| SF5M90T_2737 | *csrA* | carbon storage regulator | | **-1.28** |  |
| SF5M90T_2744 | *gshA* | gamma-glutamate-cysteine ligase | |  | **0.74** |
| SF5M90T_2746 | *emrB* | Multidrug resistance protein B | |  | **-0.78** |
| SF5M90T_2747 | *emrA* | pseudogene | |  | **-0.68** |
| SF5M90T_2748 | *emrR* | transcriptional repressor | |  | **-0.68** |
| SF5M90T_2759 | *ygaM* | conserved hypothetical protein | | **4.05** | **3.21** |
| SF5M90T_2762 | *stpA* | DNA-binding protein | | **-3.00** | **-2.60** |
| SF5M90T_2764 |  | conserved hypothetical protein | |  | **-0.95** |
| SF5M90T_2765 |  | conserved hypothetical protein | | **1.92** | **1.25** |
| SF5M90T_2766 | *ygaU* | conserved hypothetical protein | |  | **2.40** |
| SF5M90T_2769 | *gabT* | 4-aminobutyrate aminotransferase activity | | **3.17** | **2.18** |
| SF5M90T_2770 | *gabD* | pseudogene | | **3.46** | **3.79** |
| SF5M90T_2771 | *ygaF* | hydroxyglutarate oxidase | | **4.12** | **2.99** |
| SF5M90T_2772 |  | conserved hypothetical protein | | **3.76** | **2.97** |
| SF5M90T_2781 |  | conserved hypothetical protein | | **-2.00** |  |
| SF5M90T_2797 | *yfiL* | conserved hypothetical protein | |  | **2.00** |
| SF5M90T_2800 | *pheA* | chorismate mutase-P and prephenate dehydratase | | **1.21** | **0.79** |
| SF5M90T_2802 | *sdaC* | probable serine transporter | | **-2.23** | **-2.61** |
| SF5M90T_2803 | *sdaB* | L-serine dehydratase (deaminase). L-SD2 | | **-2.06** | **-2.60** |
| SF5M90T_2804 | *exo* | 5'-3' exonuclease | |  | **-1.03** |
| SF5M90T_2814 | *gcvA* | positive regulator of *gcv* operon | |  | **-0.60** |
| SF5M90T_2835 | *ygdP* | putative invasion protein | | **-1.71** |  |
| SF5M90T_2837 |  | putative transport protein | |  | **-0.98** |
| SF5M90T_2838 |  | conserved hypothetical protein | | **-3.10** | **-0.83** |
| SF5M90T_2839 |  | conserved hypothetical protein | | **1.59** | **0.96** |
| SF5M90T_2841 | *aas* | 2-acyl-glycerophospho-ethanolamineacyltransferase | |  | **0.61** |
| SF5M90T_2843 | *lysA* | diaminopimelate decarboxylase | | **2.44** |  |
| SF5M90T_2849 | *yqeF* | putative acyltransferase | |  | **1.21** |
| SF5M90T_2854 |  | putative lipoprotein | |  | **-0.64** |
| SF5M90T_2863 |  | putative enzyme | |  | **0.97** |
| SF5M90T_2877 | *gcvP* | glycine decarboxylase | | **2.13** | **1.69** |
| SF5M90T_2878 | *gcvH* | glycine cleavage system protein H | |  | **1.38** |
| SF5M90T_2879 | *gcvT* | aminomethyltransferase | |  | **1.44** |
| SF5M90T_2886 | *serA* | D-3-phosphoglycerate dehydrogenase | | **1.64** | **0.75** |
| SF5M90T_2894 | *yggE* | putative actin | |  | **1.01** |
| SF5M90T_2899 | *epd* | D-erythrose 4-phosphate dehydrogenase | |  | **-0.49** |
| SF5M90T_2900 |  | conserved hypothetical protein | | **-3.67** |  |
| SF5M90T_2904 |  | putative ATP-binding protein of ABC transport system | | **-2.11** |  |
| SF5M90T_2914 | *yggG* | conserved hypothetical protein | |  | **1.21** |
| SF5M90T_2915 | *speB* | agmatinase | |  | **-0.71** |
| SF5M90T_2922 | *metK* | S-adenosylmethionine synthetase | |  | **-0.68** |
| SF5M90T_2923 | *galP* | galactose-proton symport of transport system | |  | **0.82** |
| SF5M90T_2925 | *endA* | DNA-specific endonuclease I | | **-3.26** | **-2.74** |
| SF5M90T_2940 | *yggM* | putative alpha helix chain | |  | **-2.50** |
| SF5M90T_2941 | *ansB* | periplasmic L-asparaginase II | | **-4.49** | **-3.87** |
| SF5M90T_2943 | *yggL* | conserved hypothetical protein | | **-2.39** |  |
| SF5M90T_2944 | *yggH* | conserved hypothetical protein | | **-1.18** | **-0.61** |
| SF5M90T_2951 | *yqgA* | putative transport protein | |  | **-1.59** |
| SF5M90T_2957 | *glcB* | malate synthase G | |  | **1.69** |
| SF5M90T_2958 | *glcG* | conserved hypothetical protein | |  | **1.77** |
| SF5M90T_2959 | *glcF* | pseudogene | | **1.54** | **2.35** |
| SF5M90T_2961 | *glcC* | transcriptional activator | | **1.51** | **2.54** |
| SF5M90T_2967 | *gsp* | glutathionylspermidine synthetase/amidase | |  | **0.70** |
| SF5M90T_2968 |  | conserved hypothetical protein | |  | **1.97** |
| SF5M90T_2969 | *hybG* | hydrogenase-2 operon protein | |  | **-1.92** |
| SF5M90T_2970 | *hybF* | probable hydrogenase nickel incorporation protein | | **-2.32** | **-2.03** |
| SF5M90T_2971 | *hybE* | hydrogenase 2-specific chaperone | | **-1.82** | **-1.87** |
| SF5M90T_2972 | *hybD* | probable processing element for hydrogenase-2 | | **-1.96** | **-1.81** |
| SF5M90T_2973 | *hybC* | probable large subunit hydrogenase-2 | | **-1.75** | **-1.97** |
| SF5M90T_2974 | *hybB* | probable cytochrome Ni/Fe component of hydrogenase-2 | | **-1.92** | **-2.20** |
| SF5M90T_2975 | *hybA* | hydrogenase-2 small subunit | | **-2.34** | **-2.42** |
| SF5M90T_2976 |  | putative hydrogenase subunit | | **-2.49** | **-2.40** |
| SF5M90T_2978 | *yghYX* | predicted hydrolase | | **3.65** | **2.50** |
| SF5M90T_2981 | *yghA* | putative oxidoreductase | | **2.44** | **2.30** |
| SF5M90T_2982 | *exbD* | biopolymer transport | | **-1.15** |  |
| SF5M90T_2983 | *exbB* | biopolymer transport | |  | **-0.86** |
| SF3052 |  | conserved hypothetical protein | |  | **-1.73** |
| SF5M90T_2988 | *yqhE* | conserved hypothetical protein | | **1.52** | **2.46** |
| SF5M90T_2995 |  | conserved hypothetical protein | |  | **2.43** |
| SF5M90T_2996 | *ygiW* | conserved hypothetical protein | | **2.12** | **3.27** |
| SF3076 | *ygiA* | conserved hypothetical protein | | **-1.48** |  |
| SF5M90T_3007 | *ygiB* | conserved hypothetical protein | |  | **0.66** |
| SF5M90T_3008 | *ygiC* | putative synthetase/amidase | |  | **0.64** |
| SF5M90T_3012 |  | conserved hypothetical protein | | **-2.10** |  |
| SF5M90T_3022 | *bacA* | undecaprenyl pyrophosphate phosphatase | |  | **-0.71** |
| SF5M90T_3023 | *ygiG* | putative kinase | | **-2.08** | **-0.86** |
| SF5M90T_3025 | *ygiP* | putative transcriptional regulator/ nucleoid-associated protein | | **-4.57** | **-2.45** |
| SF5M90T_3028 | *ygjE* | conserved hypothetical protein | |  | **-1.16** |
| SF5M90T_3029 | *ygjD* | putative O-sialoglycoprotein endopeptidase | | **-1.05** |  |
| SF5M90T_3031 | *dnaG* | DNA primase | |  | **-0.63** |
| SF5M90T_3034 | *ygjF* | G/U mismatch-specific DNA glycosylase | |  | **1.10** |
| SF5M90T_3037 | *yqjI* | putative transcriptional regulator | | **-1.21** |  |
| SF5M90T_3039 | *ygjG* | probable ornithine aminotransferase | | **3.49** | **2.60** |
| SF5M90T_3041 | *ebgR* | regulator of ebg operon | | **-1.42** | **-1.03** |
| SF5M90T_3050 | *ygjO* | putative enzyme | |  | **-1.04** |
| SF5M90T_3053 | *ygjR* | conserved hypothetical protein | |  | **1.02** |
| SF5M90T_3063 | *exuR* | *exu* regulon transcriptional regulator | |  | **-0.67** |
| SF5M90T_3066 | *yqjC* | conserved hypothetical protein | |  | **1.97** |
| SF5M90T_3067 | *yqjD* | conserved hypothetical protein | | **1.78** | **2.05** |
| SF5M90T_3068 | *yqjE* | conserved hypothetical protein | | **2.35** | **2.08** |
| SF5M90T_3069 |  | conserved hypothetical protein | | **1.97** | **2.11** |
| SF5M90T_3071 | *yqjG* | putative transferase | | **1.58** | **1.53** |
| SF5M90T_3075 | *yhaL* | conserved hypothetical protein | |  | **-0.76** |
| SF5M90T_3084 | *tdcA* | transcriptional activator of *tdc* operon | |  | **-1.41** |
| SF5M90T_3106 | *yraQ* | conserved hypothetical protein | |  | **-1.28** |
| SF5M90T_3109 | *yhbP* | conserved hypothetical protein | |  | **-1.18** |
| SF5M90T_3111 | *yhbS* | putative acetyltransferase | | **-1.17** | **-1.39** |
| SF5M90T_3112 | *yhbT* | putative lipid carrier protein | | **-1.33** | **-1.19** |
| SF5M90T_3113 | *yhbU* | putative collagenase | | **-6.25** | **-7.22** |
| SF5M90T_3114 | *yhbV* | predicted protease | | **-5.06** | **-4.72** |
| SF5M90T_3115 | *yhbW* | putative enzyme | | **1.62** |  |
| SF5M90T_3123 | *infB* | protein chain initiation factor IF-2 | | **-1.07** |  |
| SF5M90T_3127 | *argG* | argininosuccinate synthetase | |  | **0.67** |
| SF5M90T_3130 | *secG* | protein export-membrane protein | |  | **-0.86** |
| SF5M90T_3135 | *yhbY* | conserved hypothetical protein | |  | **-0.65** |
| SF5M90T_3138 | *yhbZ* | putative GTP-binding factor | | **-0.95** | **-0.74** |
| SF5M90T_3139 | *yhbE* | putative permeases of drug/metabolite transporter superfamily | |  | **-0.90** |
| SF5M90T_3148 | *yrbD* | conserved hypothetical protein | |  | **-0.57** |
| SF5M90T_3149 | *yrbE* | conserved hypothetical protein | |  | **-0.59** |
| SF5M90T_3150 | *yrbF* | putative ATP-binding component of a transport system | |  | **-0.72** |
| SF5M90T_3162 | *yrbL* | conserved hypothetical protein | |  | **0.75** |
| SF5M90T_3166 | *yhcC* | conserved hypothetical protein | | **-2.57** | **-3.86** |
| SF5M90T_3167 | *gltB* | glutamate synthase large subunit | | **1.49** | **1.10** |
| SF5M90T_2447 | *gltD* | glutamate synthase small subunit | |  | **1.26** |
| SF5M90T_3171 | *yhcH* | conserved hypothetical protein | |  | **1.73** |
| SF5M90T_3172 | *yhcI* | putative NAGC-like transcriptional regulator | |  | **1.51** |
| SF5M90T_3180 | *sspB* | stringent starvation protein B | |  | **-0.59** |
| SF5M90T_3185 | *yhcB* | conserved hypothetical protein | |  | **-0.51** |
| SF5M90T_3188 | *mdh* | malate dehydrogenase | | **2.11** | **1.25** |
| SF5M90T_3189 | *argR* | repressor of *arg* regulon | |  | **-0.80** |
| SF5M90T_3190 | *yhcN* | conserved hypothetical protein | | **-1.42** |  |
| SF5M90T_3191 | *yhcO* | conserved hypothetical protein | |  | **1.97** |
| SF5M90T_3194 | *yhcR* | conserved hypothetical protein | |  | **-1.18** |
| SF5M90T_3204 | *yhdH* | putative dehydrogenase | |  | **-0.83** |
| SF5M90T_3210 | *yhdG* | putative dehydrogenase | |  | **-1.27** |
| SF5M90T_3211 | *fis* | DNA-binding protein | | **-1.28** | **-1.58** |
| SF5M90T_3215 | *yhdW* | putative periplasmic binding transport protein | | **3.31** | **2.45** |
| SF5M90T_3218 | *yhdZ* | putative ATP-binding component of a transport system | |  | **1.74** |
| SF5M90T_3226 | *yrdB* | conserved hypothetical protein | |  | **0.82** |
| SF5M90T_3227 | *aroE* | dehydroshikimate reductase | |  | **0.69** |
| SF5M90T_3228 | *yrdC* | conserved hypothetical protein | |  | **0.91** |
| SF5M90T_3244 | *prlA* | putative ATPase subunit of translocase | | **-1.22** |  |
| SF5M90T_3266 | *bfr* | bacterioferrin. An iron storage homoprotein | | **3.19** | **2.87** |
| SF5M90T_3267 | *yheA* | conserved hypothetical protein | | **-2.22** |  |
| SF5M90T_3278 | *slyX* | host factor for lysis of phiX174 infection | |  | **-0.89** |
| SF5M90T_3281 | *kefB* | glutathione-regulated potassium-efflux system protein | |  | **0.92** |
| SF5M90T_3282 | *yheR* | putative NAD(P)H oxidoreductase | |  | **1.77** |
| SF5M90T_3290 | *argD* | acetylornithine delta-aminotransferase | |  | **-0.62** |
| SF5M90T_3292 | *fic* | cell filamentation protein | | **1.67** | **2.04** |
| SF5M90T_3293 | *yhfG* | conserved hypothetical protein | |  | **1.14** |
| SF5M90T_3294 | *ppiA* | peptidyl-prolyl cis-trans isomerase A | |  | **0.47** |
| SF5M90T_3295 | *yhfC* | putative transport | | **-1.30** | **-1.25** |
| SF5M90T_3296 | *nirB* | nitrite reductase (NAD(P)H) subunit | | **-6.22** | **-5.42** |
| SF5M90T_3297 | *nirD* | nitrite reductase (NAD(P)H) subunit | | **-5.58** | **-4.85** |
| SF5M90T_3298 | *nirC* | nitrite transporter | | **-4.86** | **-4.47** |
| SF5M90T_3299 | *cysG* | sirohaeme biosynthesis | |  | **-1.45** |
| SF5M90T_3309 | *yhfV* | putative hydrolase | | **-2.39** |  |
| SF5M90T_3310 | *yhfW* | putative mutase | | **-1.44** |  |
| SF5M90T_3333 | *pckA* | phosphoenolpyruvate carboxykinase | |  | **0.62** |
| SF5M90T_3336 | *greB* | transcription elongation factor | | **-1.53** |  |
| SF5M90T_3338 | *feoA* | ferrous iron transport protein A | | **-2.53** | **-2.78** |
| SF5M90T_3339 | *feoB* | ferrous iron transport protein B | | **-1.13** | **-1.54** |
| SF5M90T_3340 | *yhgG* | conserved hypothetical protein | |  | **-1.14** |
| SF5M90T_3347 | *malQ* | 4-alpha-glucanotransferase (amylomaltase) | |  | **0.77** |
| SF5M90T_3348 | *malP* | maltodextrin phosphorylase | |  | **0.78** |
| SF5M90T_3349 | *malT* | positive regulator of mal regulon | |  | **0.58** |
| SF5M90T_3353 | *glpR* | repressor of the glp operon | |  | **0.56** |
| SF5M90T_3355 | *glpE* | thiosulfate sulfurtransferase | |  | **1.19** |
| SF5M90T_3356 | *glpD* | sn-glycerol-3-phosphate dehydrogenase (aerobic) | | **3.12** | **3.08** |
| SF5M90T_3348 | *glgP* | glycogen phosphorylase | |  | **1.34** |
| SF5M90T_3360 | *glgA* | glycogen synthase | | **1.40** | **1.28** |
| SF5M90T_3361 | *glgC* | glucose-1-phosphate adenylyltransferase | | **2.01** | **1.32** |
| SF5M90T_3362 | *glgX* | glycogen debranching enzyme | | **1.42** | **1.00** |
| SF5M90T_3363 | *glgB* | 1.4-alpha-glucan branching enzyme | |  | **1.05** |
| SF5M90T_3366 | *gntU* | pseudogene | | **-1.41** | **-0.60** |
| SF5M90T_3367 | *gntK* | thermoresistant gluconokinase | |  | **-0.79** |
| SF5M90T_3370 | *yhhX* | putative regulator | |  | **1.30** |
| SF5M90T_3372 | *ggt* | gamma-glutamyltranspeptidase | | **2.92** | **2.69** |
| SF5M90T_3375 | *ugpC* | pseudogene | |  | **0.99** |
| SF5M90T_3378 | *ugpB* | glycerol-3-phosphate-binding periplasmic protein precursor | | **2.36** | **2.28** |
| SF5M90T_3388 | *ftsE* | cell division ATP-binding protein | |  | **-0.63** |
| SF5M90T_3395 | *yhhP* | conserved hypothetical protein | |  | **-0.83** |
| SF5M90T_3396 | *yhhQ* | conserved hypothetical protein | |  | **-0.99** |
| SF5M90T_3401 | *nikA* | periplasmic binding protein for nickel | | **-5.12** | **-4.97** |
| SF5M90T_3402 | *nikB* | transport of nickel. Membrane protein | | **-4.38** | **-4.25** |
| SF5M90T_3403 | *nikC* | transport of nickel. Membrane protein | | **-4.66** | **-4.14** |
| SF5M90T_3404 | *nikD* | ATP-binding protein of nickel transport system | | **-4.29** | **-3.39** |
| SF5M90T_3405 | *nikE* | ATP-binding protein of nickel transport system | | **-4.43** | **-4.32** |
| SF5M90T_3406 | *yhhG* | nickel-responsive regulator | | **-1.30** | **-2.39** |
| SF5M90T_3408 | *yhhJ* | putative transporter | |  | **1.67** |
| SF5M90T_3409 | *yhiH* | hypothetical ABC transporter ATP-binding protein | | **1.52** | **1.32** |
| SF5M90T_3410 | *yhiI* | putative membrane protein | | **1.19** | **0.83** |
| SF5M90T_3411 | *yhiJ* | conserved hypothetical protein | | **-2.99** |  |
| SFxv_3833 |  | hypothetical protein | | **-4.23** |  |
| SF5M90T_3425 | *yhiN* | predicted flavoprotein | | **-1.17** | **-1.15** |
| SF5M90T_3426 | *pitA* | low-affinity phosphate transport | |  | **-1.03** |
| SF5M90T_3427 | *yhiO* | conserved hypothetical protein | |  | **1.26** |
| SF5M90T_3431 | *prlC* | oligopeptidase A | |  | **0.54** |
| SF5M90T_3441 | *slp* | outer membrane protein induced after carbon starvation | | **2.74** | **2.38** |
| SF5M90T_3442 | *yhiF* | conserved hypothetical protein | |  | **2.44** |
| SF5M90T_3443 | *yhiD* | pseudogene | | **2.39** | **2.27** |
| SF5M90T_3444 | *hdeB* | acid stress chaperone | |  | **2.57** |
| SF5M90T_3445 | *hdeA* | acid stress chaperone | | **1.67** | **2.35** |
| SF5M90T_3446 | *hdeD* | acid-resistance membrane protein | | **2.53** | **2.27** |
| SF5M90T_3447 | *yhiE* | putative DNA binding transcription factor | |  | **1.29** |
| SF5M90T_3448 | *yhiU* | pseudogene | | **2.07** | **1.90** |
| SF5M90T_3453 | *yiaG* | putative transcriptional regulator | |  | **3.65** |
| SF5M90T_3459 | *tag* | 3-methyl-adenine DNA glycosylase I | |  | **0.92** |
| SF5M90T_3460 | *yhjY* | putative lipase | | **2.43** | **1.61** |
| SF5M90T_3461 | *yhjX* | putative resistance protein | |  | **1.88** |
| SF5M90T_3462 | *yhjW* | conserved hypothetical protein | |  | **-0.67** |
| SF5M90T_3464 | *dppA* | dipeptide transport protein | | **1.87** | **1.82** |
| SF5M90T_3465 | *dppB* | dipeptide transport system permease protein 1 | |  | **1.74** |
| SF5M90T_3467 | *dppD* | putative ATP-binding component of dipeptide transport system | |  | **1.39** |
| SF5M90T_3468 | *dppF* | putative ATP-binding component of dipeptide transport system | |  | **1.79** |
| SF5M90T_3469 | *yhjV* | putative transporter protein | |  | **-0.78** |
| SF5M90T_3479 | *yhjL* | pseudogene | | **1.66** | **1.81** |
| SF5M90T_3481 | *dctA* | uptake of C4-dicarboxylic acids | | **2.44** | **1.90** |
| SF5M90T_3483 | *kdgK* | ketodeoxygluconokinase | |  | **-0.74** |
| SF5M90T_3485 | *yhjG* | conserved hypothetical protein | | **1.25** | **2.21** |
| SF5M90T_3487 | *yhjD* | conserved hypothetical protein | | **1.62** | **1.43** |
| SF5M90T_3488 | *yhjC* | putative transcriptional regulator LYSR-type | |  | **-1.03** |
| SF5M90T_3497 | *cdh* | CDP-diacylglycerol phosphotidylhydrolase | | **-1.75** | **-1.61** |
| SF5M90T_3498 | *sbp* | periplasmic sulfate-binding protein | | **-2.27** | **-2.25** |
| SF5M90T_3501 | *yiiO* | uncharacterized periplasmic protein | |  | **2.64** |
| SF5M90T_3508 | *sodA* | superoxide dismutase | | **1.22** | **1.13** |
| SF5M90T_1706 | *fdoG* | formate dehydrogenase-O major subunit | | **2.97** | **2.67** |
| SF5M90T_3523 | *fdoH* | formate dehydrogenase-O iron-sulfur subunit | | **2.65** | **2.49** |
| SF5M90T_3524 | *fdoI* | formate dehydrogenase cytochrome B556 (FDO) subunit | |  | **1.81** |
| SF5M90T_3525 | *fdhE* | affects formate dehydrogenase-N | |  | **1.02** |
| SF5M90T_3532 | *yiiD* | putative acetyltransferase | |  | **0.66** |
| SF5M90T_3533 | *yihZ* | conserved hypothetical protein | |  | **0.58** |
| SF5M90T_3534 | *rbn* | tRNA processing exoribonuclease BN | |  | **0.76** |
| SF5M90T_3535 | *yihX* | putative phosphatase | |  | **0.50** |
| SF5M90T_3536 | *yihW* | putative DEOR-type transcriptional regulator | |  | **-0.59** |
| SF5M90T_3551 | *glnA* | glutamine synthetase | |  | **-1.11** |
| SF5M90T_3555 | *yihI* | conserved hypothetical protein | | **-1.83** |  |
| SF5M90T_3558 | *yihG* | putative endonuclease | | **-1.57** | **-0.73** |
| SF5M90T_3574 | *fadB* | 3-hydroxyacyl-CoA dehydrogenase | |  | **1.59** |
| SF5M90T_3575 | *fadA* | acetyl-CoA transferase | | **2.26** | **1.56** |
| SF5M90T_3576 | *ubiB* | NAD(P)H-flavin reductase | |  | **0.56** |
| SF5M90T_3578 | *rfaH* | transcriptional activator | |  | **-0.96** |
| SF5M90T_3580 | *tatC* | Sec-independent protein translocase | |  | **-0.57** |
| SF5M90T_3582 | *tatA* | Sec-independent protein translocase | | **-1.38** |  |
| SF5M90T_3587 | *udp* | uridine phosphorylase | |  | **0.59** |
| SF5M90T_3588 | *ysgA* | putative hydrolase | | **3.64** | **2.55** |
| SF5M90T_3589 | *metE* | tetrahydropteroyltriglutamate methyltransferase | | **1.74** | **0.99** |
| SF5M90T_3598 | *yigI* | conserved hypothetical protein | |  | **1.81** |
| SF5M90T_3602 | *corA* | Mg2+ transport system I | |  | **-0.71** |
| SF5M90T_3618 | *hemX* | uroporphyrinogen III methylase | |  | **0.59** |
| SF5M90T_3632 | *wecD* | conserved hypothetical protein | |  | **-0.80** |
| SF5M90T_3633 | *rffH* | glucose-1-phosphate thymidylyltransferase | |  | **-0.90** |
| SF5M90T_3634 | *rffG* | dTDP-glucose 4.6-dehydratase | |  | **-0.79** |
| SF5M90T_3635 | *wecC* | UDP-N-acetyl-D-mannosaminuronic acid dehydrogenase | |  | **-0.70** |
| SF5M90T_3636 | *wecB* | UDP-N-acetyl glucosamine-2-epimerase | |  | **-0.75** |
| SF5M90T_3637 | *wzzE* | putative transport protein | |  | **-0.57** |
| SF5M90T_3638 | *rfe* | undecaprenyl-phosphatealpha-N-acetylglucosaminyltransferase | |  | **-0.91** |
| SF5M90T_3639 |  | conserved hypothetical protein | |  | **-0.79** |
| SF5M90T_3645 | *ppiC* | peptidyl-prolyl cis-trans isomerase C | | **-1.92** | **-0.85** |
| SF5M90T_3648 | *ilvA* | threonine dehydratase | |  | **0.59** |
| SF3842 | *ilvL* | ilvGEDA operon leader peptide | |  | **-1.19** |
| SF5M90T_3654 | *yifE* | conserved hypothetical protein | |  | **-1.21** |
| SF5M90T_3666 | *rbsB* | pseudogene | | **-2.31** |  |
| SF5M90T_3667 | *rbsC* | D-ribose high-affinity transport system | | **1.81** | **1.92** |
| SF5M90T_3671 | *kup* | low affinity potassium transport system | | **-2.25** | **-2.58** |
| SF5M90T_3672 | *glmS* | L-glutamine:D-fructose-6-phosphateaminotransferase | |  | **-0.76** |
| SF5M90T_3683 | *gidB* | glucose-inhibited division | |  | **-1.01** |
| SF5M90T_3684 | *gidA* | glucose-inhibited division | |  | **-0.86** |
| SF5M90T_3685 | *mioC* | initiation of chromosome replication | |  | **-0.71** |
| SF5M90T_3687 | *asnA* | asparagine synthetase A | |  | **-1.61** |
| SF5M90T_3688 | *yieM* | conserved hypothetical protein | |  | **0.92** |
| SF5M90T_3689 | *yieN* | putative 2-component regulator | |  | **0.72** |
| SF5M90T_3712 | *yieF* | conserved hypothetical protein | | **1.61** | **0.94** |
| SF5M90T_3713 | *yieE* | predicted phosphopantetheinyl transferase | |  | **0.52** |
| SF5M90T_3721 | *yidC* | inner-membrane protein | |  | **-0.65** |
| SF5M90T_3722 | *rnpA* | ribonuclease P | |  | **-0.87** |
| SF5M90T_3725 | *dnaN* | DNA polymerase III beta-subunit | |  | **-0.51** |
| SF5M90T_3726 | *recF* | DNA replication and repair protein | |  | **-0.63** |
| SF5M90T_3730 |  | putative replicase | | **-1.83** |  |
| SF5M90T_3734 | *dgoT* | D-galactonate transport | | **-1.95** |  |
| SF5M90T_3752 | *ilvB* | acetolactate synthase I large subunit | | **1.57** | **1.53** |
| SF5M90T_3753 | *ilvN* | acetolactate synthase I small subunit | |  | **2.07** |
| SF5M90T_3755 | *uhpB* | sensor protein | |  | **-0.64** |
| SF5M90T_3756 | *uhpC* | regulator of *uhpT* | |  | **-0.67** |
| SF5M90T_3788 | *gltS* | glutamate transport | |  | **-0.72** |
| SF5M90T_3790 | *spoU* | putative RNA methylase | |  | **-0.71** |
| SF5M90T_3796 | *yicG* | conserved hypothetical protein | |  | **-0.95** |
| SF5M90T_3799 | *rph* | RNase PH | |  | **-0.77** |
| SF5M90T_3800 | *pyrE* | orotate phosphoribosyltransferase | |  | **-0.84** |
| SF5M90T_3801 | *ttk* | putative transcriptional regulator | |  | **-0.60** |
| SF5M90T_3802 | *dut* | deoxyuridinetriphosphatase | |  | **-0.46** |
| SF5M90T_3808 | *kdtA* | 3-deoxy-D-manno-octulosonic-acid transferase | |  | **-0.76** |
| SF5M90T_3814 | *waaL* | lipid A-core surface polymer ligase | | **-2.09** |  |
| SF5M90T_3815 | *waaY* | putative LPS biosynthesis protein | | **-2.30** |  |
| SF5M90T_3817 | *waaD* | lipopolysaccharide1.2-N-acetylglucosaminetransferase | | **-1.49** |  |
| SF5M90T_3818 | *waaI* | lipid A-core surface polymer ligase | | **-1.67** |  |
| SF5M90T_3822 | *htrL* | uncharacterized protein | | **-2.53** |  |
| SF5M90T_3828 | *yibP* | putative membrane protein | |  | **-0.76** |
| SF5M90T_3838 | *lldD* | L-lactate dehydrogenase | | **5.97** | **5.73** |
| SF5M90T_3839 | *lldR* | transcriptional regulator | | **5.94** | **6.24** |
| SF5M90T_3840 | *lldP* | L-lactate permease | | **6.41** | **6.04** |
| SF5M90T_3853 | *yibF* | putative S-transferase | | **1.50** | **0.90** |
| SF5M90T_3854 | *selA* | selenocysteine synthase | |  | **-0.66** |
| SF5M90T_3855 | *selB* | selenocysteinyl-tRNA-specific translation factor | |  | **-0.59** |
| SF5M90T_3856 | *yiaY* | putative oxidoreductase | | **-2.73** | **-2.67** |
| SF5M90T_3857 | *aldB* | aldehyde dehydrogenase B | | **2.83** | **2.23** |
| SF5M90T_3865 |  | conserved hypothetical protein | | **1.61** |  |
| SF5M90T_3877 | *yiaK* | putative dehydrogenase | | **1.22** | **0.93** |
| SF5M90T_3878 | *yiaJ* | putative regulator | |  | **0.63** |
| SF5M90T_3879 | *yiaI* | conserved hypothetical protein | |  | **-0.82** |
| SF5M90T_3880 | *avtA* | valine--pyruvate transaminase | |  | **0.61** |
| SF5M90T_3882 | *bax* | putative ATP-binding protein | |  | **-0.77** |
| SF5M90T_3888 | *xylB* | xylulokinase | | **1.92** |  |
| SF5M90T_3890 | *yiaA* | conserved hypothetical protein | | **-3.51** |  |
| SF5M90T_3893 | *glyQ* | glycine tRNA synthetase alpha subunit | |  | **-0.50** |
| SF5M90T_3899 | *yhiW* | putative ARAC-type regulatory protein | | **3.27** | **3.29** |
| SF5M90T_3900 | *yhiX* | putative ARAC-type regulatory protein | | **2.60** | **3.25** |
| SF5M90T_3901 | *gadA* | glutamate decarboxylase isozyme | | **5.14** | **4.96** |
| SF5M90T_3908 | *glpX* | unknown function in glycerol metabolism | |  | **-1.25** |
| SF5M90T_3910 | *glpF* | facilitated diffusion of glycerol | | **1.75** | **1.58** |
| SF5M90T_3911 | *yiiU* | conserved hypothetical protein | | **-2.49** |  |
| SF5M90T_3919 | *yiiX* | conserved hypothetical protein | |  | **-1.46** |
| SF5M90T_3920 | *metJ* | transcriptional repressor protein | | **-1.54** | **-0.82** |
| SF5M90T_3924 | *katG* | catalase; hydroperoxidase HPI(I) | |  | **-0.58** |
| SF5M90T_3927 | *gldA* | pseudogene | | **-2.41** | **-1.88** |
| SF5M90T_3928 | *talC* | pseudogene | |  | **-1.61** |
| SF5M90T_3937 | *ppc* | phosphoenolpyruvate carboxylase | |  | **0.64** |
| SF5M90T_3940 | *argB* | acetylglutamate kinase | |  | **0.94** |
| SF5M90T_3941 | *argH* | argininosuccinate lyase | |  | **0.87** |
| SF5M90T_3946 | *udhA* | soluble pyridine nucleotide transhydrogenase | | **2.16** | **1.74** |
| SF5M90T_3947 | *yijC* | conserved hypothetical protein | |  | **-0.49** |
| SF5M90T_3949 | *trmA* | tRNA (uracil-5-)-methyltransferase | | **-1.23** | **-0.89** |
| SF5M90T_3951 | *murI* | glutamate racemase | |  | **-0.48** |
| SF5M90T_3956 | *murB* | UDP-N-acetylenolpyruvoylglucosamine reductase | |  | **-0.60** |
| SF5M90T_3957 | *birA* | biotin--protein ligase | |  | **-0.51** |
| SF5M90T_3963 | *tufB* | elongation factor EF-Tu | |  | **-0.89** |
| SF5M90T_3964 | *secE* | preprotein translocase | | **-1.06** | **-0.76** |
| SF5M90T_3965 | *nusG* | component in transcription antitermination | | **-1.05** | **-0.74** |
| SF5M90T_3978 | *yjaE* | putative transcriptional regulator | |  | **1.16** |
| SF5M90T_3979 | *yjaD* | conserved hypothetical protein | |  | **0.93** |
| SF5M90T_3984 | *yjaH* | conserved hypothetical protein | |  | **-0.57** |
| SF5M90T_3985 | *yjaI* | conserved hypothetical protein | | **3.05** | **3.98** |
| SF5M90T_4001 | *metH* | B12-dependent methionine synthase | |  | **0.88** |
| SF5M90T_4002 | *yjbB* | putative alpha helix protein | | **1.24** | **1.10** |
| SF5M90T_4003 | *pepE* | peptidase E | | **-2.09** | **-2.89** |
| SF5M90T_4011 | *yjbC* | conserved hypothetical protein | |  | **-0.73** |
| SF5M90T_4012 | *yjbD* | conserved hypothetical protein | | **-1.51** |  |
| SF5M90T_4017 | *dcuB* | anaerobic dicarboxylate transport | | **-4.09** | **-3.94** |
| SF5M90T_4018 | *fumB* | fumarate hydratase class I | | **-3.65** | **-3.97** |
| SF5M90T_4023 | *adiA* | biodegradative arginine decarboxylase | | **-1.15** | **-1.65** |
| SF5M90T_4025 | *yjdE* | putative amino acid/amine transport protein | | **-1.27** | **-1.54** |
| SF5M90T_4029 | *proP* | low-affinity transport system; proline permease II | | **1.05** | **0.75** |
| SF5M90T_4032 | *phnA* | conserved hypothetical protein | |  | **-1.11** |
| SF5M90T_4033 | *phnB* | conserved hypothetical protein | | **2.90** |  |
| SF5M90T_4042 | *fdhF* | pseudogene | | **-2.87** | **-3.44** |
| SF5M90T_4043 | *yjcO* | conserved hypothetical protein | |  | **0.79** |
| SF5M90T_4046 | *nrfF* | formate-dependent nitrite reductase complex subunit | |  | **-1.97** |
| SF5M90T_4047 | *nrfE* | heme lyase subunit | | **-1.81** | **-2.40** |
| SF5M90T_4048 | *nrfD* | formate-dependent nitrite reductase transmembrane protein | | **-2.17** | **-2.92** |
| SF5M90T_4049 | *nrfC* | formate-dependent nitrite reductase | | **-3.31** | **-3.10** |
| SF5M90T_4050 | *nrfB* | cytochrome c nitrite reductase pentaheme subunit | | **-3.28** | **-3.20** |
| SF5M90T_4051 | *nrfA* | cytochrome c552 precursor | | **-3.68** | **-3.32** |
| SF5M90T_4060 | *soxS* | regulation of superoxide response regulon | |  | **-1.65** |
| SF5M90T_4061 | *yjcC* | conserved hypothetical protein | | **1.56** | **1.45** |
| SF5M90T_4066 | *yjbR* | conserved hypothetical protein | |  | **1.39** |
| SF5M90T_4069 | *tyrB* | aspartate aminotransferase | |  | **0.54** |
| SF5M90T_4072 | *qor* | quinone oxidoreductase | | **1.67** | **1.58** |
| SF5M90T_4077 | *yjbK* | putative regulator | |  | **-0.68** |
| SF5M90T_4078 | *yjbJ* | conserved hypothetical protein | |  | **1.87** |
| SF5M90T_4079 | *dinF* | DNA-damage-inducible protein F | |  | **-0.72** |
| SF5M90T_4080 | *lexA* | regulator for SOS(lexA) regulon | |  | **-0.63** |
| SF5M90T_4083 | *ubiA* | 4-hydroxybenzoate-octaprenyltransferase | |  | **0.58** |
| SF5M90T_4094 | *yjbA* | P-starvation inducible protein PsiE | | **1.69** |  |
| SF5M90T_4098 | *pgi* | glucosephosphate isomerase | |  | **0.74** |
| SF5M90T_4099 | *lysC* | aspartokinase III | | **1.37** | **1.14** |
| SF5M90T_4108 | *yjiD* | conserved hypothetical protein | |  | **-1.93** |
| SF5M90T_4110 | *uxuR* | regulator for uxu operon | |  | **-0.53** |
| SF5M90T_4137 | *yjgB* | putative oxidoreductase | | **3.50** | **2.99** |
| SF5M90T_4138 | *yjgR* | conserved hypothetical protein | | **1.61** | **2.06** |
| SF5M90T_4146 | *yjgD* | conserved hypothetical protein | | **-2.30** |  |
| SF5M90T_4158 | *mgtA* | Mg2+ transport ATPase. P-type 1 | |  | **-1.54** |
| SF5M90T_4159 | *treR* | repressor | |  | **-0.73** |
| SF5M90T_4161 | *nrdD* | anaerobic ribonucleoside-triphosphate reductase | | **-2.74** | **-2.90** |
| SF5M90T_4162 | *nrdG* | anaerobic ribonucleotide reductase activating protein | | **-2.81** | **-2.68** |
| SF5M90T_4163 | *cybC* | cytochrome b(562) | | **1.07** | **1.76** |
| SF5M90T_4165 | *yjgA* | putative alpha helix protein | |  | **-0.76** |
| SF5M90T_4177 | *msrA* | peptide methionine sulfoxide reductase | | **2.21** | **1.54** |
| SF5M90T_4185 | *ytfF* | putative transmembrane subunit | | **2.71** |  |
| SF5M90T_4186 | *ytfE* | conserved hypothetical protein | | **4.42** | **3.70** |
| SF5M90T_4190 | *ytfA* | conserved hypothetical protein | | **-1.87** |  |
| SF5M90T_4197 | *yjdC* | putative transcriptional regulator | |  | **1.37** |
| SF5M90T_4200 | *dcuA* | anaerobic dicarboxylate transport | | **-1.85** | **-1.51** |
| SF5M90T_4201 | *aspA* | aspartate ammonia-lyase (aspartase) | | **-2.59** | **-2.27** |
| SF5M90T_4211 | *efp* | elongation factor P (EF-P) | |  | **-0.90** |
| SF4304 | *ecnA* | putative antitoxin of osmotically regulated toxin-antitoxin system | |  | **-0.71** |
| SF5M90T_4212 | *ecnB* | putative toxin of osmotically regulated toxin- antitoxin system | |  | **2.87** |
| SF5M90T_4213 | *sugE* | suppresser of groEL | |  | **1.48** |
| SF5M90T_4214 | *blc* | outer membrane lipoprotein | | **3.19** | **2.82** |
| SF5M90T_4215 | *ampC* | beta-lactamase; penicillin resistance | |  | **-1.69** |
| SF5M90T_4216 | *frdD* | fumarate reductase membrane anchor polypeptide | |  | **-2.48** |
| SF5M90T_4217 | *frdC* | fumarate reductase membrane anchor polypeptide | | **-2.16** | **-2.55** |
| SF5M90T_4218 | *frdB* | fumarate reductase iron-sulfur protein subunit | | **-2.08** | **-2.49** |
| SF5M90T_4219 | *frdA* | fumarate reductase flavoprotein subunit | | **-2.34** | **-2.54** |
| SF5M90T_4220 | *yjeA* | putative lysyl-tRNA synthetase | | **-1.75** | **-1.72** |
| SF5M90T_4221 | *yjeM* | pseudogene | |  | **-0.73** |
| SF5M90T_4236 | *hfq* | RNA-binding protein | | **-2.10** |  |
| SF5M90T_4237 | *hflX* | GTP-binding protein | |  | **0.63** |
| SF5M90T_4238 | *hflK* | protease specific for phage lambda cII repressor | |  | **0.59** |
| SF5M90T_4239 | *hflC* | protease specific for phage lambda cII repressor | |  | **0.58** |
| SF5M90T_4241 | *purA* | adenylosuccinate synthetase | |  | **-0.77** |
| SF5M90T_4249 | *aidB* | putative acyl coenzyme A dehydrogenase | | **3.17** | **2.84** |
| SF5M90T_4250 | *yjfN* | conserved hypothetical protein | |  | **1.52** |
| SF5M90T_4251 | *yjfO* | conserved hypothetical protein | | **1.55** | **1.68** |
| SF5M90T_4253 | *yjfQ* | putative DEOR-type transcriptional regulator | |  | **-0.83** |
| SF5M90T_4261 | *yjfY* | conserved hypothetical protein | |  | **1.85** |
| SF5M90T_4271 |  | conserved hypothetical protein | | **-2.92** | **-1.04** |
| SF5M90T_4272 |  | putative restriction modification enzyme M subunit | | **-1.02** | **-0.86** |
| SF5M90T_4273 |  | putative restriction modification enzyme R subunit | | **-0.94** | **-0.99** |
| SF5M90T_4279 | *yjiY* | putative carbon starvation protein | | **2.66** | **2.25** |
| SF5M90T_4288 | *hpaD* | homoprotocatechuate dyoxygenase | | **2.27** | **2.07** |
| SF5M90T_4289 | *hpaE* | 5-carboxy-2-hydroxymuconate semialdehyde dehydrogenase | | **2.00** |  |
| SF5M90T_4290 | *hpaG* | 5-oxo-1.2.5-tricarboxilic-3-penten acid decarboxilase/isomerase | | **2.66** |  |
| SF5M90T_4296 | *mdoB* | pseudogene | | **-0.92** | **-1.15** |
| SF5M90T_4300 | *yjjB* | conserved hypothetical protein | |  | **-2.47** |
| SF5M90T_4306 | *fhuF* | ferric hydroximate transport ferric iron reductase | |  | **-1.08** |
| SF5M90T_4312 | *holD* | DNA polymerase III, psi subunit | |  | **-0.81** |
| SF5M90T_4316 | *osmY* | hyperosmotically inducible periplasmic protein | | **4.24** | **4.82** |
| SF4408 |  | conserved hypothetical protein | | **3.66** | **3.40** |
| SF5M90T_4317 | *yjjU* | conserved hypothetical protein | | **1.85** | **1.36** |
| SF5M90T_4318 | *yjjV* | Mg-dependent DNase | | **1.31** | **0.97** |
| SF5M90T_4319 | *yjjW* | putative activating enzyme | | **-3.10** | **-4.33** |
| SF5M90T_4320 | *yjjI* | conserved hypothetical protein | | **-4.87** | **-4.67** |
| SF5M90T_4322 | *deoA* | thymidine phosphorylase | |  | **0.68** |
| SF5M90T_4332 | *slt* | soluble lytic murein transglycosylase | | **1.54** | **1.46** |
| SF5M90T_4339 | *creC* | sensory histidine kinase | | **1.43** |  |
| SF5M90T_4341 | *arcA* | aerobic respiration control protein | | **-1.54** | **-1.11** |
| pWR501_0002 |  | putative resolvase | | **1.97** |  |
| pWR501_0003 |  | hypothetical protein | |  | **3.29** |
| pWR501_0004 | *phoN2* | apyrase | | **2.38** | **3.25** |
| pWR501_0005 |  | hypothetical protein | |  | **2.49** |
| pWR501_0007 |  | hypothetical protein | | **1.63** |  |
| pWR501_0012 | *shET2-2* | enterotoxin | |  | **3.41** |
| pWR501_0013 | *mkaD* | mouse killing factor | | **1.97** | **3.33** |
| pWR501_0014 |  | hypothetical protein | | **1.60** | **1.27** |
| pWR501_0015 |  | hypothetical protein | | **1.52** | **1.05** |
| pWR501_0016 |  | hypothetical protein | |  | **2.32** |
| pWR501_0030 |  | putative enterotoxin fragment | | **2.20** | **4.54** |
| pWR501_0066 |  | hypothetical protein | |  | **2.06** |
| pWR501_0074 | *sepA* | secreted protease | | **1.34** | **1.52** |
| pWR501_0084 | *IpaH7.8* | T3SS effector | |  | **1.37** |
| pWR501_0097 | *ShET2-1* | enterotoxin | | **-1.60** |  |
| pWR501_0100 |  | hypothetical protein | |  | **1.68** |
| pWR501_0103 |  | hypothetical protein | |  | **1.56** |
| pWR501_0105 |  | hypothetical protein | |  | **1.02** |
| pWR501_0113 |  | hypothetical protein | |  | **0.97** |
| pWR501_0114 |  | hypothetical protein | |  | **1.46** |
| pWR501_0115 |  | hypothetical protein | |  | **1.43** |
| pWR501_0122 |  | hypothetical protein | | **1.53** | **1.95** |
| pWR501_0130 | *ipaJ* | invasion plasmid antigen | |  | **0.86** |
| pWR501_0131 | *virB* | transcriptional activator | | **2.40** | **4.26** |
| pWR501_0132 | *acp* | hypothetical protein | |  | **5.19** |
| pWR501_0133 | *ipaA* | T3SS effector | | **3.09** | **4.98** |
| pWR501_0134 | *ipaD* | T3SS effector | | **2.99** | **4.92** |
| pWR501_0135 | *ipaC* | T3SS effector | | **2.65** | **5.01** |
| pWR501_0136 | *ipaB* | T3SS effector | | **2.97** | **4.59** |
| pWR501_0137 | *ipgC* | chaperon | | **1.86** | **4.04** |
| pWR501_0138 | *ipgB* | invasion protein | |  | **3.66** |
| pWR501_0139 | *ipgA* | chaperon | | **2.45** | **4.25** |
| pWR501_0140 | *icsB* | T3SS effector | | **2.16** | **4.43** |
| pWR501_0141 |  | hypothetical protein | |  | **3.71** |
| pWR501_0142 | *ipgD* | secreted protein | |  | **3.90** |
| pWR501_0143 | *ipgE* | chaperon | |  | **3.39** |
| pWR501_0144 | *ipgF* | putative lytic transglycosylase | |  | **3.20** |
| pWR501_0145 | *mxiG* | T3SS component | |  | **3.45** |
| pWR501_0146 | *mxiH* | T3SS component | | **1.55** | **4.10** |
| pWR501_0147 | *mxiI* | T3SS component | | **2.00** | **4.01** |
| pWR501_0148 | *mxiJ* | T3SS component | | **2.17** | **4.33** |
| pWR501_0149 | *mxiK* | T3SS component | |  | **4.33** |
| pWR501_0150 | *mxiL* | hypothetical protein | | **2.07** | **4.64** |
| pWR501_0151 | *mxiM* | T3SS component | | **2.40** | **4.25** |
| pWR501_0152 | *mxiE* | transcriptional activator | | **2.51** | **4.48** |
| pWR501_0153 | *mxiD* | T3SS component | | **2.33** | **4.81** |
| pWR501_0154 | *mxiD* | T3SS component | | **2.66** | **4.54** |
| pWR501_0155 | *mxiC* | T3SS component | | **2.43** | **4.95** |
| pWR501_0156 | *mxiA* | T3SS component | |  | **4.67** |
| pWR501_0157 | *spa15* | chaperon | | **3.52** | **4.43** |
| pWR501_0158 | *spa47* | T3SS component | | **3.19** | **4.69** |
| pWR501_0159 | *spa13* | T3SS component | |  | **5.13** |
| pWR501_0160 | *spa32* | invasion protein | | **1.34** | **4.63** |
| pWR501_0161 | *spa33* | T3SS component | | **1.99** | **4.03** |
| pWR501_0162 | *spa24* | T3SS component | | **2.28** | **3.71** |
| pWR501_0163 | *spa9* | T3SS component | | **1.93** | **3.56** |
| pWR501_0164 | *spa29* | T3SS component | | **2.36** | **3.32** |
| pWR501_0165 | *spa40* | T3SS component | |  | **4.02** |
| pWR501_0166 | *spa-orf10* | hypothetical protein | | **1.97** | **4.52** |
| pWR501_0167 | *spa-orf11* | hypothetical protein | | **2.85** | **4.07** |
| pWR501_0175 |  | hypothetical protein | |  | **2.29** |
| pWR501_0176 |  | hypothetical protein | |  | **2.02** |
| pWR501_0177 |  | hypothetical protein | |  | **1.04** |
| pWR501_0179 |  | hypothetical protein | |  | **-2.61** |
| pWR501_0191 | *virA* | T3SS effector | |  | **2.42** |
| pWR501_0192 | *virG* | invasion protein | |  | **2.36** |
| pWR501_0198 | *ushA* | UDP-sugar hydrolase | |  | **0.92** |
| pWR501_0199 |  | hypothetical protein | |  | **-2.13** |
| pWR501_0200 |  | putative reverse transcriptase fragment | |  | **-1.40** |
| pWR501_0205 |  | hypothetical protein | | **-1.56** |  |
| pWR501_0206 | *stbB* | plasmid stable inheritance protein | | **-3.81** |  |
| pWR501_0207 | *stbA* | plasmid stable inheritance protein | | **-2.52** |  |
| pWR501_0225 | *ospI* | T3SS effector | | **-2.21** |  |
| pWR501_0238 | *mob9* | plasmid mobilization protein | |  | **0.80** |
| pWR501_0250 | *shf* | peptidoglycan deacetylase | | **-3.42** | **-1.30** |
| pWR501_0251 | *rfbU* | glycosiltransferase | | **-1.65** | **-1.16** |
| pWR501_0252 | *virK* | virulence protein | | **-2.16** | **-1.03** |
| pWR501_0253 | *msbB* | acyltransferase | | **-3.03** | **-1.08** |
| pWR501_0264 | *yigA* | hypothetical protein | |  | **0.91** |
| pWR501_0279 | *repA2* | replication protein | |  | **-0.50** |
| pWR501_0280 | *repA1* | replication protein | |  | **-0.59** |
| pWR501_0282 |  | hypothetical protein | |  | **-2.60** |
| pWR501_0290 |  | hypothetical protein | |  | **2.00** |
| pWR501_0292 | *sopA* | VirG-specific protease | |  | **2.49** |
|  |  |  | |  |  |

1. Genomes used as reference are: *S. flexneri* 5a str. M90T, *S. flexneri* 2a str. 301. *S. flexneri* 2002017 and *Shigella* sp. D9 with GenBank accession numbers AGNM00000000, AE005674, CP001383 and NZ_GG657384 respectively. *S. flexneri* 5a str. M90T pWR501 virulence plasmid sequence with GenBank accession numbers AF348706 was also used as reference.
2. Log2 of Fold Change values of Δ*fnr* no O_2_/WT no O_2_ comparisons obtained using RNA-seq and FRT-seq technologies are presented. Only values with *p* adjust <0.05 were considered differentially expressed.

Table S3. Summary of mapping statistics

|  | **WT O_2_^*^** | **Δ*fnr* O_2_^*^** | **WT no O_2_^*^** | **Δ*fnr* no O_2_^*^** |
| --- | --- | --- | --- | --- |
|  |  |  |  |  |
| **RNA-seq** |  |  |  |  |
| Total number of mapped reads | 14652524 | 56780496 | 20099597 | 22736494 |
| Ratio s/as of total mapped reads | 1.84 | 1.17 | 1.80 | 2.15 |
| SD of ratio s/as of total mapped reads | 1.06 | 0.59 | 0.84 | 0.15 |
| Total number of reads mapping to genes | 1529546 | 4516733 | 1525782 | 2271423 |
| Reads mapping genes in sense | 1221664 | 3700589 | 1195446 | 1958533 |
| Reads mapping genes in antisense | 307882 | 816144 | 330336 | 312890 |
| % genes with reads in sense (5 minimum) | 79.67 | 87.36 | 75.31 | 82.98 |
|  |  |  |  |  |
| **FRT-seq** |  |  |  |  |
| Total number of mapped reads |  |  | 49925286 | 47605241 |
| Ratio s/as of total mapped reads |  |  | 1.78 | 1.95 |
| SD of ratio s/as of total mapped reads |  |  | 0.05 | 0.07 |
| Total number of reads mapping to genes |  |  | 3037954 | 2585600 |
| Reads mapping genes in sense |  |  | 2469828 | 2129951 |
| Reads mapping genes in antisense |  |  | 568126 | 455649 |
| % genes with reads in sense (5 minimum) |  |  | 78.09 | 80.82 |

Sense/antisense (s/as). Standard Deviation (SD). *Values shown are the average between replicates.

Table S4. Strains and plasmids used in this study.

| **Strain or plasmid** | **Relevant characteristic(s)** | **Source** |
| --- | --- | --- |
| **Strains** |  |  |
| *E. coli* DH5*α* | Used for cloning assays | Invitrogen |
|  |  |  |
| *S. flexneri* M90T | *S. flexneri* (serotype 5a), nalidixic acid resistant | [[1](#_ENREF_1)] |
| *S. flexneri* M90T *Δfnr* | *S. flexneri* M90T with deletion of *fnr* gene | This study |
| *S. flexneri* M90T *Δfnr* pBM2 | *S. flexneri* M90T *fnr* mutant complemented with pBM2 | This study |
| *S. flexneri* M90T *Δfnr* pBBR1MCS-4 | *S. flexneri* M90T *fnr* mutant transformed with pBBR1MCS-4 | This study |
|  |  |  |
| **Plasmids** |  |  |
| pGEMT | Vector used for cloning assays | Promega |
| pKO3blue | CloR derivative of pKO3 carrying the pMAD *lacZ* gene under the control of the PclpB promoter. Vector used for deletion of genes | [[2](#_ENREF_2)] |
| pBM2 | Derivative of pBBR1MCS-4 carrying a copy of *fnr* gene under the control of its own promoter. Vector used for complementation experiments. | [[3](#_ENREF_3)] |
| pBBR1MCS-4 | Vector used for complementation experiments | [[4](#_ENREF_4)] |
|  |  |  |

Table S5. Oligonucleotides used in this study.

| **Name** | **Sequence (5’-3’)^*^** |
| --- | --- |
| **Mutagenesis** |  |
| *fnr*-A | GCGGCCGCCTTAGCATTATTGATACGCTTC |
| *fnr*-B | CCATGGGTCTGCTCAAGCCGTAATTG |
| *fnr*-C | CCATGGCTGGCAGTCAAAGGTAAATAC |
| *fnr*-D | AGATCTGTGGTCATTTCGTATGAGAAG |
| *fnr*-E | GACAAGCTTCGTGAATATTTTG |
| *fnr*-F | GACGCATAGCGGTACGTTC |
| **DNA control** |  |
| *trpB -*1 | CACTGACCAAATGCCAGAAC |
| *trpB* -2 | CCGGGATCACTTCCGCAC |
| *thrB-*1 | GAGCGTCGGGTTTGATGTG |
| *thrB*-2 | GTTCATTCATCGCCATCAGC |
| *purN-*1 | CGATGTGGTCGTGCTGGC |
| *purN-*2 | CTTTCGCCTGTAAAATAACCG |
| *mdh*-1 | CAGATGTCGTTCTTATCTCTG |
| *mdh-*2 | GGTCAGATCAGCCACTTCC |
| **qRT-PCR** |  |
| Tag primer | **CCGTCTAGCTCTCTCTAATCG** |
| *pol*A-FW | CTGACCTTTAACCAGATTGC |
| *pol*A-RV-T | **CCGTCTAGCTCTCTCTAATCG**CGATATTCTCGAAGACGTTC |
| *ptsG* FW | CGTACCTTTCCAGATGCAGA |
| *ptsG* RV-T | **CCGTCTAGCTCTCTCTAATCG**AGCCACCAGACAGTTTACCC |
| *manX* FW | CATTGCAGGCGTTAACATTC |
| *manX* RV-T | **CCGTCTAGCTCTCTCTAATCG**TTGGCTTTCAGTGCTTTCAC |
| *murQ* FW | TAACGGCACAGGATGTGGT |
| *murQ* RV-T | **CCGTCTAGCTCTCTCTAATCG**ACAGGAAATTCCCACTGTGC |
| *cra* FW | GAAGATCAGCCAGACAACGA |
| *cra* RV-T | **CCGTCTAGCTCTCTCTAATCG**CGACGTCGAAACAATAATGG |
| *gapA* FW | TTCCGTGTTCGATGCTAAAG |
| *gapA* RV-T | **CCGTCTAGCTCTCTCTAATCG**GGAGTAACCGGTTTCGTTGT |
| *cysP* FW | TGACCGACGTACAAATCCTG |
| *cysP* RV-T | **CCGTCTAGCTCTCTCTAATCG**GAACGGCGAGCTATTATTCG |
| *ybgH* FW | ATGTGGCAATCGTCATTTCA |
| *ybgH* RV-T | **CCGTCTAGCTCTCTCTAATCG**GTTCGGCAGGAGAAAGTTTG |
| *adiY* FW | CATGCGTTATGCCGTAAATG |
| *adiY* RV-T | **CCGTCTAGCTCTCTCTAATCG**ACTGTTGTAGCCGCAGGACT |
| *yciD* FW | GTGCTGGTGGTACGTTAGGA |
| *yciD* RV-T | **CCGTCTAGCTCTCTCTAATCG**CTGCCAGTAATTCCACACCA |
| *nmpC* FW | TGGTCTGAACTTTGCTGCTC |
| *nmpC* RV-T | **CCGTCTAGCTCTCTCTAATCG**GAGAAACCGAAACCATCACC |
| *slyB* FW | ACGTCCGGTACAGATTCAGG |
| *slyB* RV-T | **CCGTCTAGCTCTCTCTAATCG**TGCTGCAGTAGCCAGAGAAC |
| *hns* FW | ATGTACACTTGAAACGCTGG |
| *hns* RV-T | **CCGTCTAGCTCTCTCTAATCG**ATTGCTGCAGCTTACGAGTG |
| *stpA* FW | GGGTTGTCACTAAAGAAAGAC |
| *stpA* RV-T | **CCGTCTAGCTCTCTCTAATCG**GCTCCAGCCAGGTGCTAA |
| *fruB* FW | TGAAGCTCGACAACGAAATG |
| *fruB* RV-T | **CCGTCTAGCTCTCTCTAATCG**TCATTGATGGCTTTGGTGAC |
| *udhA* FW | TCTGGAACAGTGGCGTAGTG |
| *udhA* RV-T | **CCGTCTAGCTCTCTCTAATCG**AGAGCAGGCAGTCAGCTTTC |
| *menD* FW | GAACAGGGGCAATTGTTTGT |
| *menD* RV-T | **CCGTCTAGCTCTCTCTAATCG**GAAAGCAGTCCGTCGATACC |
| *ygiP* FW | GGCCTTTAGCCAAAGACTTG |
| *ygiP* RV-T | **CCGTCTAGCTCTCTCTAATCG**AGCACTAAAACTCCCGCTGT |
| *phoN2* FW | GGCTATCACTGGCTCTTATCC |
| *phoN2* RV-T | **CCGTCTAGCTCTCTCTAATCG**TGAGGATTAATCTCCGCAAG |
| *virB* FW | TACGCGATCAAGAATCCCTA |
| *virB* RV-T | **CCGTCTAGCTCTCTCTAATCG**AGTGCCATCCAGAATTTCAA |
| *ipaB* FW | GAGGCGCTCTTGTCCTAGTT |
| *ipaB* RV-T | **CCGTCTAGCTCTCTCTAATCG**ACAGCTGCCAGTGCTAGAGA |
| *mxiH* FW | AAATCCTTCGAATCCACAGTT |
| *mxiH* RV-T | **CCGTCTAGCTCTCTCTAATCG**ATTGCAGCATCAACATCCTT |
| *mxiE* FW | AGCAGGGAGAGAGGCAGA |
| *mxiE* RV-T | **CCGTCTAGCTCTCTCTAATCG**GACTTTAAAGCACACCCACTC |
| *spa32* FW | GTTCGAACTTAGAAACGAACGA |
| *spa32* RV-T | **CCGTCTAGCTCTCTCTAATCG**TTTCCCTTGCTTTGATATCTTG |
| *spa33* FW | ATTCAAGTTTCCCGATGACA |
| *spa33* RV-T | **CCGTCTAGCTCTCTCTAATCG**TAGAAACAAGTTCCCCATGC |
| *virA* FW | CAATTCAAACTTCCCCCAAC |
| *virA* RV-T | **CCGTCTAGCTCTCTCTAATCG**TGCAGGGTCTTGAAAGAGTG |
| *virG* FW | TGGCACTATGGTCACATCCT |
| *virG* RV-T | **CCGTCTAGCTCTCTCTAATCG**TGAGCTCCATTTCCAATTCA |
| *shf* FW | CGGTTTTATTGGGAATGGTC |
| *shf* RV-T | **CCGTCTAGCTCTCTCTAATCG**TTCCCTGTCGCTATTTGATG |
| *msbB* FW | TGGTTTACATGGTTCGTTTTG |
| *msbB* RV-T | **CCGTCTAGCTCTCTCTAATCG**GCCAAGACAGGATCTCTGAA |
| *ospI* FW | TTGGCAGCGACTTTACAGAA |
| *ospI* RV-T | **CCGTCTAGCTCTCTCTAATCG**ACGGGAGCCTCTCTTACTCC |
| *parA* FW | CTCATCCTCACCTGCTGTCA |
| *parA* RV-T | **CCGTCTAGCTCTCTCTAATCG**CCGCATGCAAGTAGTTCAGA |
| *parB* FW | GTCGGCAGCTAAGGTGACTC |
| *parB* RV-T | **CCGTCTAGCTCTCTCTAATCG**GTTGCCTGTAATCGGCGTAT |
| *shiA* FW | ATAACCATTCCTCCGTGCTG |
| *shiA* RV-T | **CCGTCTAGCTCTCTCTAATCG**ACAGGTGCTCCTGCTGTCTT |
| **Northern** |  |
| *csrB* 1 | GACAACGAAGTGAACATCAGG |
| *csrB* T7-2 | **TAATACGACTCACTATAGGG**CCTTGACAACTTTTCCTCTGG |
| *csrC* 1 | CTAACAGGAACAATGACTCAG |
| *csrC* T7-2 | **TAATACGACTCACTATAGGG**CAAGCAAAGAAAAAAGGCGAC |
|  |  |

*Restriction sites are underlined. Tag and T7 promoter sequences are marked in bold.


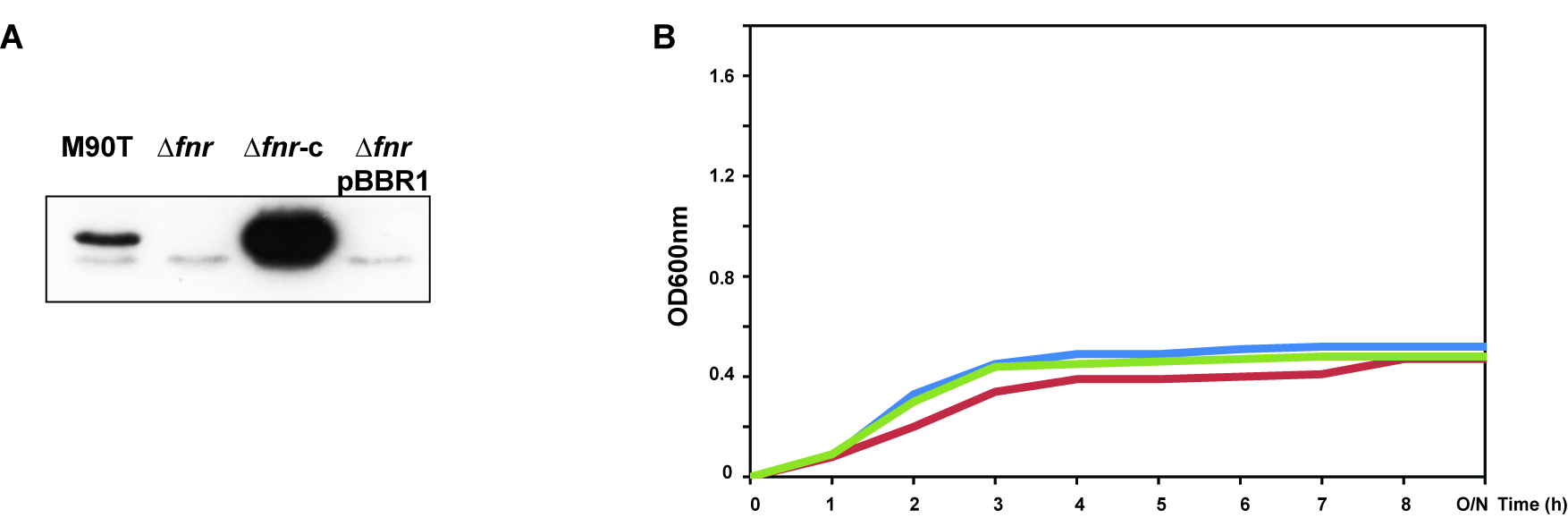


Figure S1. Characterization of M90T Δ*fnr* mutant.

1. Detection of FNR by western blot in the wild-type strain M90T, its isogenic Δ*fnr* mutant, the complemented mutant, Δ*fnr*-c and the control Δ*fnr* strain carrying the empty plasmid pBBR1MCS-4.
2. Growth curve of wild-type strain M90T (blue), its isogenic Δ*fnr* mutant (red) and the complemented Δ*fnr* mutant (green) growing at 37ºC, in LB medium under anaerobic and shaking conditions.

References

1. Sansonetti PJ, d'Hauteville H, Formal SB, Toucas M: **Plasmid-mediated invasiveness of "*Shigella*-like" *Escherichia coli***. *Ann Microbiol (Paris)* 1982. **133**(3):351-355.

2. Solano C, Garcia B, Latasa C, Toledo-Arana A, Zorraquino V, Valle J, Casals J, Pedroso E, Lasa I: **Genetic reductionist approach for dissecting individual roles of GGDEF proteins within the c-di-GMP signaling network in Salmonella**. *Proc Natl Acad Sci U S A* 2009. **106**(19):7997-8002.

3. Marteyn B, West NP, Browning DF, Cole JA, Shaw JG, Palm F, Mounier J, Prevost MC. Sansonetti P, Tang CM: **Modulation of *Shigella* virulence in response to available oxygen *in vivo***. *Nature* 2010. **465**(7296):355-358.

4. Kovach ME, Elzer PH, Hill DS, Robertson GT, Farris MA, Roop RM2nd, Peterson KM: **Four new derivatives of the broad-host-range cloning vector pBBR1MCS carrying different antibiotic-resistance cassettes**. *Gene* 1995. **166**(1):175-176.
